# Hepatoprotective and Anti-fibrotic Agents: It's Time to Take the Next Step

**DOI:** 10.3389/fphar.2015.00303

**Published:** 2016-01-07

**Authors:** Ralf Weiskirchen

**Affiliations:** Institute of Molecular Pathobiochemistry, Experimental Gene Therapy, and Clinical Chemistry, RWTH University Hospital AachenAachen, Germany

**Keywords:** hepatic fibrosis, 3R principle, therapy, animal experimentation, collagen, α-smooth muscle actin, clinical trials, translational medicine

## Abstract

Hepatic fibrosis and cirrhosis cause strong human suffering and necessitate a monetary burden worldwide. Therefore, there is an urgent need for the development of therapies. Pre-clinical animal models are indispensable in the drug discovery and development of new anti-fibrotic compounds and are immensely valuable for understanding and proofing the mode of their proposed action. In fibrosis research, inbreed mice and rats are by far the most used species for testing drug efficacy. During the last decades, several hundred or even a thousand different drugs that reproducibly evolve beneficial effects on liver health in respective disease models were identified. However, there are only a few compounds (e.g., GR-MD-02, GM-CT-01) that were translated from bench to bedside. In contrast, the large number of drugs successfully tested in animal studies is repeatedly tested over and over engender findings with similar or identical outcome. This circumstance undermines the 3R (Replacement, Refinement, Reduction) principle of Russell and Burch that was introduced to minimize the suffering of laboratory animals. This ethical framework, however, represents the basis of the new animal welfare regulations in the member states of the European Union. Consequently, the legal authorities in the different countries are halted to foreclose testing of drugs in animals that were successfully tested before. This review provides a synopsis on anti-fibrotic compounds that were tested in classical rodent models. Their mode of action, potential sources and the observed beneficial effects on liver health are discussed. This review attempts to provide a reference compilation for all those involved in the testing of drugs or in the design of new clinical trials targeting hepatic fibrosis.

## Introduction

**Figure 1:**
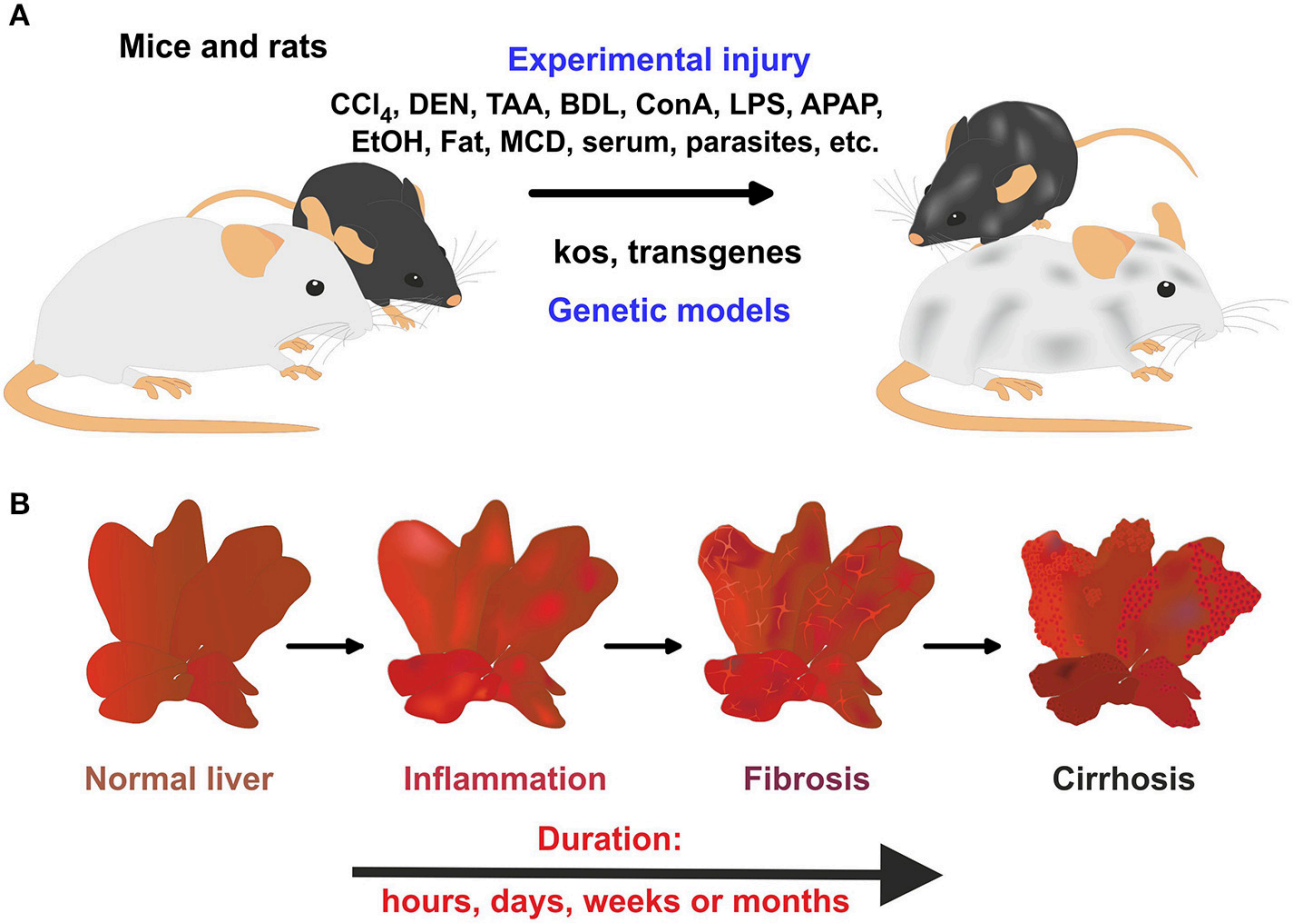
**Rodent models in experimental liver fibrosis. (A)** The application of hepatotoxins or parasites, bile duct surgery, or the feeding of specialized diets is widely applied to induce liver damage and hepatic fibrogenesis in mice and rats. In addition, genetically engineered mice models that develop spontaneous hepatic fibrosis are further alternatives. **(B)** In these models, a time-dependent progress of liver damage occurs in which inflammation, fibrosis, and cirrhosis time-dependently follow each other.

The last statistical report on the number of animals used for experimentation and other scientific purposes in the member states of the European Union was published in 2011 (EU Parliament, 2013). It contains data collected in 26 member states in 2011 and in France in 2010. In summary, this report shows that about 11.5 million animals were used for experimental and other scientific purposes in the EU, of which mice (60.9%) and rats (13.9%) were by far the most used species (EU Parliament, 2013). Over 46% of these animals were used for biological studies of a fundamental nature and 8.75% in the area of toxicology and other safety evaluations. These are particular the two research areas in which novel test drug candidates for future human trials are pre-clinical screened for their safety and efficacy. In hepatology research, there is a mandatory need for novel anti-fibrotic therapies and many different *in vivo* and *in vitro* rodent models were introduced during the last decades (Figure 1). Mice and rats are relatively inexpensive and can be bred in large quantities, their inbred character helps to establish reproducible results, and their anatomy, genetics and biology is similar to humans. Most importantly, the pathogenesis of experimental hepatic disease in rodents closely resembles the disease progress in humans (Friedman, 2008). In this process, hepatic stellate cells (HSC) and portal fibroblasts are major collagen-producing cells. The proliferative activity is triggered by numerous pro-fibrogenic chemokines and cytokines that in liver are produced by residental cells or infiltrating blood cells (Gressner and Weiskirchen, 2006). This complex network of cellular interactions and the great diversity of different mediators offer a wealth of potential drug targets for targeting disease progression.

**Figure 2:**
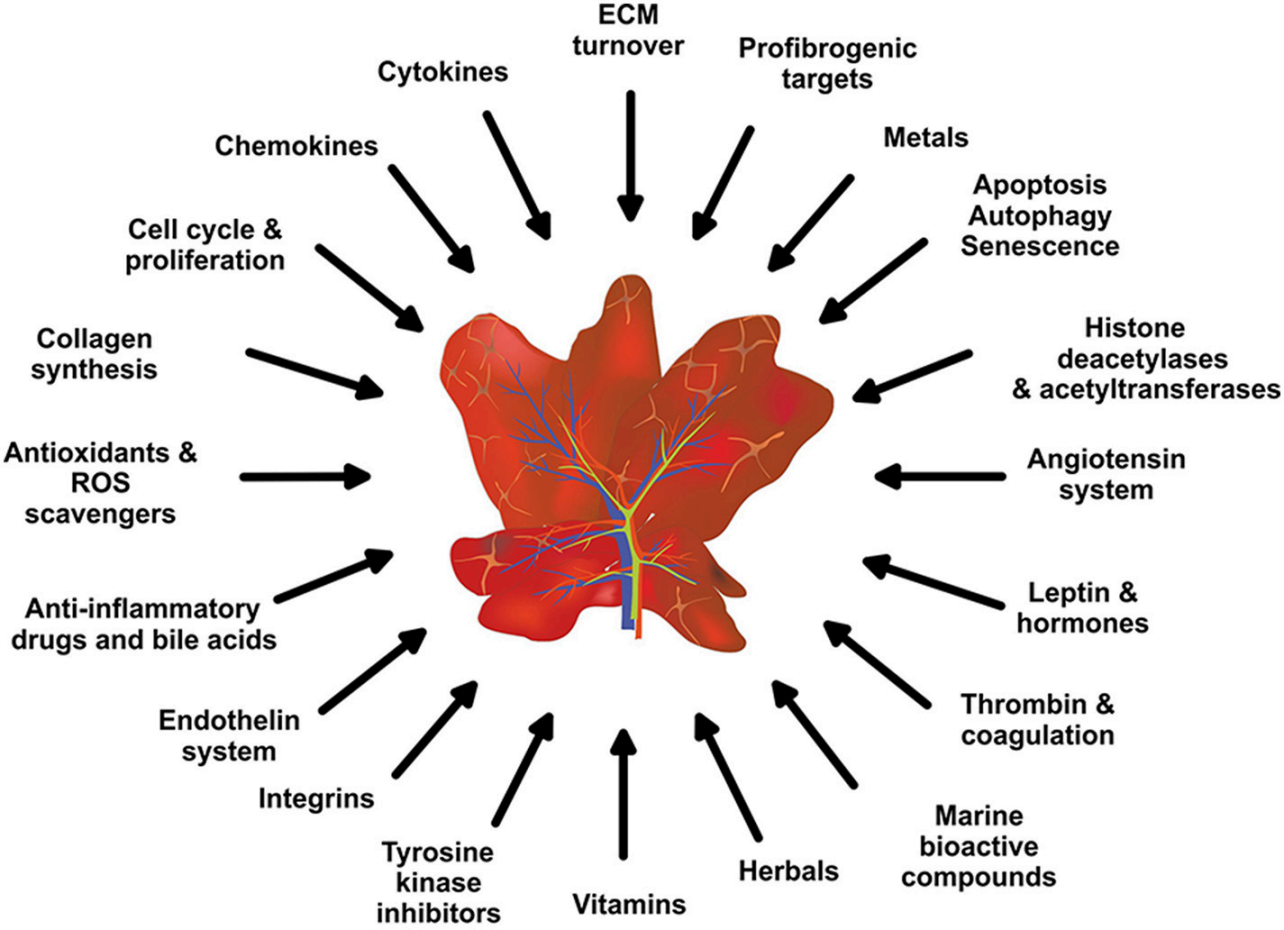
**Potential anti-inflammatory and anti-fibrotic drug targets and treatments**. Based on the complexity of hepatic fibrosis, there are numerous possibilities for therapeutic intervention.

Using pre-clinical rodent models, many hundred (or thousands) pharmacological active ingredients with presumed fibropreventive, fibrostatic, or fibrolytic spectrum were discovered (Figure 2). However, the translation of these encouraging findings to humans and the initiation of human trials is perennially hampered by many factors. Consequently, there are no effective treatments for hepatic fibrosis to date. Instead, many of the identified substances are tested in regular intervals in other cell systems or animal models confirming previous reports. Although the authors of these confirmatory studies will compile some nice publications, the novelty of these studies is rather low. In addition, all these studies are expensive, cause needless pain, and suffering to animals and subvert the ethical framework for conducting scientific experiments with animals that was first proposed by Russell and Burch (1959). These guidelines encourage the replacement, reduction and refinement of animals used for scientific purposes and testing. Currently largely ignored, this so called 3R principle is the basis of the new animal welfare rules that have been implemented in the member states of the EU by the EU Directive 2010/63 and had turned into law at the beginning of year 2013 (EU Parliament, 2010). Nevertheless, in future this regulation predicts that new animal studies initiated with the aim to test drugs that were already tested before will not be approved in the Member states of the EU. Moreover, applying for a new animal study will require a concise review on what is done so far and what was not tested yet.

In the present review, a comprehensive synopsis on experimentally tested anti-fibrotic compounds is given. The chemical structure, potential sources, mode of action, molecular target and their experimental pharmacological activity in hepatic fibrogenesis of each drug is discussed.

## Antioxidants/radical scavengers

Reactive oxygen species (ROS) formation is one key driver of hepatic inflammation and fibrosis. Under normal physiological conditions, oxygen-containing reactive molecules control key physiological activities such as cell growth, proliferation, migration, differentiation, and apoptosis (Manea et al., 2015). However, elevated intracellular ROS concentrations induce damage to cell structures (DNA, RNA, protein, lipids, and cofactors), oxidative stress and inflammation. In the liver, ROS induce apoptosis and necrosis of hepatocytes, stimulate the production of profibrogenic mediators by Kupffer cells and recruitment of circulating inflammatory cells, and leads to direct activation of HSC (Sánchez-Valle et al., 2012). Accumulating evidence suggest that beside multiple other mechanisms, the upregulation of different NADPH oxidases (NOX) subtypes in liver fibrogenesis is majorly the cause for increased intracellular ROS concentrations (Crosas-Molist and Fabregat, 2015; Manea et al., 2015). This assumption was recently confirmed in NOX1- or NOX4-deficient mice (Lan et al., 2015). When these mice were treated with carbon tetrachloride (CCl_4_) to induce liver fibrosis, they showed reduced hepatic inflammation than wild type mice. Moreover, culture-activated HSC derived from these mice had overall reduced expression of pro-fibrogenic genes and the dual NOX1/4 inhibitor GKT137831 suppressed ROS production and expression of inflammation-associated genes (Lan et al., 2015). Intracellular ROS formation also affects the activity of pro-fibrogenic genes and *vice versa*. Of particular interest in hepatic fibrogenesis is the interrelation of ROS and transforming growth factor-β (TGF-β). In cultured HSC, TGF-β increases the production of H_2_O_2_ (De Bleser et al., 1999), which in turn induces the expression of α1(I) procollagen mRNA (García-Trevijano et al., 1999). Consequently, catalase, an enzymatic scavenger of H_2_O_2_, abrogated TGF-β-mediated type I collagen gene expression (García-Trevijano et al., 1999). Numerous agents available prevent or even interfere with ROS formation, or alternatively eliminate or scavenge elevated intracellular ROS traces. Based on their chemical composition, they can be divided into sulfur-containing and non-sulfur containing antioxidants. In the following, some examples of both groups and their beneficial effects in hepatic fibrosis are summarized.

### Glutathione, N-acetyl-L-cysteine, S-Nitroso-N-acetylcysteine, S-adenosyl-L-methionine, S-allylcysteine

There are a large number of sulfur-containing antioxidants with beneficial effects on hepatic inflammation and fibrosis (Supplementary Figure 1). Glutathione (GSH) is an essential nutrient that is synthesized in the body from amino acids L-cysteine, L-glutamic acid, and glycine. It exists in both reduced from (GSH) or in a dimer oxidized (GSSG) form. In the reduced form, the sulfhydryl group of the cysteine residue is able to donate a reducing equivalent and serve as a proton, give rise to its activity as an antioxidant. GSH and its structurally related compounds N-acetyl-L-cysteine (NAC), S-Nitroso-N-acetylcysteine (SNAC), S-adenosyl-L-methionine (SAM) and S-allylcysteine (SAC) have been used in clinics for the treatment of fibrotic diseases. NAC, SNAC, SAM, and SAC either serve as direct GSH precursors, nitric oxide (NO) donors, or serve as methyl group donors required for methylation of nucleic acids, phospholipids, histones, biogenic amines, and proteins. In addition, NAC dose-dependently blocked TGF-β signaling in fibrogenic cells by monomerization of the biological active TGF-β dimer (Meurer et al., 2005). NAC attenuated hepatic oxidative stress and prevented increases in cytochrome P450 2E1 apoprotein, TNF-α expression, and induction of auto-antibodies associated with lipid peroxidation in a dietary polyunsaturated fat model of non-alcoholic steatohepatitis (NASH) in rats (Baumgardner et al., 2008). Likewise, NAC prevented cirrhosis by reducing oxidative stress and TGF-β expression (Galicia-Moreno et al., 2009). The oral SNAC administration resulted in a reduction in collagen α1, increased matrix metalloproteinase (MMP)-13 activity, and a significant suppression of TIMP-2 and TGF-β1 (Mazo et al., 2013). It further induced de-differentiation of the immortalized murine hepatic stellate cell line GRX (Stefano et al., 2011). SAM is a modulator of cellular apoptosis, suppressor of tumor necrosis factor (TNF)-α and inducer of interleukin (IL)-10 expression (Pfalzer et al., 2014). It further inhibits cellular proliferation, adhesion, migration and invasion of human HSC *in vitro* (Zhang et al., 2014). The efficacy of SAC was proven in a porcine serum-induced hepatic fibrosis model in rats in which this compound attenuated hepatic fibrosis and suppressed α-smooth muscle actin (α-SMA) expression (Shinkawa et al., 2009).

### Bucillamine

This substance is a cysteine derivative that contains two donatable thiol groups thereby acting as a potent sulfhydryl donor rendering a potent antioxidant activity. It is particular efficacious in acute settings characterized by inflammation and oxidative stress (Horwitz, 2003). It further sequesters iron (II) and copper (II) that are both involved in oxidative stress-induced damage (Mazor et al., 2006). Bucillamine also inhibits neutrophil activation during hepatic injury and modulates the Bax/Bcl-2 ratio without affecting the tissue GSH levels (Junnarkar et al., 2010).

### Lipoic acid

Lipoic acid (or better α-lipoic acid) contains two sulfur atoms that are connected by a redox-sensitive disulfide bond. It functions as a cofactor for some enzyme systems involved in acyl group transfer. Independent studies have shown that α-lipoic acid and its reduced form dihydrolipoic acid inhibit liver fibrosis in rats chronically treated with thioacetamide (TAA), most likely by preventing ROS generation and ROS-mediated signaling in HSC (Foo et al., 2011). In addition, lipoic acid prevented the development of BDL-induced hepatic fibrosis and effectively attenuated TGF-β-stimulated PAI-1 expression through inhibition of the TGF-β-associated mediators Smad3, AP1, and SP1 (Min et al., 2010). Similarly, this substance prevented fibrosis development, inflammation and cellular apoptosis in rats that were subjected to a high fat diet (Kaya-Dagistanli et al., 2013).

### Taurine

This compound is a derivative of cysteine that is a major constituent of bile and has many biological activities in bile acid conjugation, membrane stabilization and calcium signaling. It further acts as a direct antioxidant and protects against the toxicity of different metals. Taurine administration in drinking water prevented the activity of lipid hydroperoxides, improved mitochondrial enzyme activities, and regulated iron and calcium levels in experimental rat liver fibrosis that was induced by simultaneous application of ethanol and iron (Devi and Anuradha, 2010). In the same model system, taurine lowered the levels of IL-6, TNF-α and peroxidation products, as well as the expression of α-SMA, desmin, and TGF-β1 and further improved the antioxidant status (Devi et al., 2010). Other studies showed that this β-amino acid is neither a classical scavenger nor a regulator of the anti-oxidative defenses but more likely serves as a regulator of mitochondrial protein synthesis (Jong et al., 2012).

### α-tocopherol and trolox

**Figure 3:**
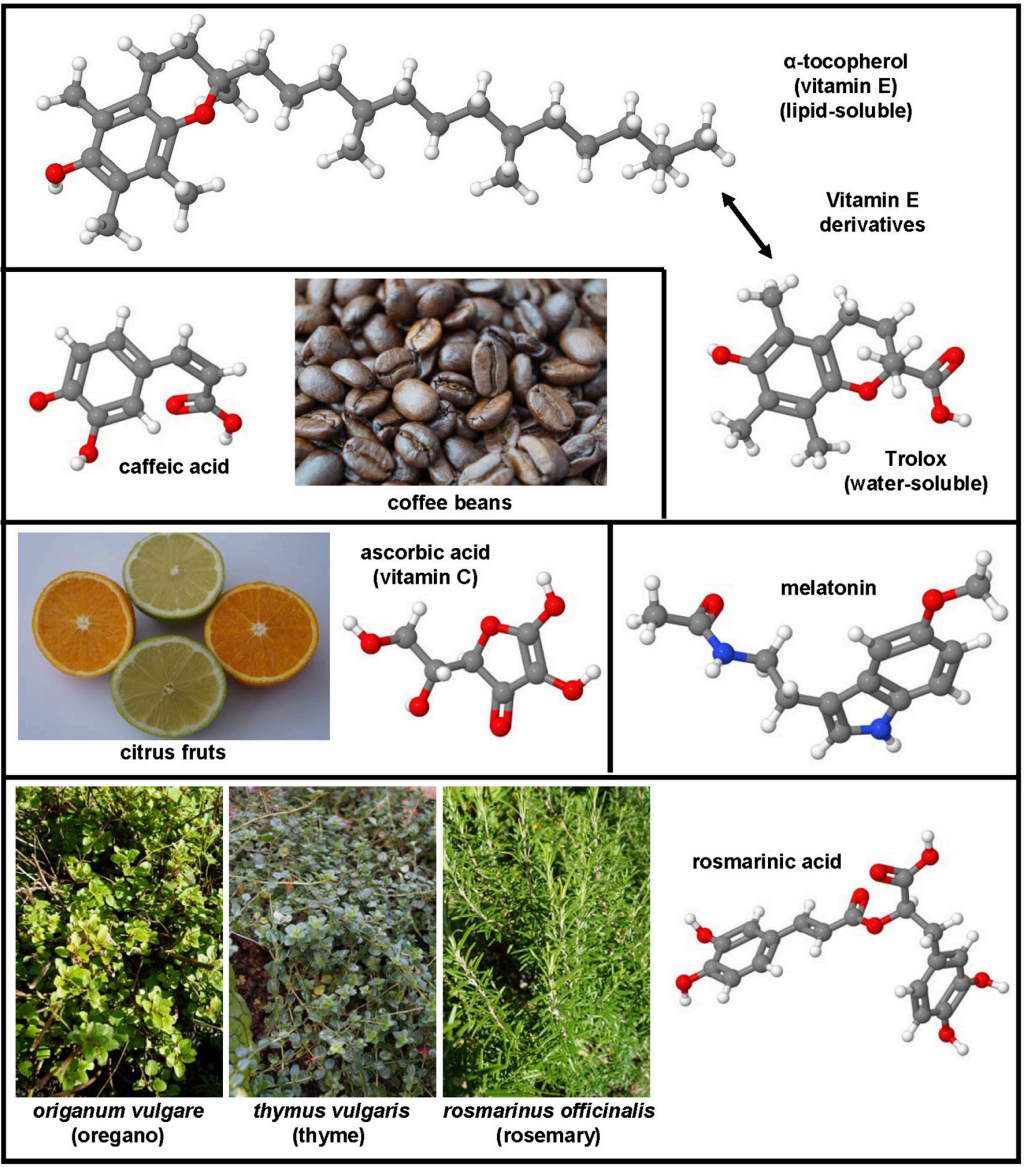
**Non-sulfur-containing antioxidants with therapeutic potential in hepatic fibrosis**. α-tocopherol (CAS 59-02-9), trolox (CAS 53188-07-1), ascorbic acid (CAS 50-81-7), melatonin (CAS 73-31-4), rosmarinic acid (CAS 20283-92-5), and caffeic acid (CAS 331-39-5) were successfully applied in many experimental models of hepatic fibrosis. Major sources of ascorbic acid are citrus fruits, while caffeic acid can be found in high concentrations in coffee beans. Rosmarinic acid is ingredient of culinary herbs such as oregano, thyme, and rosemary.

The non-sulfur-containing lipid-soluble benopyranol α-tocopherol (vitamin E) and its water-soluble analog Trolox are effective peroxyl radical scavengers and absorber of oxygen radicals (Figure 3). Moreover, these substances were found to act as non-competitive inhibitors of cyclooxygenase, suppressor of vascular endothelial growth factor (VEGF) and TGF-β gene transcription (Abdelazim et al., 2015). The importance of the antioxidant capacity of vitamin E in preventing fibrosis was proven in animals studies (Zhang et al., 1996) and clinically confirmed (Harrison et al., 2003).

### Ascorbic acid

This soluble essential nutrient (vitamin C; Figure 3) is an essential cofactor in enzymatic reactions, involved in many biochemical activities, and as a electron donor acting as a direct free radical scavenger (Padayatty et al., 2003). Vitamin C alone or in synergy with other agents decreases lipid peroxidation directly or indirectly by regenerating vitamin E (Adikwu and Deo, 2013). In line with this assumption, the pre-treatment with vitamin C prior exposure to TAA is sufficient to prevent hepatic cirrhosis in rats (Al-Attar, 2011). However, ascorbic acid also induced pro-fibrotic effects in hepatic fibrogenesis in mice that lacked regucalcin (SMP30), a gene that is critically involved in hepatic Ca^2+^ homeostasis (Park et al., 2010).

### Melatonin

This substance, also known as N-acetyl-5-methoxytryptamine (Figure 3), is a free-radical scavenger and antioxidant that protects against nuclear and mitochondrial DNA damage and interacts with the immune system. It has also the capacity to form complexes with different metals. In dimethylnitrosamine (DMN)-induced liver fibrosis in rats, melatonin functions as a potent fibrosuppressant and antioxidant by preventing the decrease of GSH and superoxide dismutase levels (Tahan et al., 2004). This substance has recently renewed interest because it is an effective drug that inhibits autophagy, necroptosis, and endoplasmic reticulum stress in CCl_4_-induced hepatic fibrosis in mice (San-Miguel et al., 2015, p. 38; Choi et al., 2015). In the same model, it attenuated liver injury and inhibited the expression of collagens types I and III, TGF-β, PDGF, connective tissue growth factor (CTGF), amphiregulin, and activation of Smad3, while the MMP-9 activity decreased and the expression of nuclear factor erythroid-2-related factor 2 (Nrf2), representing is a central regulator of anti-oxidative response, increased (Crespo et al., 2015).

### Caffeic acid, rosmarinic acid

The natural phenol caffeic acid (Figure 3) is present in modest concentration in coffee. However, it is unrelated to caffeine and is composed of a phenol ring carrying an unsaturated carboxylic acid side chain. Like its ester, rosmarinic acid, caffeic acid has a high rate constant to react with hydroxyl radicals (Bors et al., 2004). Rosmarinic acid (Figure 3) inhibited proliferation and induced apoptosis in HSC-T6, partly due to the inhibition of phosphorylation in STAT3 (Zhang et al., 2011b). The intraperitoneal application of phenethyl ester of caffeic acid was recently shown to evolve hepatoprotective effects and to suppress HSC activation by inhibiting oxidative stress in rats that were injected subcutanly with CCl_4_, feed with high fat forage, and administered with alcohol orally (Li et al., 2015b).

### Genistein, luteolin, quercitin, apigenin, naringenin, and other polyphenolic compounds

**Figure 4:**
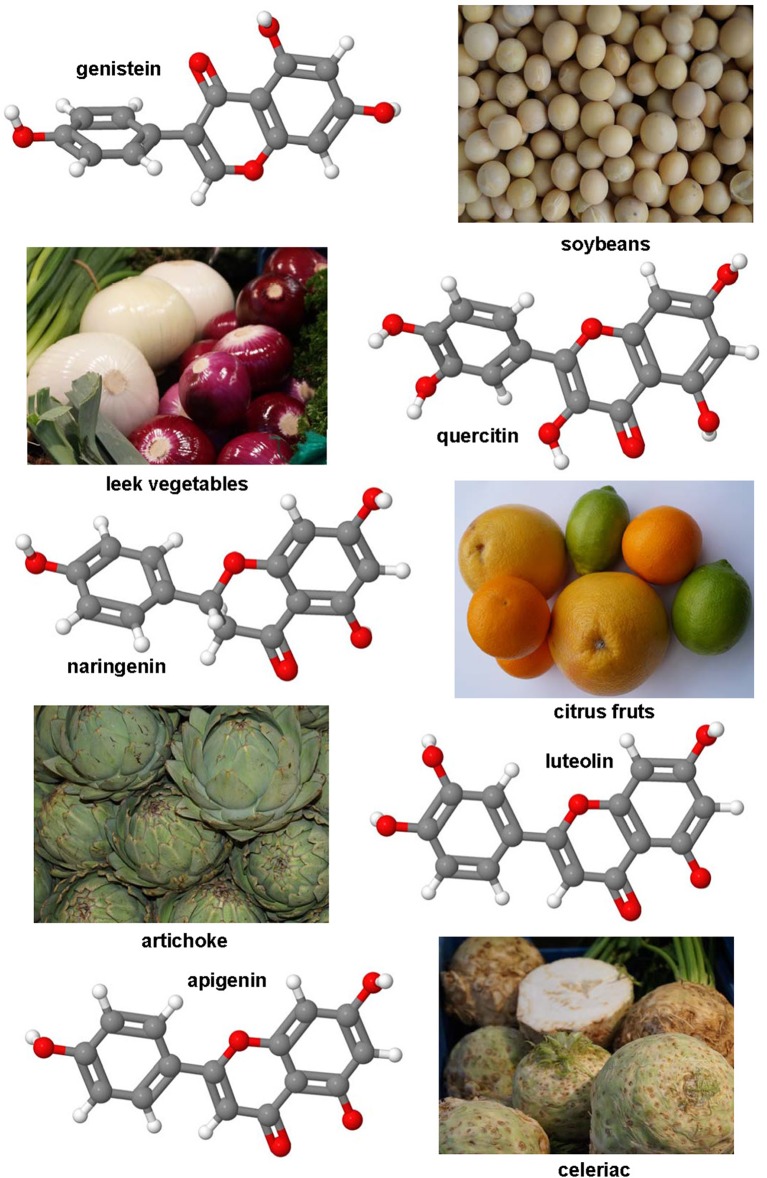
**Polyphenol substances as therapeutics in hepatic fibrosis**. Genistein (CAS 446-72-0), luteolin (CAS 491-70-3), quercitin (CAS 117-39-5), apigenin (CAS 520-36-5), and naringenin (CAS 480-41-1) share a similar polyphenolic structure with ability to scavenge free radicals. Fruits or vegetables in which these substances occur are depicted besides the formulas.

Genistein is a naturally occurring phytoestrogic isoflavone that is present in high concentration in soy and many other plants. Similar to many other polyphenolic substances (Figure 4), this compound acts as a direct antioxidant. It reduces damaging effects of free radicals, prevents the release of cytochrome c from mitochondria and has further the ability to modulate the activity of the nuclear receptor Peroxisome proliferator-activated receptor (PPAR)-γ and the estrogen receptor (Dang et al., 2003; Borrás et al., 2010). Dietary supplementation of genistein down regulated the augmented gene expression associated with hepatic inflammation and fibrosis in a methionine-choline-deficient (MCD) diet in leptin receptor deficient db/db mice (Yoo et al., 2015). Likewise, genistein ameliorated developing liver injury and fibrosis that was induced by repeatedly intragastric administration of alcohol (Huang et al., 2013). Luteolin is a flavone that is found in high concentrations in leaves of the yellow myrobalan (*Terminalia chebula*), avocado, celery, olive oil, chamomile, peppermint and many aromatic plants. It was supposed that this compound and some of its derivatives act as dopamine transporter activators (Zhang et al., 2010). Although the precise activity is not fully understand, this drug increased the expression of MMP-9 and Metallothionein thereby promoting extracellular matrix (ECM) degradation in established hepatic fibrosis that was induced by administration of CCl_4_ (Domitrović et al., 2009). More recently, it was demonstrated that luteolin inhibited DEN-initiated alcohol-promoted hepatic inflammation by stimulating hepatic sirtuin 1 activity that is a master regulator in hepatic lipid metabolism (Rafacho et al., 2015). Other reports have suggested that luteolin prevents progression of liver fibrosis through a multitude of different mechanisms that include inhibition of fibrosis-related genes in HSC, induction of HSC apoptosis and cell arrest, and inhibition of cytokine signaling pathways (TGF-β and PDGF; Li et al., 2015a). Quercitin is a pentahydroxyl flavonoid occurring for example in onions, apples, broccoli and green beans. In CCl_4_-treated rats, this substance prevented oxidative stress, lipid peroxidation and increased levels of GSH, SOD, catalase, GPx, and GST levels (Amália et al., 2007). Similarly, quercitin decreased ALT, AST, GGT and LDH levels and increased expression of albumin in a high fat diet model in rats (Surapaneni and Jainu, 2014). This drug was also effective in a MCD diet model in mice in which it attenuated pro-fibrotic and pro-inflammatory gene expression (Marcolin et al., 2012). Strong antioxidant activity, anti-apoptotic and hepatoprotective effects were also reported for the flavone apigenin that occurs in garden parsley (*Petroselinum crispum*), celery (*Apium graveolens*), and different chamomiles. The efficacy of this drug was proven in different toxic liver injury models (Tsalkidou et al., 2014; Wang et al., 2014). Naringenin occurs in high concentrations in grapefruit and other citrus fruits in form of conjugated glucosides such as naringenin-7-rhamnoglucoside or naringenin-7-glucoside. The oral administration of naringenin prevented DMN-induced hepatic fibrosis in rats (Lee et al., 2004a). A subsequent study *in vitro* study performed in HSC-T6 cells showed that naringenin dose-dependently exerts its anti-fibrogenic activity by down-regulation of Smad3 protein expression and activation (Liu et al., 2006). Furthermore, this substance reduced the plasma fat and the hepatic expression of pro-inflammatory mediators such as TNF-α, IL-6, IL-1β, iNOS, MMP-2, and MMP-9 in rats that were fed with a high cholesterol diet (Chtourou et al., 2015). Likewise, there are many other natural plant-derived polyphenolic compounds with anti-oxidative activity including acids (3,4-OH-benzoic, gallic, O-, and P- coumaric, syringic, vanillic), alcohols (tyrosol and OH-tyrosol), theobromine, rutin, catechine, and apigenin. All these substances are in the pipeline of researchers and are worth to be tested in experimental models of hepatic fibrogenesis.

### Nicotinic acid, nicotinamide, and nicotinamide adenine dinucleotide (phosphates)

Nicotinic acid (niacin) and its amide (vitamin B3) serve as the most fundamental nicotinamide adenine dinucleotide precursors (Supplementary Figure 2). They are critical cofactors in a wide variety of intracellular oxidation-reduction reactions and serve as antioxidants. Nicotinamide adenine dinucleotides consist of either oxidized or reduced unphosphorylated (NAD^+^ or NADH) and phosphorylated (NADP^+^ and NADPH) forms. These compounds are indispensable for a multitude of dehydrogenase enzymes, act as mitochondrial electron carriers and electron donors for GSH, thioredoxin, and NADPH oxidases (i.e., the NOX enzymes). They further act as ADP donors in ribosylation reactions, substrates for NAD^+^-dependent enzymes (e.g., PARPs and Sirtuins), and redox regulators that modify ion channel function (Nakamura et al., 2012). Since the cellular concentration of oxidized and reduced forms of these intracellular ROS modulators is itself influenced by countless factors (e.g., total ROS concentration, enzymatic activities of ROS producers and scavengers), this axis offers a plenitude of possibilities to modulate the intracellular ROS concentration. In TAA-induced hepatic fibrogenesis, the co-administration of nicotinic acid prevented fibrosis by its antioxidant properties and reduction of TGF-β expression (Arauz et al., 2015). As mentioned above, NOXs play crucial roles in hepatic fibrogenesis and HSC express a non-phagocytic form of NOX, which plays a critical role in activating fibrosis-associated pathways (Bataller et al., 2003; De Minicis et al., 2010). The deficiency of NOX1 and NOX4 in CCl_4_-treated mice reduced liver injury, inflammation, and activation of stellate cell thereby attenuating fibrosis (Lan et al., 2015). The importance of NOX1 and NOX4 in HSC was further underpinned by the fact that the dual NOX1/4 inhibitor GKT137831 (Supplementary Figure 2) not only suppressed ROS production but also prevented HSC activation by inhibition of inflammation- and proliferation-associated signaling (Lan et al., 2015). Likewise, the application of the coumarin derivative decursin that blocks the expression and activity of NOX1, NOX2, and NOX4 reduced the quantities of ROS and fibrogenesis in a CCl_4_-induced liver injury model in mice (Choi et al., 2014). Another possibility to reduce the hepatic ROS content and fibrogenesis is the overexpression of the ubiquitously expressed thioredoxin. This protein acts as an antioxidant by facilitating the reduction of other proteins by cysteine thiol-disulfide exchange. In line with this attributed, thioredoxin transgenic mice were protected against TAA-induced hepatic fibrogenesis and HSC isolated from these mice were less proliferative than those isolated from wild type littermates (Okuyama et al., 2005).

## HMG-CoA reductase inhibitors

The central and rate-controlling enzyme in the synthesis of cholesterol is the 3-hydroxy-3-methyl-glutaryl-CoA (HMG-CoA) reductase that NADH- or NADPH-dependently forms mevalonate from HMG-CoA. There are a large number of drugs, i.e., the statins, which inhibit the activity of this enzyme. A growing number of statins such as lovastatin, atorvastatin, simvastatin, pravastatin, fluvastatin, pitavastatin, rosuvastatin, and others (Supplementary Figure 3) and combinations thereof are on the market. They should prevent cardiovascular disease in liver-diseased patients (Argo et al., 2008). Atorvastatin attenuated ongoing and established hepatic fibrogenesis in BDL rats by inhibiting HSC activation or turnover suggesting that this statin has both protective and therapeutic potential (Trebicka et al., 2010). In accordance with its therapeutic potential, it was shown that atorvastatin decreased cytokine and collagen production in myofibroblasts (MFB) *in vitro* and initiated apoptosis (Klein et al., 2012). Anti-proliferative activity in cultured primary HSC was also demonstrated for lovastatin and simvastatin that both inhibited proliferation and collagen expression (Rombouts et al., 2003). Rosuvastatin successfully improved hepatic steatosis in a high-fat and high-cholesterol diet-induced NASH model in rats and further improved hepatic fibrosis *via* improved peroxisomal β-oxidation (Okada et al., 2013). Simvastatin administered intragastrically in a model of high fat diet in rats significantly reduced expression of inducible nitric oxide (iNOS) synthase, α-SMA, TGF-β1, and collagen, while inducing the expression of endothelial nitric oxide (eNOS) synthesis that evolves protective function in the cardiovascular system (Wang et al., 2013). However, there are an increasing number of reports that somewhat questioning the beneficial effects of statins in therapy of hepatic disease. In a BDL model performed in rats, the administration of rosuvastatin in early stages of cholestasis decreased α-SMA expression and inhibited NF-κB activation but also increased hepatocytolysis, oxidative stress formation, and hepatic inflammation and sustained increased levels of TGF-β1 (Olteanu et al., 2012). Likewise, in the TAA model in rats both atorvastatin and rosuvastatin failed to inhibit liver cirrhosis or oxidative stress formation and had no effect of HSC proliferation (Shirin et al., 2013).

## Hepatoprotective substances affecting fat metabolism

Excessive alcohol consumption, high caloric intake, or several metabolic disorders predispose for fatty liver in which triglycerides accumulate in liver cells. This process termed steatosis is accompanied by inflammation that on long term ends in fibrosis. Therefore, substances that prevent intracellular fat uptake or increase the turnover and metabolization of triglycerides have anti-inflammatory and anti-fibrotic impact.

### L-carnitine

**Figure 5:**
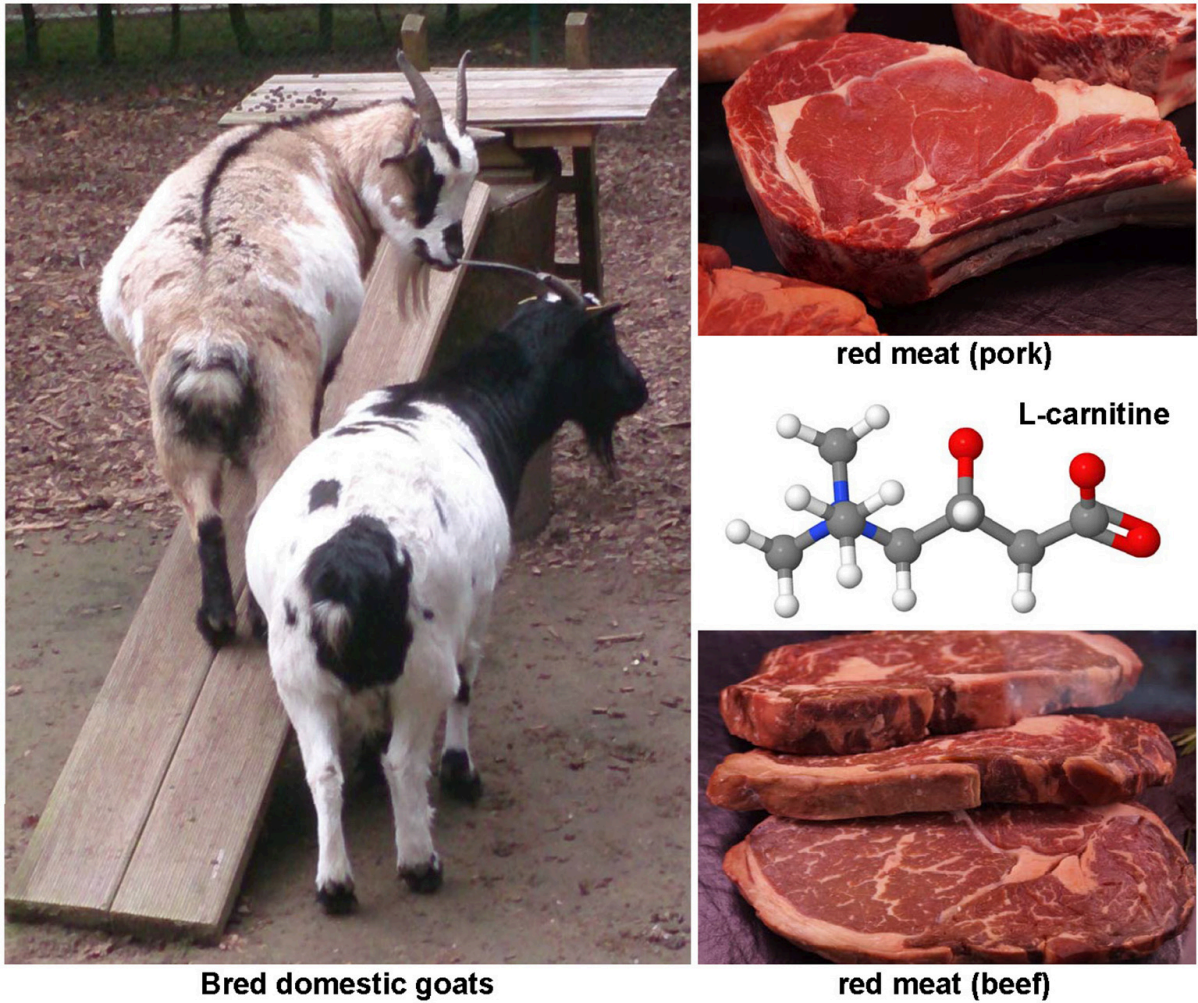
**The hepatoprotective agent L-carnitine**. This quaternary ammonium compound (CAS 541-15-1) is enriched in meats of mutton, lamb and other mammals. It has essential function in long-chain fatty acid transport, prevent mitochondria dysfunction, and reduces expression of TNF-α, PDGF, and IL-6.

Carnitine (Figure 5) is found in highest concentrations in red meat and is structurally a quaternary ammonium compound with essential function in long-chain fatty acid transport. It acts as a fatty acid carrier across the mitochondrial membrane. The protective effects of carnitine were demonstrated experimentally in CCl_4_-treated rats in which this substance reduced expression of TNF-α, platelet-derived growth factor (PDGF)-BB and IL-6 (Demiroren et al., 2014). Other animal studies have shown that the dietary supplementation with L-carnitine protects mitochondria and influences the outcome of chemically induced hepatitis and subsequent hepatocellular carcinoma (HCC), as well as NASH-related HCC (Chang et al., 2005; Ishikawa et al., 2014).

### Polyenylphosphatidylcholine (PPC)

This highly purified mixture of different phospholipids is marked as “Essentiale” with indications in acute and chronic hepatitis, fatty degeneration, toxic liver damage and dyslipoproteinemia. This preparation should evolve several hepatoprotective activities such as recovery of hepatocytes, improvement of lipid and glycogen metabolism, correction of mitochondrial failure and activation of RNA synthesis. Its main phosphatidylcholine species dilinoleoylphosphatidylcholine is highly effective in blocking TGF-β1-induced collagen and TIMP-1 expression in rat HSC, while Palmitoyl-linoleoylphosphatidylcholine, the second most abundant component in PPC had no effect on expression of these pro-fibrogenic genes (Cao et al., 2002).

## Hepatoprotective substances affecting sugar metabolism

Elevated fasting glucose is a risk factor for non-alcoholic fatty liver disease that is associated with NASH, fibrosis, and cirrhosis. Insulin resistance and diabetes contribute to the progression from NASH to fibrosis through the development of a pro-fibrotic environment in the liver (Chiang et al., 2011). Therefore, drugs with insulin-sensitizing or anti-hyperglycaemic activity are one option that protects for hepatic inflammation and fibrosis. Several drugs are available that either has one or both of these activities.

### Pioglitazone (actos) and rosiglitazone (avandia)

Based on its insulin-sensitizing and anti-hyperglycaemic activity, this drug is clinical used for the treatment of adult-onset diabetes. Chemically, it belongs to the large group of thiazolidinediones also known as glitazones (Supplementary Figure 4) that activate the nuclear PPAR receptors. In experimental hepatic steatosis and fibrosis in rats induced by feeding of a choline-deficient L-amino-acid-defined diet, pioglitazone reduced the expression of TIMP-1, TIMP-2, and prevented the activation of HSC (Kawaguchi et al., 2004). These experimental findings were also confirmed in a rat model of high fat-induced steatosis in which pioglitazone reduced excess hepatic fatty degeneration and fibrosis, serum levels of transaminases, triglycerides, free fatty acids, glucose, insulin, and expression of hepatic collagen I and α-SMA (Zhang et al., 2012b). In humans, a detailed meta-analysis of pioglitazone activity in 137 patients that suffered from NASH showed that patients taken this drug had significant lower grade of fibrosis, lower body weight fat and improvement of ballooning degeneration, lobular inflammation, and steatosis than the placebo group that contained 134 individuals (Boettcher et al., 2012). However, in some rat models the therapeutic anti-fibrotic efficacy of this thiazolidinedione was limited. It failed to interrupt progression of BDL-induced fibrosis suggesting that the etiology leading to fibrosis, the duration of the underlying liver disease, and/or the severity of fibrosis at the time of initiation of piaglitazone treatment significantly influence the therapeutic potential this drug (Leclercq et al., 2006). Rosiglitazone, another thiazolidinedione, showed highly beneficial effects in prevention and ameliorating nutritional fibrosing steatohepatitis in rodents (Nan et al., 2009). In contrast this drug on long-term id associated with increased expression of pro-inflammatory genes in humans that however has no effects on collagen I or TGF-β expression (Lemoine et al., 2014). In summary, all these findings indicate that not all glitazones might be entirely suitable for treatment of NASH or uniformly useful in other kind of hepatic injuries.

## Inhibitors of cytokine signaling

**Figure 6:**
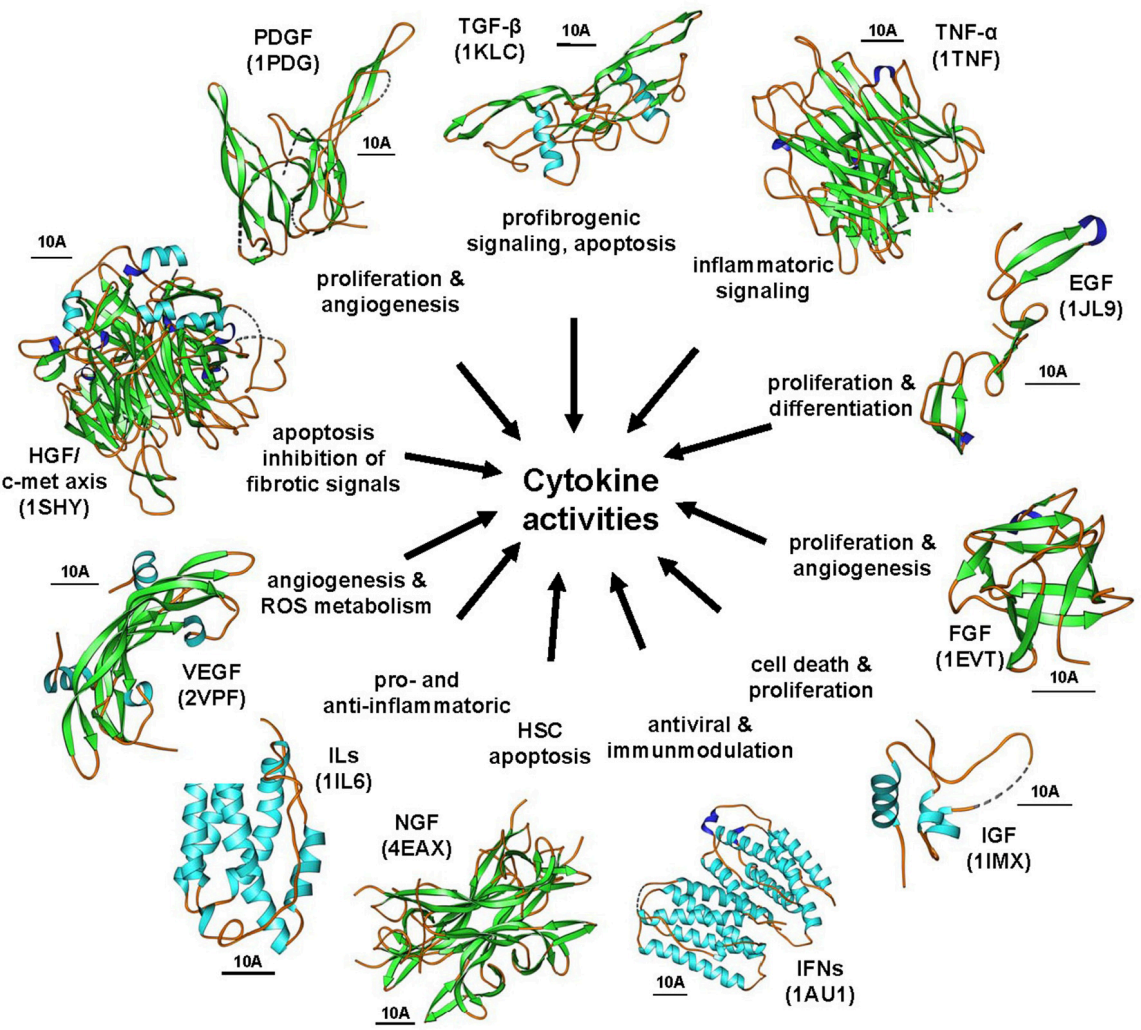
**The cytokine signaling wheel in hepatic fibrogenesis**. Hepatic inflammation and fibrosis is the consequence of multiple cytokine activities that have multiple biological functions. Representative cytokines involved in disease process are TGF-βs, PDGFs, TNF-α, EGFs, FGF, IGFs, IFNs, NGFs, ILs, VEGFs, and the HGF/c-met axis. The images were prepared with Ribbons vers. 3.0 using the structure coordinates deposited in the RCSB Protein Data Base under accession no. 1KLC (human TGF-β1), 1TNF (human TNF-α), 1JL9 (human EGF-1), 1EVT (human FGF1), 1IMX (human IGF-1), 1AU1 (human IFN-β), 4EAX (mouse NGF), 1IL6 (human IL-6), 1SHY (human β-chain of HGF in complex with the sema domain of the c-met receptor), and 1PDG (human PDGF-BB), respectively. The bars represent each 10 A.

Cytokines are produced by a broad range of cells. They and act in an autocrine or paracrine manner by binding to specific cell-surface receptors thereby initiating intracellular signaling cascades commonly resulting in modulation of gene transcription. Over the last decades, there has been increasing knowledge on the involvement of different cytokines and their pathways in the pathogenesis of hepatic fibrosis (Figure 6). Some cytokines that seems to be extremely important for initiation and progression of hepatic fibrosis represent good drug targets that were comprehensively explored during the last decades.

### TGF-β

Transforming growth factor (TGF-β) and its intracellular signaling mediators, the Smad proteins, is the key cytokine axis driving collagen gene transcription. Most obvious the important role of TGF-β in collagen synthesis was shown in mice that carried a fusion gene consisting of modified porcine TGF-β1 cDNA directed under regulatory control of the mouse albumin gene promoter. Respective mice developed hepatic fibrosis and apoptotic death of hepatocytes (Sanderson et al., 1995). *Vice versa*, the suppression of TGF-β expression in ongoing hepatic fibrosis in rats effectively attenuated hepatic fibrogenesis (Arias et al., 2003). Similar results were obtained with soluble TGF-β receptors that sequester biological active TGF-β, dominant-negative TGF-β receptors, dominant negative Smad proteins, or in experiments in which inhibitory Smad7 was expressed (Gressner et al., 2002). Nowadays, novel small inhibitors (such as SB-431542, GW788388, SKI2162, EW-7197, SM16) that specifically target the TGF-β type I receptor (ALK5) that mainly drives the fibrogenic responses in HSC are experimentally tested in mice and rats (de Gouville et al., 2005; Gellibert et al., 2006; Park et al., 2015a,b). In addition, the adenoviral overexpression of BMP-7 that physiologically opposes TGF-β activities was beneficial in experimental models (Kinoshita et al., 2007).

### PDGF

The five members of the PDGF family of cytokines, PDGF-AA, PDGF-BB, PDGF-AB, PDGF-CC, and PDGF-DD, mediate their signals *via* two types of receptor tyrosine kinases (PDGFRα, PDGFRβ) that can form pure or mixed dimers. In general, PDGFs are potent mitogens that drive cell proliferation and differentiation. Their involvement in the pathogenesis of hepatic fibrosis was proven in transgenic mice that expressed PDGF-B under control of the hepatocyte specific albumin promoter (Czochra et al., 2006). This study further revealed that the PDGF-B-induced process of fibrogenesis does not require upregulation of TGF-β suggesting that PDGF-B alone is able to initiate hepatic fibrosis by TGF-β-independent mechanisms. Likewise, the overexpression of PDGF-C is already sufficient to induce hepatic fibrosis in mice, irrespectively if this cytokine is expressed transient or stable (Campbell et al., 2005). Conversely, the lack of PDGF-C in mice is not suitable to prevent hepatic fibrosis suggesting that antagonizing strategies for PDGF-C are not effective to treat liver fibrosis (Martin et al., 2013). During inflammatory liver insult, the expression of different ligands and receptors of the PDGF network is differentially regulated (Borkham-Kamphorst et al., 2008). Most of the experimental studies suggest that the targeting of PDGF-B, PDGF-D, and PDGFRβ should be more effective in treatment of hepatic fibrosis, while the ligands that are more connected to the PDGFRα signaling branch are more relevant in tumor angiogenesis and maintenance of the tumor microenvironment that is necessary for progression to hepatocellular carcinoma (Oseini and Roberts, 2009). In this regard, it is worth to mention that PDGF-B and PDGF-D show the same fibrotic activities in HSC and portal MFB (Borkham-Kamphorst et al., 2015a). In line with these findings, the overexpression of a dominant-negative soluble PDGFRβ blocked HSC proliferation and hepatic fibrogenesis in rats (Borkham-Kamphorst et al., 2004). Today are also varieties of small components available that (more or less) specifically target the PDGFR kinase activity. Some *in vitro* and *in vivo* studies have reported beneficial effects of the quinoxaline-type tyrphostin AG-1295 in hepatic fibrosis.

### TNF-α

Tumor necrosis factor-α (TNF-α) is the prototype of the TNF family that consists of 19 different proteins sharing a homotrimeric or heterotrimeric structure. TNF-α is mainly secreted by macrophages and binds with different affinities to two receptors (TNF-R1, TNF-R2), that initiate several intracellular cascades leading to activation of NF-κB. This pathway triggers cellular activation, differentiation, cytokine production and cellular apoptosis. The activation of TNF-α-associated pathways is causatively linked to liver injury and hepatic inflammation. This connection is particularly demonstrable after Concanavalin A injection that induces acute hepatitis (Heymann et al., 2015). There is an infinite number of studies that unanimously demonstrated that the blockade of TNF-α is suitable to attenuate the inflammatory response. Monoclonal antibodies (e.g., infliximab, adalimumab, certolizumab pegol, golimumab) and circulating receptor fusion proteins (e.g., etanercept) that block TNF signaling are already applied in humans. Although these drugs are beneficial to reduce hepatic inflammation in many disease models, some of these compounds cause different forms of hepatic injury indicated by elevation of serum aminotransferases, induction of auto-antibodies, cholestasis, and reactivation of hepatitis viruses (Efe, 2013).

### NGF

Nerve growth factor (NGF) binds to different receptors (p75NTR, TrkA) and leads to activation of several pathways (e.g., AKT/PKB, MAPK, NF-κB). During fibrotic liver injury, hepatocytes express NGF that dose-dependently leads to increase in HSC apoptosis (Oakley et al., 2003), while proNGF protects MFB from apoptosis (Kendall et al., 2009). These findings suggest that the NGF axis might be of particular interest for therapeutic targeting of HSC apoptosis. It will be interesting to follow how NGF, proNGF, monoclonal antibodies against NGF, non-peptidic NGF agonists and other drug systems that target the NGF route will modulate experimental hepatic fibrosis.

### VEGF

As the term vascular endothelial growth factor (VEGF) already suggest, the five members of this cytokine family (VEGF-A, VEGF-B, VEGF-C, VEGF-D, and PIGF) are important for vascular development and angiogenesis. They bind to tyrosine kinase receptors (VEGFRs) that trigger multiple downstream signals. It is generally assumed that VEGF-A is implicated in hepatic fibrosis. This growth factor is induced in cells upon oxygen tension. It modulates the density of microvessels and is involved in the production of portal hypertension (Corpechot et al., 2002). However, studies that are more recent have shown that VEGF is not only a promoter of hepatic fibrosis but is *vice versa* also required for hepatic tissue repair and fibrosis resolution (Yang et al., 2014c). Therefore, modulation of VEGF or VEGFR activity might provide some drug targets for therapy of hepatic fibrosis. There are several (pan-specific) tyrosine kinase inhibitors (e.g., PTK787/ZK22258, sunitinib, sorafenib, ramucinumab, vatalanib, brivanib) on the market that were already successfully tested in experimental rodent models.

### ILs

Over 40 different cytokines form the group of ILs. Together with their different types of receptors, they possess a pleiotropic activity in the innate and adaptive immune response and in all kinds of inflammatory responses. Beside their linkage to hepatic inflammation, several studies showed that some ILs (e.g., IL-22) have hepatoprotective and anti-fibrotic effects. Therefore, several clinical studies actually examine their effects in liver disease. The high number of ILs and their receptors as well as the availability of IL agonists or antagonists offers infinite therapeutic modalities. Since ILs may evolve pro- or anti-inflammatory activities (as well as both activities), it is most likely that strategies targeting one particular IL or a specific IL receptor will be beneficial only in selected disease subsets. A comprehensive summary of experimental studies in mice targeting different ILs is given elsewhere (Hammerich and Tacke, 2014).

### Fibroblast growth factors

The 22 members of the fibroblast growth factors (FGFs) family bind to five distinct receptors (FGFR1-4, FGFRL1). Signaling of these cytokines is mediated through several downstream pathways including the Ras-Raf-mitogen-associated protein kinase and phosphoinositol-3 kinase-AKT cascades. The FGF family is involved in the initiation of fibrosis and progression of hepatic fibrosis to cirrhosis (Cheng et al., 2011). Mice that lacked FGFR4 were more prone to hepatic fibrosis, while mice that lacked FGF1 and FGF2 showed decreased liver fibrosis in the CCl_4_ model (Yu et al., 2002, 2003) These unexpected and somewhat contradictory findings suggest that FGF signaling offers some potential therapeutic clues for prevention or treatment of hepatic fibrosis.

### HGF

Hepatocyte growth factor (HGF) or scatter factor binds to the c-Met receptor that possesses tyrosine kinase activity. This cytokine has pleiotropic effects on liver cells and influences cell proliferation, apoptosis, differentiation, motility, invasion and angiogenesis. In experimental liver disease, HGF attenuated liver fibrosis (Xia et al., 2006). Mechanistically, it was supposed that HGF suppresses pro-fibrogenic signaling *via* induction of galectin-7 that can bind to phosphorylated Smad2/3 thereby preventing its transcriptional regulator function (Inagaki et al., 2008). The pleiotropic activities of this cytokine combined with the availability of novel inhibitory peptides, therapeutic antibodies, truncated antagonistic peptides (e.g., HGF/NK1), and small molecule inhibitors (e.g., INC280, Tivantinib) that target c-Met will open new avenues for anti-fibrotic treatment strategies.

### EGF

The epidermal growth factor (EGF) induces on its receptor (EGFR) tyrosine kinase activity. This cytokine has recently attracted much interest because EGFR inhibition by erlotinib was shown to attenuate liver fibrosis and development of HCC in DEN-treated mice and BDL rats (Fuchs et al., 2014). EGF and FGF2 synergistically suppressed expression of α-SMA and reversed human activated primary HSC into a transitional state demonstrating that this cytokine counteracts profibrogenic signaling (El Taghdouini et al., 2015).

### IFN

The type I (IFN-α, IFN-β, IFN-ω, IFN-τ, IFN-κ, IFN-λ, IFN-σ) and type II (IFN-γ) interferons (IFN), and their receptors (IFNR) transduce signals *via* the classical JAK-STAT pathway and mediate antiviral and growth-inhibitory effects. Their important immunomodulatory activity alone (for example of pegylated IFN-γ) or in combination with other antiviral drugs is already clinical exploited in treatment of hepatitis virus associated liver disease since many years (EASL, 2015).

### IGF

The two insulin-like growth factors (IGF-1, IGF-2), their six binding proteins (IGFBP-1 to IGFBP-6) and their two receptors (IGF1R, IGF2R) form a complex network, i.e., the “IGF axis.” This axis is critically associated with the development of liver disease. In accordance with this assumption, the treatment with recombinant human IGF-1 attenuated and reversed the fibrotic degeneration of hepatic tissue (Bonefeld and Møller, 2011). Although the importance of the IGF axis is somewhat fallen into oblivion during the last years, there are reasonable arguments that still make IGF and its signaling pathways attractive for therapeutic interventions.

## Inhibitors of chemokine signaling

Chemokines are a large family of chemotactic and immunomodulatory molecules that act through 19 known G-protein coupled receptors. Based on their chemical structure and the spacing of common four conserved cysteine residues, the different chemokines are grouped into four groups, i.e., CXC, CC, CX_3_C, and XC. Most chemokines act as chemoattractant that become released by different cell types. They modulate the biological attributes of immune cells and some chemokines promote hepatic fibrosis. A summary of chemokines and receptors relevant in the pathogenesis of hepatic disease was recently published (Marra and Tacke, 2014). A wealth of studies suggested that the different chemokine-receptor axes offer a large variety of therapeutic options. Neutralizing antibodies to a specific chemokine or its receptor, inactive chemokines blocking receptor docking sites, small molecule receptor antagonists, intracellular signaling blockers, or direct blockade of chemokine synthesis by siRNA or aptamers are currently under investigation. Several clinical trials are on the way testing the efficacy of chemokine antagonism in patients with chronic liver inflammation and fibrosis (Marra and Tacke, 2014). In particular, several 50–120 kDa polysaccharides (e.g., GR-MD-02, GM-CT-01) are promising drugs (Supplementary Figure 5). These compounds target galectin-3 that regulates TGF-β driven HSC activation and inflammation-associated chemoattraction (Henderson et al., 2006; Klyosov et al., 2012). The mentioned polysaccharides showed good beneficial effects on regression of fibrosis and reversal of cirrhosis in rats (Traber et al., 2013). With one of these compounds, GR-MD-02, a large phase 2 trial for the treatment of liver fibrosis and resultant portal hypertension in patients with NASH cirrhosis is currently implemented.

## Modulators of cellular apoptosis, autophagy and senescence

Programmed cell death (apoptosis) and autophagy (autophagocytosis) are physiological programs having fundamental function during development and differentiation. However, enhanced rates of apoptosis are also physiologically important when aberrant cellular proliferation occurs or unwanted cells should be removed. Similarly, exposure to tremendous factors can induce a cellular senescence response in which the growth of premalignant cells is haltered and overshooting cell proliferation in tissue repair prevented. Since the pathogenesis of hepatic fibrosis and cirrhosis is associated with a significant increase in ECM producing cells, it is obvious that the targeted induction of apoptosis, autophagy, or senescence in ECM-producing cells might be therapeutically beneficial. The development of respective strategies targeting profibrogenic cells in hepatic fibrosis is therefore a very important therapeutic option.

### Gliotoxin

**Figure 7:**
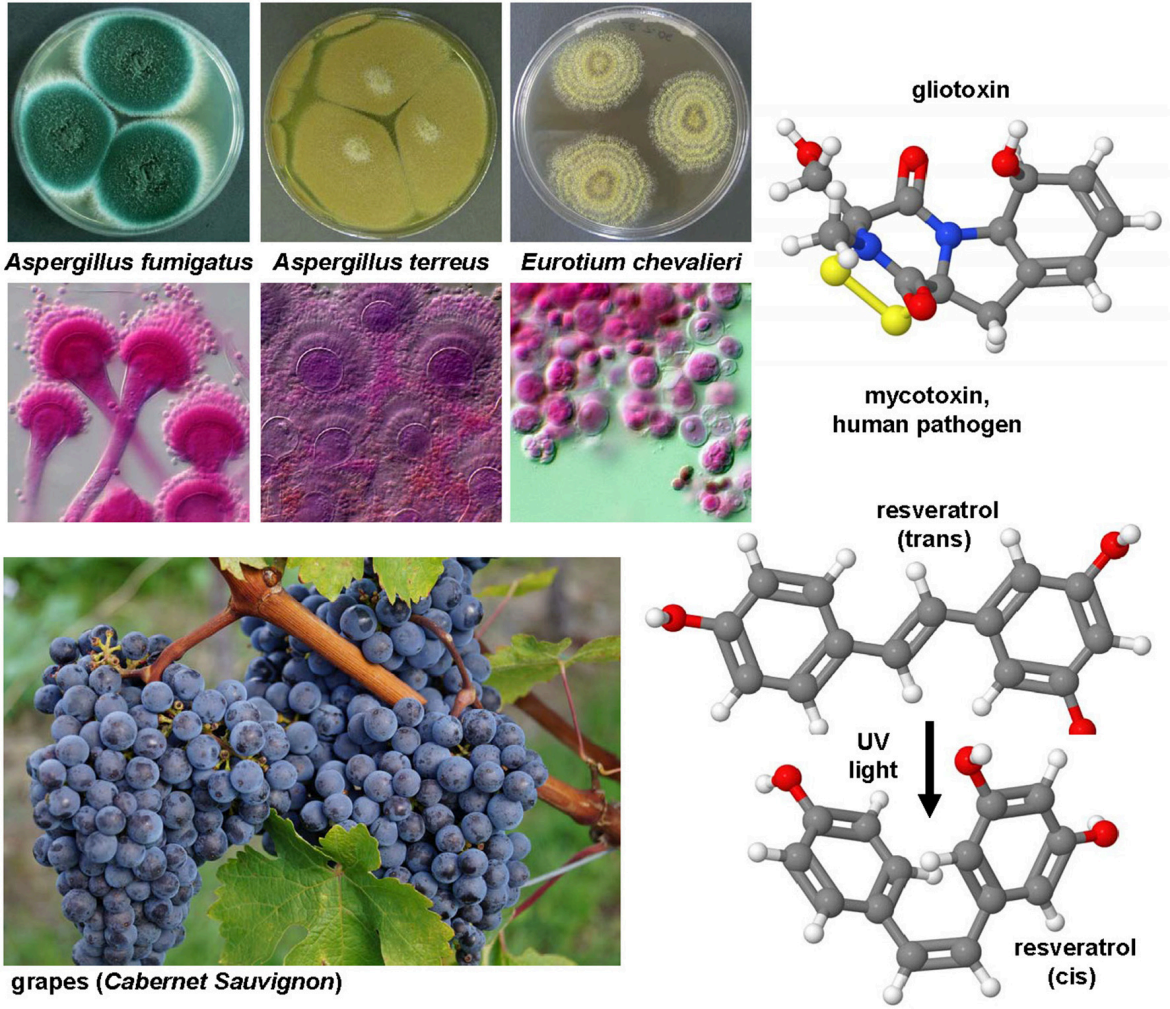
**Gliotoxin and Resveratrol, two modulators of cellular apoptosis, autophagy and senescence**. The potent sulfur-containing mycotoxin gliotoxin (CAS 67-99-2) that can be isolated from many fungi and the stilbenoid resveratrol (trans-resveratrol: CAS 501-36-0, cis-resveratrol, CAS 61434-67-1) that acts as an phytoalexin have been the subject of numerous experimental studies on hepatic fibrosis. Both substances have growth inhibitory activity and apoptotic capacity. In UV light, resveratrol can be converted form its trans into its cis form. The images of *Aspergillus fumigatus, Aspergillus terreus, Eurotium chevalieri* were kindly provided by the CABI/Royal Botanic Gardens, Kew, Richmond, UK and printed with kind permission of Dr. Paul F. Cannon.

*Aspergillus fumigatus* and many other fungi (Figure 7) produce this highly potent mycotoxin. The toxicity if this epidithiodioxopiperazine-type fungal toxin is majorly due to the unusual intra-molecular disulfide bridge. Gliotoxin induced apoptosis in rat and human HSC (Wright et al., 2001) potentially through a specific thiol redox-dependent interaction with the adenine nucleotide transporter in respective cells (Orr et al., 2004). It further reduced the number of activated HSC in the liver of rats treated with CCl_4_ or TAA (Wright et al., 2001; Dekel et al., 2003). Although hepatocytes are rather robust against this drug, all non-parenchymal cells showed a similar degree of apoptosis when tested in normal and fibrotic precision-cut rat liver slices (Hagens et al., 2006). Therefore, gliotoxin might be a future good drug candidate when strict targeting strategies for HSC become available. Although such targeting strategies are under development and evaluation, this drug is presently only of experimental interest.

### Resveratrol

This compound (Figure 7) is a hydroxylated stilbene belonging to the phytoalexins produced by many plants (e.g., grapes, raspberries, mulberries, plums, peanuts). It acts as an antioxidant and anti-mutagen, most likely by inhibition the activity of cyclooxygenase and hydroperoxidase (Jang et al., 1997). First reports in 2000 investigated potential anti-fibrotic effects of resveratrol and analogs in human MFB that were obtained by outgrowths from non-tumoral liver explants (Godichaud et al., 2000). This study revealed that resveratrol dose-dependently inhibited proliferation of MFB and decreased expression of α-SMA and collagen type I without affecting vimentin expression. Moreover, the migration of MFB was inhibited and the secretion of MMP2 increased by resveratrol. All these activities were specific for resveratrol and not found in cells that were stimulated with piceid or piceatonnol representing glucosylated or hydroxylated analogs of resveratrol. Therapeutic effects were found in the CCl_4_ model in mice in which resveratrol prevented the toxin-induced glycogen decrease, expression of TGF-β and further reduced the activity of the NF-κB pathway (Chávez et al., 2008). Similar findings were obtained in the TAA model in which the daily oral gavage of resveratrol for seven days after a single intraperitoneal injection of TAA prevented body and liver weight loss, liver inflammatory infiltrate, and hepatic expression of fibrosis-related genes (Hong et al., 2010). In this model, the authors also found that resveratrol decreased the level of malondialdehyde, while the levels of glutathione peroxidase and superoxide dismutase were increased. A therapeutic action of resveratrol was also proven in murine models of chronic and acute hepatic iron overload (Das et al., 2015). Mechanistically, a very recent study demonstrated that resveratrol induces autophagy, promotes cell death and concomitant apoptosis in GRX cells (Meira Martins et al., 2015).

## Interfering with pro-fibrogenic target molecules

Numerous profibrogenic proteins are known that drastically affect the outcome of hepatic fibrosis. Prototypically for these proteins are some matricellular proteins that are increased expressed during progression of hepatic disease. They modulate either the activity of profibrogenic cytokines or the process of pathologic tissue remodeling. Some examples of this class of proteins are briefly discussed in the following.

### SPARC

The secreted protein acidic and rich in cysteine (SPARC) that is also known as osteonectin is a 40-kDa calcium binding protein that binds to collagen, increases the production and activity of MMPs, modulates cell shape and disrupts cell-matrix interactions. The major source of SPARC in fibrotic livers are HSC, while other hepatic cell types produce only trace amounts of this matricellular protein (Frizell et al., 1995). Adenoviral overexpression of a SPARC antisense was suitable to attenuate the development of hepatic fibrosis in rats in which fibrosis was induced by TAA application (Camino et al., 2008). Subsequent *in vitro* studies performed in immortalized human LX-2 and rat CFSC-2G cell lines showed that the suppression of SPARC prevents HSC adhesion to fibronectin, expression of TGF-β and collagen as well as TGF-β- and PDGF-induced cell migration (Atorrasagasti et al., 2011). In line with these anti-fibrotic capacity of SPARC, mice that lacked SPARC had reduced hepatic collagen deposits combined with elevated quantities of MMP-2 and reduced expression of TGF-β1 when subjected to TAA or BDL (Atorrasagasti et al., 2013). Therefore, it is temping to speculate that SPARC is a potential drug candidate for therapeutic intervention.

### Osteopontin

This highly negative charged 33-kDa ECM protein is also known as bone sialoprotein-1 (BSP-1) or secreted phosphoprotein 1 (SPP1), In HSC it is involved in the control of migration, activation of MMP2, and production of collagen and TGF-β type II receptor (Lee et al., 2004b). Based on these effects it was proposed that osteopontin is a cytokine with key activities in the extracellular protein network (Urtasun et al., 2012). This notion was underpinned by the finding that osteopontin-deficient mice were more susceptible to CCl_4_. It was therefore suggested that the increase of this protein protects for fibrosis (Lorena et al., 2006). In addition, it was demonstrated that osteopontin deficiency is protective in ischemia-reperfusion hepatic injury in mice, most likely by preventing hepatocyte death and sensitizing macrophages to inflammatory signals (Patouraux et al., 2014). Also the overall quantities of osteopontin impacts the resolution of hepatic fibrosis, since osteopontin deficient mice that were made fibrotic by TAA administration showed significant delays in fibrosis resolution when compared to wild type mice (Leung et al., 2013). However, in a murine model of alcoholic neutrophilic hepatitis, the lack of osteopontin failed to prevent alcoholic hepatitis, enhanced the expression of IL-17, and increased the number of polymorphonuclear cells infiltrating the liver potentially pointing to a more complex function of Osteopontin in formation of liver insult (Lazaro et al., 2015).

### CCN2/CTGF and CCN1/CYR61

The CCN (CYR61, CTGF, and NOV) protein family consists of six conserved small secreted cysteine-rich proteins. These matricellular proteins have a modular structure with up to four individual modules including an insulin-like growth factor binding domain, a von Willebrand Factor type C motif, a thrombospondin type I module, and a carboxyl-terminal cystine knot. The individual domains of the CCN proteins confer different binding activities, intrinsic activities, modulator activities, and antagonistic activities (Weiskirchen, 2011). Under normal conditions the different CCN proteins take over important roles in embryonic development, cellular commitment, tissue differentiation (angiogenesis, osteogenesis), and matrix remodeling as well as wound healing. The most intensively studied member of this family in experimental and human liver disease is CCN2/CTGF. Its expression in liver tissue correlates well with the extent of liver injury. The biological inactivation of CCN2/CTGF by antisense nucleotides (Uchio et al., 2004), small interfering RNA (Li et al., 2006, 2008a; George and Tsutsumi, 2007; Georges et al., 2007), short hairpin RNA (Yuhua et al., 2008) or hammerhead ribozymes (Gao and Brigstock, 2009) abrogated the process of ongoing hepatic fibrogenesis *in vitro* and *in vivo*. In this regard, it is noteworthy that CCN2/CTGF binds to and increases the biological activity of TGF-β for the type II TGF-β receptor complex (Abreu et al., 2002). Interestingly, the expression of CCN2/CTGF upon stimulation with TGF-β1 was only marginally increased in primary HSC but strongly increased in primary hepatocytes suggesting that profibrogenic activities of CCN2/CTGF are stimulated in a paracrine fashion (Gressner et al., 2007). In contrast, the basal expression of CCN1/CYR61 in primary hepatocytes is rather low compared to HSC and portal MFB (Borkham-Kamphorst et al., 2014). In models of ongoing liver fibrogenesis, elevated levels of CCN1/CYR61 were particularly noticed during early periods of insult, while its expression declined during prolonged phases of fibrogenesis (Borkham-Kamphorst et al., 2014). Since the delivery of recombinant protein in mice with established fibrosis accelerated fibrosis regression, it was speculated that this CCN protein family might have therapeutic potential by its capacity to induce cellular senescence in hepatic MFB (Kim et al., 2013).

### Tenascins, syndecans, aggrecan, lumican, and fibromodulin

Another family of ECM proteins that is associated with hepatic fibrogenesis are tenascins. This family contains four members (TNC, TNR, TNX, and TNW) sharing multiple EGF-like repeats and several fibronectin-III domains. It is assumed that tenascins have affinity for fibronectin thereby blocking its interaction with syndecans that are heparin sulfate and chondroitin sulfate coupled single transmembrane proteins. TNC is upregulated during chronic hepatic disease and mice that lacked TNC were more prone to hepatic inflammation and fibrosis (El-Karef et al., 2007). In addition, the expression of other keratin sulfate proteoglycans such as syndecans, aggrecan, lumican, and fibromodulin were linked to the pathogenesis of hepatic disease and activation of HSC (Gressner et al., 1994). Although this class of proteins were somewhat forgotten, it is obvious that they have large potential as therapeutic targets in hepatic fibrosis.

### Thrombospondins

The family of thrombospondins are implicated in multiple biological processes. In particular, the finding that thrombospondin-1 is involved in the conversion of latent to activated forms of TGF-β family members has attracted much interest (Schultz-Cherry and Murphy-Ullrich, 1993). Application of a specific tetra-peptide (Leu-Ser-Lys-Leu) derived from the latency-associated peptide of TGF-β prevented the progression of hepatic fibrosis in a DMN model in rats through inhibition of TGF-β1 activation and its downstream signaling (Kondou et al., 2003).

### WNT, hedgehog and notch signaling compounds

The canonical Wnt/β-catenin signal pathway forms a close signaling network with TGF-β thereby influencing liver fibrosis by modulating HSC activation and survival. There are a variety of Wnt pathway antagonists and inhibitors which provide a high variability for targeting the respective pathways. They include soluble receptors, siRNA, chemically synthesized transcription factor competitors and transcription inhibitors (Guo et al., 2012). However, there is presently a strong debate if the Wnt/β-catenin pathway is beneficial or tremendous for the outcome of hepatic fibrosis. While the blockade of Wnt/β-catenin signaling inhibited HSC activation (Ge et al., 2014), the restoration of Wnt/β-catenin signaling in alcohol liver diseased rats attenuated progression of hepatic insult (Huang et al., 2015). However, certainly the Wnt/β-catenin axis still provides potential targets attractive for diagnosis, prognosis, and development of therapeutics (Monga, 2015).

The hedgehog pathway with its three homologs DHH, IHH, and SHH and their receptors is involved in fate determination of HSC and significantly participates in liver fibrosis (Yang et al., 2014a). Several of its antagonists and agonists of the underlying pathways are already in clinical use. Many different agents (e.g., IP-926, LDE-225, Gli-3, arsenic trioxide) can target the activity of the receptor Smoothened.

Notch signaling is a crucial determinant of cell fate decision that becomes visible during liver regeneration and repairs. Alterations in Notch signaling are associated with liver malignancies and it was therefore supposed that the underlying pathways would provide novel drug targets to develop safe and specific therapeutic agents for different hepatic lesions (Morell and Strazzabosco, 2014).

## Modulators of cell cycle or proliferation

### Colchicine, colchiceine

**Figure 8:**
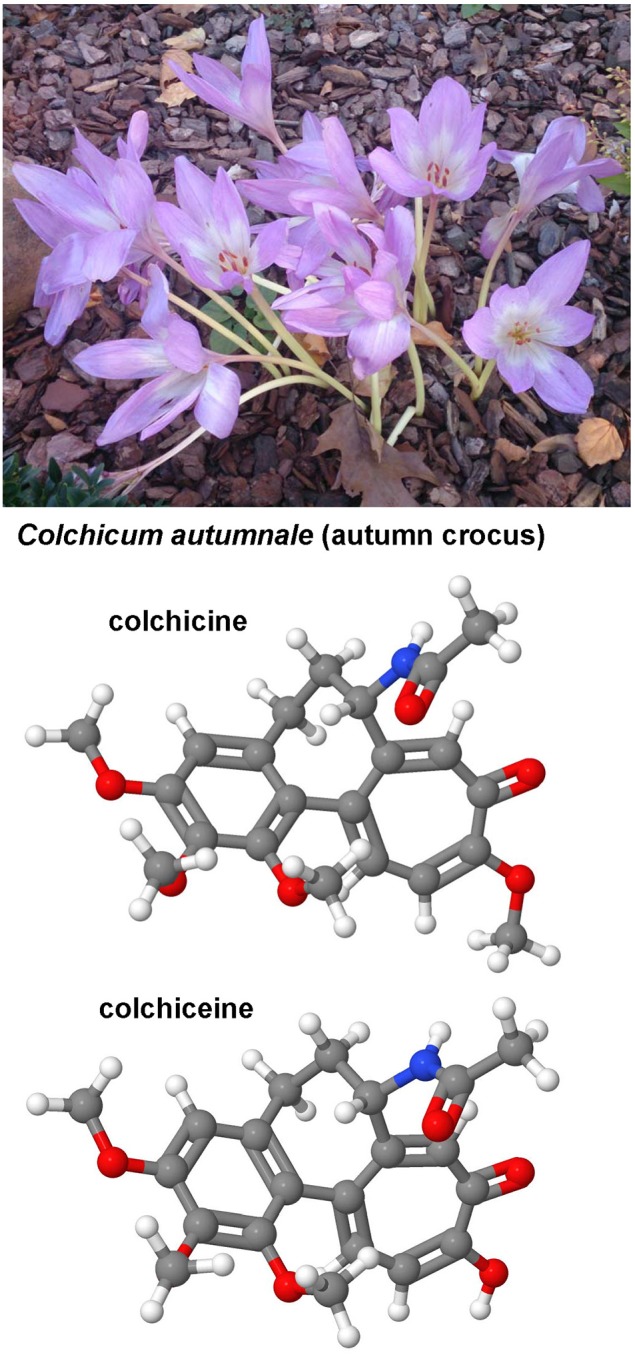
**Colchicine and cholchiceine**. Both colchicine (CAS 64-86-8) and its metabolite colchiceine (CAS 477-27-0) that are found in the autumn crocus have been reported as antifibrotic agents in experimental models of hepatic fibrosis.

Both the toxic *Cholchicum autumnale* alkaloid colchicine and its metabolite colchiceine (Figure 8) are inhibitors of microtubule polymerization by binding to tubulin. Cholchicine is used primarily in the treatment of gout and experimentally as a strong inhibitor of mitosis in genetic karyotyping studies. The interest in colchicine in hepatic fibrosis research arouse in 1975 when Rojkind and Kershenobich found that the content of collagen synthesis was blocked and liver function greatly improved in cirrhotic rat livers (Rojkind and Kershenobich, 1975). A similar protective activity of cholchicine was demonstrated in BDL rats that received chronic oral administration of this drug (Poo et al., 1993). A randomized, double-blind, placebo-controlled trial that was performed in 100 cirrhotic patients arising form diverse causes and followed for 14 years showed that the overall survival rate in the cholchicine group was markedly better (Kershenobich et al., 1988). However, this finding was not reproduced in another trial that enrolled patients with alcohol-induced cirrhosis that received colchicine (*n* = 274) or placebo (*n* = 275) for 2–6 years (Morgan et al., 2005). The potential of this drug is not exhaustive clarified and there are still appearing human studies showing effective and safe anti-fibrotic benefits of this drug (Muntoni et al., 2010). Possibly, the application of colchiceine that has better anti-fibrotic properties than cholchicine in experimentally induced CCl_4_-induced cirrhosis in rats (Nava-Ocampo et al., 1997) and has overall lower toxic effects than cholchicine (Dvorak et al., 2002) is worth to be tested in future clinical studies.

## Modulators of ECM turnover and collagen synthesis

It is obvious that alterations in ECM synthesis, breakdown, or posttranslational modifications of ECM proteins are involved in both initiation and progression of hepatic fibrosis (Karsdal et al., 2015). The composition of the ECM is majorly regulated by proteases and their inhibitors that control ECM remodeling (the MMP/TIMP system) or by enzymes that are directly involved in the synthesis, organization or crosslinking of ECM components (Lysyloxidase and Lysyloxidase like 2). The understanding of ECM remodeling during hepatic fibrogenesis has lead to the uncovering of many novel therapeutic targets. Some of these are briefly discussed in the following paragraphs.

### MMPs and TIMPs

In general hepatic fibrosis is associated with an unbalanced expression of matrix metalloproteinases (MMPs) and their physiological inhibitors, the tissue inhibitors of MMPs (TIMPs). The family of MMPs comprise 23 different members in human and mouse (MMP-1 to MMP-28) that are not numbered consecutively. These enzymes act as zinc-dependent endopeptidases that have a role in a diverse range of physiological and pathological processes (Visse and Nagase, 2003; Jackson et al., 2010). Under normal conditions, the different MMPs degrade and remodel the ECM with different substrate specificities. They are synthesized as inactive zymogens and become activated by intracellular, extracellular, or cell surface-mediated proteolysis. Once activated, the extracellular activity of MMPs is controlled by TIMPs. These form a group of four members (TIMP-1, TIMP-2, TIMP-3, and TIMP-4) with different affinity for individual MMPs. The balance of MMP synthesis, activation and inhibition by TIMPs is most critical for ECM homeostasis and overactivity of TIMPs or suppressed expression of MMPs favors fibrosis progression. In humans, there are only a few MMPs (MMP-1, MMP-2, MMP-3, MMP-8, MMP-9, MMP-13, MMP-14) and TIMPs (TIMP-1, TIMP-2) expressed in liver tissue or relevant for hepatic fibrogenesis and fibrolysis. A detailed literature search that analyzed 243 Medline records describing experimental models of liver fibrosis that evaluated a defined anti-fibrotic intervention showed that all these genes have a characteristic expression during degradation of normal liver matrix, accumulation of fibrotic tissue and degradation of fibrotic liver matrix (Hemmann et al., 2007). While for example MMP-2 and MMP-14 are relevant during phases of accumulation of fibrotic scar tissue, the activity of MMP-13 is particular observed during degradation of normal tissue and in the initiation process of degradation of fibrotic scar tissue suggesting that the individual MMPs have pro- and anti-fibrotic activities (Hemmann et al., 2007).

In line with this concept, the small interfering RNA targeting TIMP-1 expression effectively suppressed MMP-2 expression and activity in the CCl_4_ and BDL models in rats, while the expression and activity of MMP-13 was elevated (Cong et al., 2013). Likewise, the direct targeting of TIMP-2 by a synthetic siRNA resulted in promotion of ECM degradation, decreased MMP-2 expression, and increased MMP-13 expression (Hu et al., 2007). Other therapy strategies were based on the assignment of inactivated MMP enzymes acting as profound scavengers for TIMP-1. Mice that were treated with proteolytically inactive MMP-9 mutants showed overall lower fibrosis scores and were effective in preventing transdifferentiation of HSC *in vitro* and *in vivo* (Roderfeld et al., 2006).

Moreover, different cytokines are effective in modulating the activity of the MMP/TIMP axis. Members of the PDGF family (i.e., PDGF-B, PDGF-D) induce expression of TIMP-1 thereby attenuating MMP-2 and MMP-9 activities in HSC and portal MFB *in vitro* (Borkham-Kamphorst et al., 2015a,b). Likewise, there are many other compounds or mixtures of substances extracted from plants or fungi that directly or indirectly influence expression or activity of different MMPs or their inhibitors. A polysaccharide extracted from the entomopathogenic fungi *Cordyceps sinensis mycelia* reduced activities of MMP-2 and MMP-9 (Peng et al., 2013a), demonstrating that the modalities to interfere with MMP activity are manifold.

### Integrins, RGD peptides, and RGD analogs

Integrins are divalent cation-dependent heterodimeric, membrane glycoproteins that are composed of non-covalently associated α- and β-subunits. They regulate cell-cell contacts and cellular interactions and attachment of immune cells with the ECM. Most integrins recognize their respective ECM proteins through short peptide stretches such as Arg-Gly-Asp (RGD), Glu-Ile-Leu-Asp-Val (EILDV), or Arg-Glu-Asp-Val (REDV; Supplementary Figure 6). For therapy of hepatic fibrosis, the RGD peptide has attracted much interest because this peptide is the predominant cell adhesive epitope to fibronectin and well known to bind preferentially and with high affinity to the integrin α_v_β_3._This integrin and other integrins play essential role in angiogenesis and in fibroblasts where they are central in differentiation, migration, and proliferation. They have many ligands (e.g., vitronectin, fibronectin, fibrinogen, osteopontin, and CCN1/CYR61) that are important in initiation or progression of hepatic fibrosis. Although integrins have no intrinsic enzymatic activity, they are capable to initiate diverse signaling cascades after binding to its ECM partner. They not only signal on their own but can also cooperate with growth factor receptors or modify the activity of enzymes or cytokines that are involved in ECM homeostasis. As good ligands, the short RGD peptides are already able to induce a conformational change from a closed to an open integrin conformation (Hantgan et al., 1999). Therefore, these soluble peptides can compete with insoluble ECM proteins for binding to integrin receptors thereby preventing the initiation of signal cascades or preventing cell adhesion. However, the RGD peptide itself is not stable and rapidly degraded. Therefore, several non-peptidic RGD mimetics such as the SF-6,5 that contains guanidinium and carboxylic groups separated by an 11-carbon atom backbone that mimic the distal configuration of functional RGD sequence were developed (Greenspoon et al., 1993). This compound is proteolytically more stable and serves as a useful therapeutic agent in versatile pathological processes. In 1996, it was shown that this RGD analog is therapeutically useful and suitable to inhibit progression of TAA-induced fibrosis in rats (Bruck et al., 1996). Similar findings were reported for the RGD peptide GRGDS in the CCl_4_ model when injected intraperitoneally three times a week for 1 month but were not noticed when the control peptide GRGES were applied (Kotoh et al., 2004). *In vitro*, the RGD peptide reduced accumulation of type I collagen and increased the secretion of collagenases by HSC (Iwamoto et al., 1999). A cyclic peptide (C^*^GRGDSPC^*^) that has high affinity to the collagen type VI receptor binds preferentially to activated HSC and when coupled to sterically stable liposomes these particles accumulated *in vivo* 10-fold higher to HSC than uncoupled liposomes in BDL rats (Du et al., 2007). The concept that the RGD sequence is suitable to target drugs specifically to activated HSC was also exploited in another approach in which the quinolizidine alkaloid oxymatrine from the root of the evergreen slow growing shrub (*Sophora flavescens*) was specifically targeted by RGD peptide-labeled liposomes to HSC in the CCl_4_ model in rats (Chai et al., 2012). Also the encapsulation of oxymatrine into RGD-labeled biodegradable polymersomes ignorantly reduced the expression of α-SMA and collagen Iα1 *in vitro* and decreased hepatic connective tissue deposition in BDL rats (Yang et al., 2014b).

### Malotilate

This sulfur-containing drug (diisopropyl 1,3-dithiol-2-ylidenemalonate; Supplementary Figure 7) was shown to prevent paracetamol-induced liver damage in male mice when given 1 h before application of paracetamol, most likely by interacting with the bioactivation of paracetamol (Younes and Siegers, 1985). Likewise, malotilate drastically reduced hepatic inflammation, the increases in type I procollagen α2 mRNA and activities of prolyl 4-hydroxylase and galactosylhydroxylysyl glucosyltransferase that are key mediators in collagen fibril formation in dimethylnitrosamine-treated rats (Ala-Kokko et al., 1989). Malotilate was well tolerated in phase II study that enrolled a small number of patients with cirrhosis of various aetiologies and severity (Bührer et al., 1986) and shown to accelerate the recovery of impaired protein metabolism in alcoholic liver disease (Takase et al., 1989). However, this drug is nowadays out of the focus, potentially because it is poorly water-soluble and has low oral bioavailability. It will be interesting to follow if novel spray-dried malotilate emulsions that can produce higher plasma concentrations (Zhang et al., 2011a) will lead to a revival in hepatic malotilate research.

### Tranilast

This drug (Supplementary Figure 7) was originally approved in Japan and South Korea and brought into the market in 1982 as an anti-allergic drug (Rizaben). Some years later, an inhibitory effect on fibroblast proliferation and collagen synthesis was found both *in vitro* and *in vivo* (Isaji et al., 1987; Yamada et al., 1994). Thereafter, this drug was successfully tested in different experimental models of liver injury. Tranilast dose-dependently reduced mRNA expression of procollagen and TGF-β1 in cultured rat HSC (Ikeda et al., 1996). *In vivo* it was recently shown that tranilast has anti-inflammatory potential, antioxidant activity, and immunomodulatory properties in TAA-induced acute liver injury (Abdelaziz et al., 2015). However, human trials with this drug were not initiated yet.

### β-aminopropionitrile

This small substance (BAPN) consists of an amine and nitrile group that are linked *via* a C2 linker (Supplementary Figure 7). It occurs in sweet pease and other Lathyrus plants and irreversibly inhibits the enzymatic activity of the Lysyl oxidase. This enzyme is copper-dependent and cross-links collagen or elastin by oxidative deamination of peptidyl lysine or hydroxylysine and peptidyl lysine residues (Bondareva et al., 2009). In a model in which rats were treated with CCl_4_ twice weekly for 3–70 days, BAPN prevented early increase in liver stiffness suggesting that it effectively prevented HSC activation, collagen expression, crosslinking or deposition (Georges et al., 2007). Although these findings are encouraging, more studies are still required before clinical trials are performed in humans.

### S 4682, S 0885, HOE 077, safironil

S 4682 is a heterocyclic carbonyl-glycine inhibitor of the prolyl 4-hydroxylase that catalyzes the crucial step in intracellular collagen processing. This drug reduced hepatic collagen accumulation in the CCl_4_ model in rats (Bickel et al., 1998). A similar effect was found with S 0885 (Supplementary Figure 7) that is structurally related to S 4682 (Bickel et al., 1991). The competitive prolyl 4-hydroxylase inhibitors HOE 077 is a hydrophilic prodrug that can be converted to active hydrophilic metabolites only within hepatocytes. Like its congener safironil, HOE 077 prevented stellate cell activation, expression of TIMP-1 mRNA, and formation of collagen deposits in rat liver fibrosis (Bickel et al., 1991; Wang et al., 1998; Sakaida et al., 1999).

### Lysyl oxidase-like-2

The enzyme LOXL2 catalyzes a rate limiting step that is necessary in scaffolding and crosslinking of collagens and elastins. Moreover, this enzyme is involved in the formation of a microenvironment that is crucial for scar tissue formation in hepatic fibrosis (Barry-Hamilton et al., 2010). Targeting LOXL2 with an inhibitory monoclonal antibody (AB0023) was highly effective in targeting liver and lung fibrosis (Barry-Hamilton et al., 2010). Studies with a humanized monoclonal antibody against LOXL2, termed Simtuzumab (GS-6624), were recently initiated and are currently being investigated in diverse ongoing phase 2 trials involving HCV patients, primary sclerosing cholangitis, NASH-related fibrosis and cirrhosis. Since the expression of LOXL2 is also influenced by hypoxia, TGF-β, and microRNAs (miR-26 and mIR-29), there are also other potential strategies for targeting LOXL2 expression or activity (Wong et al., 2014).

## Histone deacetylase and acetyltransferase blockers

Histone deacetylases (HDAC) are a family of enzymes that remove acetyl groups from histones thereby suppressing general gene transcription. Their activities are counterbalanced by histone acetyl transferases (HAT) that acetylate lysine amino acids on histones resulting in relaxed chromatin structures. The ratio of acetylation and de-acylation adjudicate gene regulation. Substances that prevent deacetylase or transferase activity are suitable to modulate gene expression, replication, mitosis, meiosis, and apoptosis. In addition, there are some non-histone protein substrates of histone deacylases that are involved in control of cell proliferation, cell migration, and cell death.

The 18 mammalian HDACs are grouped into four major classes that are distinguished by their inhibitability by different substances. They counteract oxidative stress and inflammation and further influence MAP kinase driven pathways (Ferguson and McKinsey, 2015). In experimental hepatology research, several HDAC inhibitors have become in focus because they can induce growth arrest, terminal differentiation, intrinsic and extrinsic apoptotic pathways, autophagic cell death, and senescence already at low concentrations (Dokmanovic et al., 2007).

### Curcumin

This natural phenol with a diarylhepanoid structure is the yellow pigment associated with the curry spice that can be isolated from *Curcuma longa* (turmeric) and to a lesser content from ginger (Supplementary Figure 8). It is an inhibitor of p300 histone deacetylases, cyclooxygenase, and arachidonate 5-lipoxygenase and is further a compound that modulates NF-κB, MAPKs, and pathways involved in apoptosis (Liu et al., 2005a). There are a large wealth of experimental studies that have unanimously shown that cucuminoids evolve strong antioxidant, anti-inflammatory, and anti-fibrotic properties Chainani-Wu, 2003). It was shown that the dietary administration of whole spice turmeric or ethanolic extracts thereof predict changes in liver parenchymal cells in mice (Kandarkar et al., 1998). At the molecular level, it was recently demonstrated that curcumin modulates cell fate and metabolism by abrogating Hedgehog signaling by downregulating essential key elements of this pathway (i.e., Patched and Smoothened) in HSC (Lian et al., 2015). Curcumin has also some other potential target sites in HSC rendering that principal curcuminoid of turmeric as a potential candidate to prevent or treat hepatic fibrosis in humans (Tang, 2015).

### Trichostatin A

This antifungal and antibiotic drug (Supplementary Figure 8) inhibits HDAC and cell cycle by interfering with the removal of acetyl groups form histones and induction of apoptosis-related genes. Work performed in telomerase-immortalized human corneal fibroblasts has shown that this drug has antioxidant activity preventing TGF-β-induced formation of ROS and H_2_0_2_ and MFB differentiation by triggering the Nrf2-ARE signaling pathway (Yang et al., 2013). In primary rat HSC, trichostatin A induced hyperacetylation of histone H4 affecting proliferation, transdifferentiation, and expression of fibrosis-associated genes (Niki et al., 1999). Moreover, this compound inhibited the occurrence of TGF-β1-induced epithelial-to-mesenchymal transition in hepatocytes (Kaimori et al., 2010). In the field of experimental hepatology, the therapeutic efficacy of that drug on hepatic fibrosis was not tested in animals yet.

### Valproic acid

This substance (Supplementary Figure 8) is in use as an anticonvulsant since several decades. Although the molecular mechanism of its activity is not fully understood, it is believed that this drug interferes with the gamma-aminobutyric acid (GABA) metabolism and acts as a potent HDAC inhibitor. Although the treatment with valproic acid in long-term induced impairment of mitochondrial function and strong hepatotoxicity in humans (Pessayre et al., 1999), the chronic administration of valproic acid inhibited HSC activation and hepatic fibrogenesis in mice (Mannaerts et al., 2010). Beneficial effects of valproic acid were also found in rats with TAA-induced hepatic fibrosis in which the drug prevented the activation of HSC and decreased collagen deposition, infiltration of inflammatory cells, and further induced DNA damage and apoptosis in activated HSC (Aher et al., 2015). Therefore, the view that the administration of this drug for hepatic fibrosis still needs careful evaluation is still valid (Ikura et al., 2010).

### Epigallocatechin-3-gallate

This gallic acid (EGCG) has a catechin structure that is one of the major effective ingredients of green tea. It has antioxidant activity and inhibits HATs, DNA methyltransferases, and tyrosinases. EGCG is further effective in preventing NF-κB and TNF-α activation (Choi et al., 2009) and induction of cell cycle arrest and apoptosis (Du et al., 2012). EGCG was shown to prevent hepatic inflammation, oxidative stress formation and ongoing hepatic fibrosis in mice that were subjected to CCl_4_ (Tipoe et al., 2010). Similar results were found in mice in which liver injury was induced by BDL (Shen et al., 2015), and further in MCD diet-induced hepatic steatosis in which EGCG inhibited IL-1β, IL-6, TNF-α, and MCP-1 mRNA expression (Ding et al., 2015). Furthermore, ECGC suppressed the phosphorylation of Smad2/3 and Akt/PKB and produced similar effects as LY294002 (that is a highly selective inhibitor of phosphatidylinositol 3 kinase), possibly suggesting that some of its activities are due to inhibition of the PI3K/Akt/Smad pathway (Yu et al., 2015).

## Angiotensin receptor antagonism

The peptide hormone angiotensin is part of the renin-angiotensin system and acts through a class of G protein-coupled receptors that in their activated forms activate phospholipase C, cellular calcium increase, and protein kinase C. Based on their binding specificities for ligands, the receptors can be classified into four subtypes, i.e., AT_1_R, AT_2_R, AT_3_R, and AT_4_R (Unger et al., 1996). The activity of angiotensin can be either blocked by angiotensin-converting enzyme (ACE) inhibitors that block the conversion of the decapeptide angiotensin I to the biological active octapeptide angiotensin II, or alternatively by antagonistic angiotensin II receptor blockers. Some ACE inhibitors (e.g., perindopril) and several selective AT_1_ receptor blockers (losartan, candesartan) have attracted the interest of hepatic fibrosis research (Supplementary Figure 9). Historically, these drugs become into the focus by the finding that perindopril and candesartan were both effective in preventing pig serum-induced liver fibrosis in rats (Yoshiji et al., 2001). Subsequently, Bataller and co-workers have shown that the infusion of angiotensin II during ongoing BDL-induced hepatic fibrosis in rats significantly augmented hepatic fibrosis and promoted inflammation, oxidative stress formation, and thrombogenic events (Bataller et al., 2005). Countless subsequent proof-of-concept studies confirmed that the renin-angiotensin system significantly contributes to the pathogenesis of hepatic fibrosis (Munshi et al., 2011). Recently, also some direct renin inhibitors (e.g., aliskiren) that inhibit the formation of angiotensin I from angiotensinogen were successfully tested in rodent models of hepatic injury (Lee et al., 2013) suggesting that the renin-angiotensin system offer many potential therapeutically beneficial drug targets for interfering with hepatic fibrosis.

## Endothelin receptor antagonism

Endothelins are a group of three (ET-1, ET-2, ET-3) potent vasoconstrictive peptides that can bind with different affinity to two G-protein coupled receptors, i.e., ET_A_ and ET_B_ (Maguire and Davenport, 2015). These receptors are detectable on all major hepatic cell types but are most abundant expressed on HSC in which the expression of ET-1 is significantly activated during cellular activation (Housset et al., 1993). ET-1 (Supplementary Figure 10) and the 21-residue peptide Sarafotoxin (S6C) that represent a potent ETB receptor agonist are highly effective in stimulating HSC activation, while the mixed ET_A_-ET_B_ receptor antagonist bosentan blocked this activity (Rockey and Chung, 1996). However, bosentan that was applied in clinical studies for treatment of pulmonary hypertension, turned out to evolve hepatotoxic effects hampering the initiation of human trials testing the efficacy of ET blockers in patients suffering from liver disease (Humbert et al., 2007). Although the endothelin axis shifted out of the attention during the last years, the complex physiology of the ET-1 system offers many potential drug target that are particular suitable to interfere with initiation of the fibrotic response (Rodríguez-Pascual et al., 2014).

## Leptin antagonism

Leptin is an adipokine that acts as a hormone and is majorly involved in the regulation of energy balance. It binds to a cerebral receptor that regulates appetite and body weight. Although predominantly synthesized in adipose tissue, leptin is also secreted from activated HSC (Ding et al., 2005). In addition, leptin can induce expression of TGF-β and CTGF expression in Kupffer cells thereby promoting the progression of hepatic disease (Wang et al., 2009). In line with this assumption, the application of leptin was associated with significant enhanced TAA-induced liver disease, while the application of a competitive leptin inhibitor attenuated chemically induced hepatic fibrosis in mice and suppressed profibrogenic potential of HSC (Elinav et al., 2009). Therefore, both the application of leptin inhibitors and the application of adiponectin that opposes leptin functions and signaling were proposed as effective means to interfere with hepatic fibrogenesis (Handy et al., 2011).

## Thrombin antagonism and anticoagulants

The serine protease thrombin is an essential converting factor that is required in the coagulation cascade, and implicated in the physiology of blood clot formation. Beside this direct activity of thrombin in control of haemostasis, thrombin can activate four different protease-activated receptors (PAR-1, PAR-2, PAR-3, and PAR-4) that belong to the seven transmembrane G-protein-coupled receptor family. These stimulate upon proteolytic activation cell proliferation and transdifferentiation in HSC, while their expression increases in parallel with the severity and duration of liver disease (Calvaruso et al., 2008). In experimental fibrosis in rats induced by administration of CCl_4_, the synthetic thrombin inhibitor SSR 182289 was found to decrease the content of α-SMA and expression of TIMP-1 (Duplantier et al., 2004). Exposure of HSC to a PAR-1 antagonists resulted in inhibition of primary rat HSC activation *in vitro* and protected against fibrosis development in a rat model of cirrhosis induced by BDL (Fiorucci et al., 2004). Conversely, PAR-1 and PAR-2 agonists induced stellate cell proliferation, again demonstrating the importance of thrombin in the formation of liver fibrosis (Gaça et al., 2002). Also the application of substances that prevent the proteolytic activation of PAR receptors have anti-fibrotic capacity. Such a drug is APC 366 that selectively blocks the mast cell tryptase prevented hepatic fibrosis in rats (Lu et al., 2014). Strategies counteracting PAR activity are therefore still one attractive option for promotion of restoration of liver health. Likewise, other compounds with anticoagulant activity, such as warfarin (coumadin), might be successful to modulate pre-existing fibrosis. However, evaluation of their anti-fibrotic potential is still pending.

## Tyrosine kinase inhibitors

Tyrosine kinases are enzymes that catalyze the transfer of the phosphate groups from nucleotide triphosphates (e.g., ATP, GTP) to tyrosine residues in a protein substrate. This phosphorylation causes structural changes that induce altered functions or enzymatic activities. Tyrosine kinases can be classified into two groups, i.e., the transmembrane receptors and the cytoplasmic non-receptor tyrosine kinases. Both groups are linked to fundamental functions such as cell differentiation, proliferation, growth factor signaling, apoptosis, and malignant transformation. During the last decades, the understanding in the biochemical activity of receptor tyrosine kinases (RTKs) increased dramatically. Simultaneously, the number of RTK inhibitor substances (Supplementary Figure 11) that pharmaceutical companies developed for disease therapy increased dramatically (Hunter, 2009).

Fibrotic disorders in many organs are influenced by RTKs and non-receptor tyrosine kinases. Most prominently in fibrogenesis are the transmembrane receptors for PDGF, TGF-β, VEGF, EGF, FGFR, and JAK and the non-receptor tyrosine kinase c-*Abl* and c-*src* (Beyer and Distler, 2013). Nowadays, highly selective or pan-specific inhibitors targeting activity of multiple RTKs are on the market. In hepatology research, many of them were tested experimentally in ongoing and established fibrosis. Imatinib (STI-571) that is specific for Abl and PDGF receptors attenuated liver fibrosis and increased apoptosis, suppressed expression of α-SMA and type I collagen, and further reduced proliferation (Kuo et al., 2012). The same beneficial effects were reported for the structurally related inhibitor nilotinib (AMN107) that showed anti-fibrotic effects in CCl_4_- or to TAA-treated rats (Shaker et al., 2011a,b; Shiha et al., 2014). The oral, multi-targeted RTK inhibitor sunitinib (Su11248) blocked collagen synthesis, attenuated contraction, reduced cell migration without affecting cell viability, and inhibited PDGF-induced activation of ERK1/2 MAPK and AKT/PKB phosphorylation in LX-2 cells (Tugues et al., 2007; Majumder et al., 2013). Beneficial effects of sunitinib were also shown in rats that were treated with CCl_4_ (Tugues et al., 2007). The multikinase blocker sorafenib attenuated liver injury and profibrogenic signaling in mice and rats in a large variety of experimental models (Wang et al., 2010; Hennenberg et al., 2011, p. 253; Thabut et al., 2011; Deng et al., 2013; Hong et al., 2013; Su et al., 2015). Orantinib (TSU-68) that has greatest potency against PDGFR auto-phosphorylation was recently shown to block growth in HCC and fibroblast cell lines (Hara et al., 2015). Worldwide, numerous other multi-targeted RTK inhibitors (e.g., pazopanib) are currently tested in experimental models.

## Metals

Metals play pivotal roles in the liver. They are essential for proper protein function, structure and stability. As part of enzymes, they act as cofactors, or are indispensable direct catalysts in biochemical reactions (Susnea and Weiskirchen, 2015). Consequently, the lack of an individual trace element leads to shortcomings, failures, and illness. On the contrary, excess metal supply resulting from genetic disorders, intoxication, exorbitant dietary intake, organ failure, or during therapeutic treatments such as blood transfusion and long-term parenteral nutrition may result in elevated metal concentrations and deposits. Mechanistically, hepatic overload with metals (i.e., iron, copper) results in elevated ROS formation triggering cell stress, apoptosis, and necrosis in hepatocytes. Therefore, cells have developed sequestering mechanisms that protect against metal toxicity. Best known is the family of metallothioneins that bind a multitude of metals through thiol groups.

On the other side, some metals (particularly zinc) partly functions as an antioxidant and zinc supplementation is associated with decreased ROS formation (Kloubert and Rink, 2015). In BDL mice, zinc supplementation was effective in suppressing progression of fibrosis through inhibition of collagen production and by enhancing collagen degradation (Shi et al., 2015). Mechanistically, inadequate supply with zinc results in HSC activation that is associated with a reduction in intracellular glutathione and increased quantities of intracellular H_2_O_2_ (Kojima-Yuasa et al., 2005). Although clinical trials in liver-diseased patients are limited in size and quality, first results from a double-blind, placebo-controlled human trial with a small number of patients showed that zinc supplementation for 3 months significantly reduced blood ammonia levels in patients with liver cirrhosis and hyperammonemia (Katayama et al., 2014).

## Herbal supplements

There is a bulk of reports describing the use of crude, unclassified herbal preparations, purified mixtures, or single plant compounds for treatment of experimental hepatic disease. Most of these medicinal formulations and remedies have their origin in Traditional Chinese Medicine (TCM) that use ingredients form plants, animals, humans, and mineral products for healing. Although most the knowledge of TCM drugs was developed over generations, it is thought that they have no real rational mechanism of action (Nature, 2007). However, there is no doubt that some of these “indigenous” drugs have produced wonderful miracles in experimental research. Therefore, several of these drugs became in the focus of global interest and natural ingredients served as blueprints for the synthesis of novel therapeutic drugs. Actually, there is a rapid growth in the field of “Pharmacognosy” in which scientists bundle their knowledge of bioactive products derived from natural sources. Particularly, countless studies from Asia report the successful usage of TCM (“folk medicines”) in animal studies. Also in Western countries, TCM drugs are becoming increasingly important. An interdisciplinary network in Germany, for example, has set up a program in 1999 in which methods for sustainable field production and post-harvest processing are developed for selected plant species. The major the aim of this initiative is to improve herbal drug quality and safety and to make these drugs more reliable for all users (Heuberger et al., 2010).

In the following, some herbs or mixtures including their most important compounds are discussed.

### Sho-saiko-to

**Figure 9:**
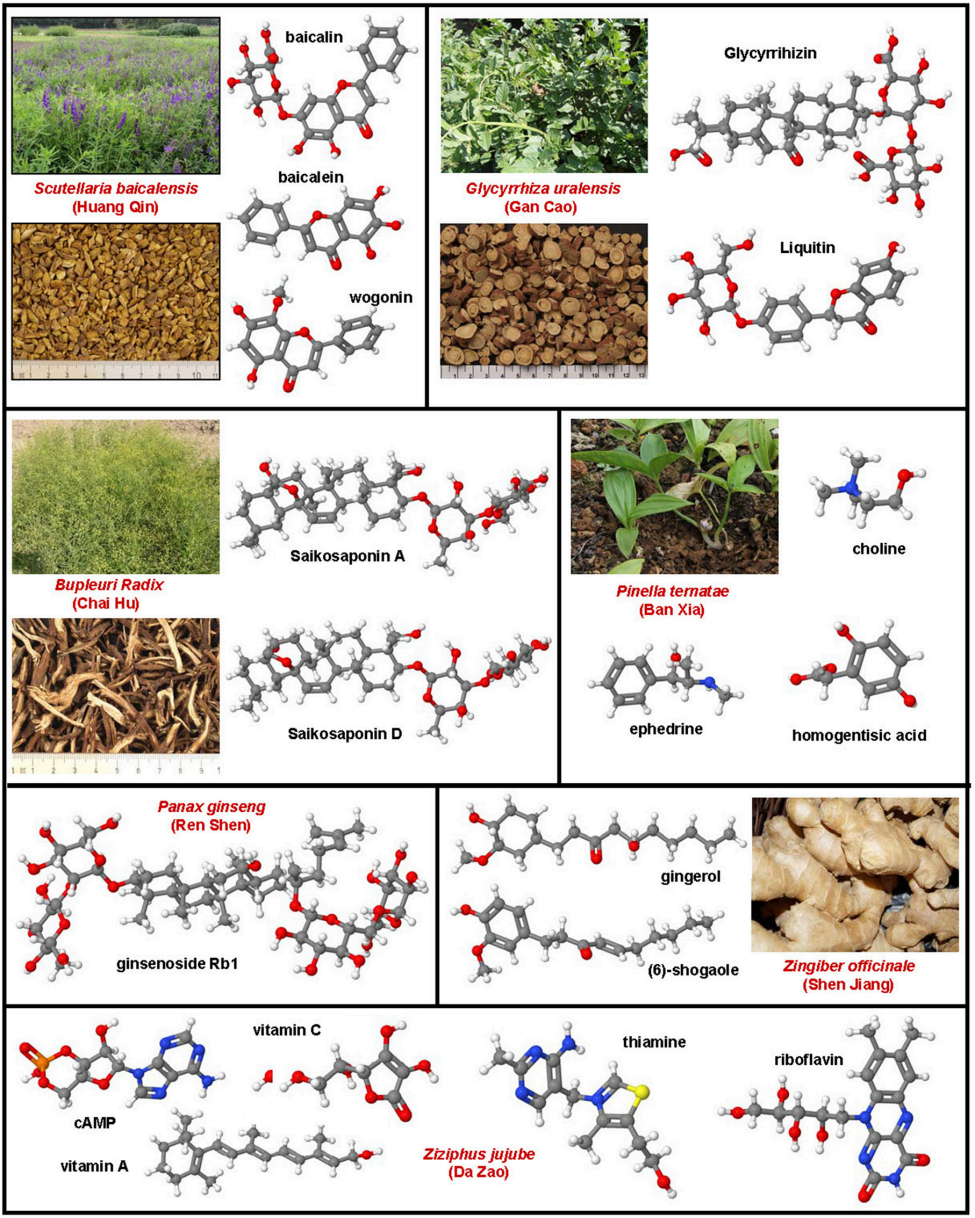
**Sho-shaiko-to a herbal mixture and its main ingredients**. Sho-saiko-to contains root of *Bupleurum*, root/stolon of *Glycyrrhiza*, root w/o rootlets of *Panax ginseng*, tuber w/o cork layer of *Pinelliae*, rot w/o periderm of *Scutellaria*, root/rhizome of *Zingiber*, and fruit of *Ziziphus jujube.* Each herb contribute some key ingredients that together are made responsible for the antifibrotic activity of Sho-saiko-to. Bupleurum contains different Saikosaponins (e.g., Saikosaponin A, CAS 20736-09-8; Saikosaponin D, CAS 20874-52-6), while licorice is enriched with the glycyrrihizin (CAS 1405-86-3) and liquiritin (CAS 551-15-5). Ginseng contains different ginsenosides and panaxic acid. Pinellia is enriched with homogentisic acid (CAS 451-13-8), ephedrine (CAS 299-42-3), and choline (CAS 67-48-1). Baical skullcap contains baicalin (CAS 21967-41-9), baicalein (CAS 491-67-8), and wogonin (CAS 632-85-9). The active components of ginger are gingerol and shogoal. Jujube is enriched in cyclic AMP (CAS 60-92-4), vitamin C (CAS 50-81-7), vitamin A (CAS 68-26-8), niacin (CAS 59-67-6), riboflavin (*CAS* 83-88-5), and thiamine (CAS 59-43-8). The plant images were provided by Dr. Heidi Heuberger (LfL, Bayerische Landesanstalt für Landwirtschaft, Freising, Germany, www.lfl.bayern.de/).

The mixture that is also known as TJ-7 or Xiao Chaihu Tang is composed of seven herbals (Figure 9). It contains 17.5% root of *Bupleurum falcatum* (Bupleurum, Chai Hu, Saiko), 5.0% root/stolon of *Glycyrrhiza uralensis* (Licorice, Gan Cao, Kanzou), 7.5% root w/o rootlets of *Panax ginseng* (Asian ginseng, Ren Shen, Ninjin), 12.5% tuber w/o cork layer of *Pinelliae ternatae* (Pinellia, Ban Xia, Hange), 7.5% rot w/o periderm of *Scutellaria baicalensis* (Baical skullcap, Huang Qin, Ougan), 2.5% root/rhizome of *Zingiber officinale* (Ginger, Sheng Jiang, Shokyo), 7.5% fruit of *Ziziphus jujube* (Jujube, Da Zao, Daiso), and 40% additives (Nishimura et al., 2001; Wen, 2007). Each of these herbs contains several active biochemical constituents that are considered to predict their pharmacological effects. Bupleurum contains different Saikosaponins (e.g., A, B, C, D) that represent a group of oleanane derivatives predicting an anti-inflammatory action. Licorice is enriched with the saponin Glycyrrihizin and the flavanone Liquiritin that are both supposed to have anti-inflammatory and mineralocorticoid activity. Ginseng contains ginsenosides (steroid glycosides) and panaxic acid that strengthen the immune system and act as partial agonists of steroid hormone receptors. Pinellia is enriched with homogentisic acid, the sympathomimetic amine ephedrine, and choline that is an essential compound necessary for structural integrity and signaling of cell membranes. The anxiolytic and sedative flavone baicalin, its aglycone baicalein, and wogonin that have anti-oxidative, anti-inflammatory, and anti-viral activity are considered to be therapeutic effective drugs of the Baical skullcap. The relative of capsaicin gingerol and its dehydrated product shogoal are the active compounds present in high concentration in the rhizomes of ginger. Both compounds exhibit a multitude of biological activities, ranging from anti-cancer, anti-oxidant, anti-microbial, anti-inflammatory, and anti-allergic to various central nervous system activities (Semwal et al., 2015). The fruits of jujube are enriched in cyclic AMP, vitamins (vitamin C, vitamin A, niacin, riboflavin, and thiamine) and different glycosides that may have significant pharmacological actions. Although it is hard to estimate and verify how the individual ingredients exhibit their anti-fibrotic effects, studies in rats that were subjected to a choline-deficient L-amino acid-defined diet-induced liver fibrosis showed that the dietary intake of 1% (w/w) Sho-saiko-to prevents fibrosis by inhibiting the activation and proliferation of HSC (Sakaida et al., 1998). Another beneficial effect of Sho-saiko-to is its potent anti-fibrosuppressant activity that is *in vivo* mediated by its inhibitory effect on lipid peroxidation both in hepatocytes and HSC (Shimizu et al., 1999). In line, these beneficial effects on hepatic fibrosis were also achieved by other herbal mixtures (TJ-135, Inchin-ko-to; TJ-48, Juzen-taiho-to; TJ-25, Keishi-bukuryo-gan) in the db/db mouse model of NASH (Takahashi et al., 2014).

### Salvia miltiorrhiza

**Figure 10:**
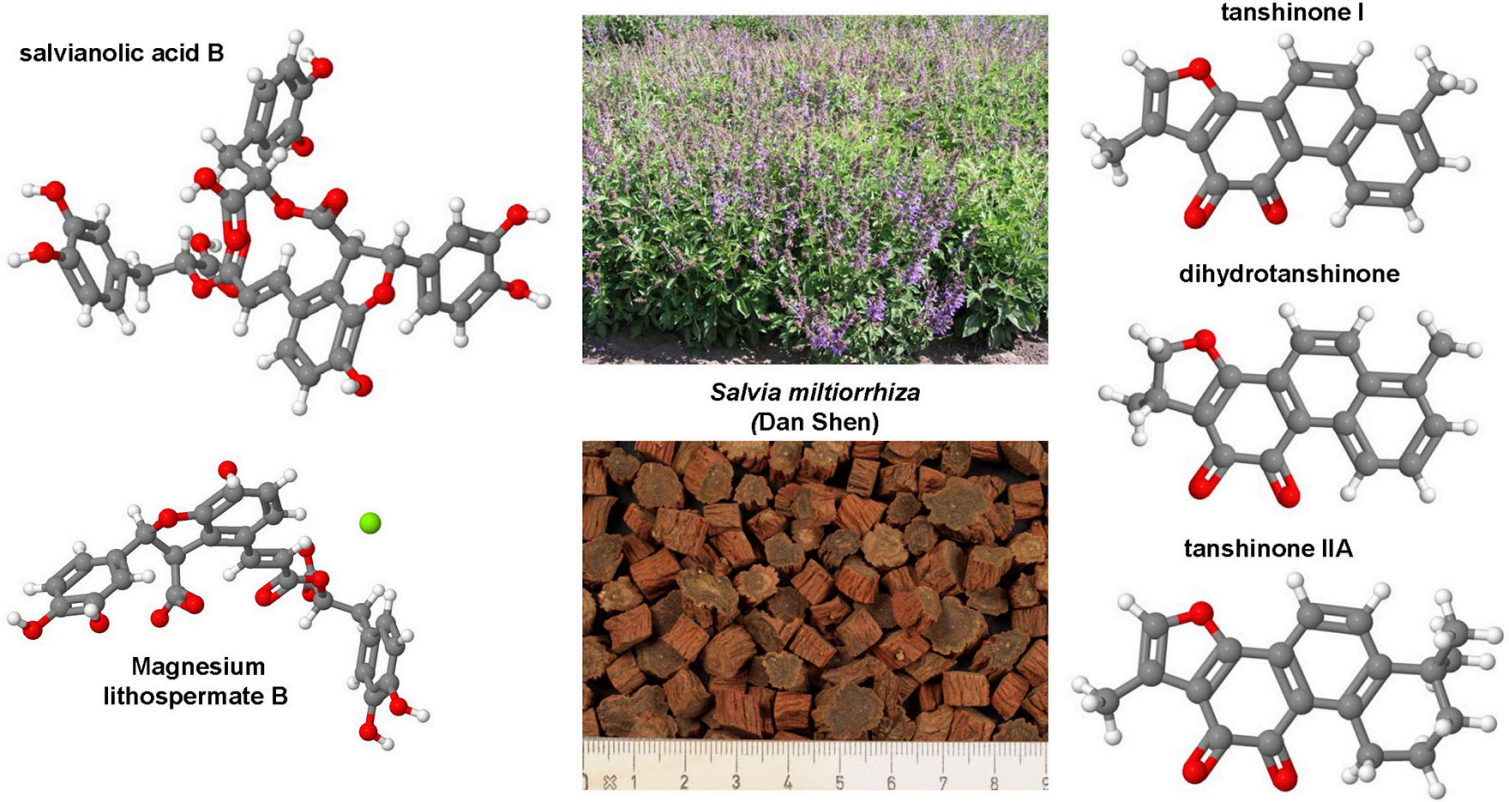
**Salvia miltiorrhiza and its active compounds**. In the roots of *saliva miltiorrhiza* are many ingredients with antifibrotic activity including salvianolic acid B (CAS 121521-90-2), magnesium lithospermate B (CAS 122021-74-3), tanshinone I (CAS 568-73-0), dihydrotanshinone (CAS 20958-18-3), and tanshinone IIA (CAS 568-72-9). The plant images of *Salvia miltiorrhiza* were provided by Dr. Heidi Heuberger (LfL, Bayerische Landesanstalt für Landwirtschaft, Freising, Germany).

This plant is also known as red sage and the dried roots are known in TCM as Dan Shen. This herb contains a great wealth of flavonoids, terpens, chinons, lignans, steroids, and tannins (Figure 10). However, in regard to their beneficial effects on liver fibrogenesis, specific ingredients with antioxidant properties (e.g., salvianolic acid B, tanshinone IIA), cytotoxic attributes (dihydrotanshinone), or substances that interfere with cell migration, cell invasion and MMP activity (tanshinone I) are presently discussed as the major bioactive compounds in *Salvia miltiorrhiza*. In addition, magnesium lithospermate B and the monomer IH764-3 are further in the focus of experimental studies. When Salvia was given from the onset of CCl_4_ treatment it effectively reduced expression of TGF-β, collagen types I and III, TIMP-1, and further increased expression of MMP-13 in rats (Wasser et al., 1998). Likewise, water-soluble extracts of *Salvia miltiorrhiza* suppressed decreased activity of caspase-1 and expression of Bax, Bcl-2, and cytochrome c thereby preventing hepatic apoptosis in rats (Lee et al., 2006). Similar results were reported for a standardized fraction of *Salvia miltiorrhiza* (PF2401-SF) in rats in which hepatic fibrosis was induced by TAA (Parajuli et al., 2015). Also the *in vitro* or *in vivo* beneficial effects of individual *salvia miltiorrhiza* ingredients such as salvianolic acid B (Wang et al., 2012), salvianolic acid A (Lin et al., 2006), tanshinone IIA (Pan and Wang, 2012), dihydrotanshinone I (Liu et al., 2010), tanshinone I (Kim et al., 2003), magnesium lithospermate B (Paik et al., 2011), and monomer IH764-3 (Liu et al., 2012) was proven experimentally. The combined administration of lithospermate B and the caspase-inhibitor nivocasan effectively reversed hepatic fibrosis in rats demonstrating that the addition of other pharmacological active constituents might enhance the beneficial effects of this herbal drug (Kim do et al., 2013).

### Astragaloside IV

**Figure 11:**
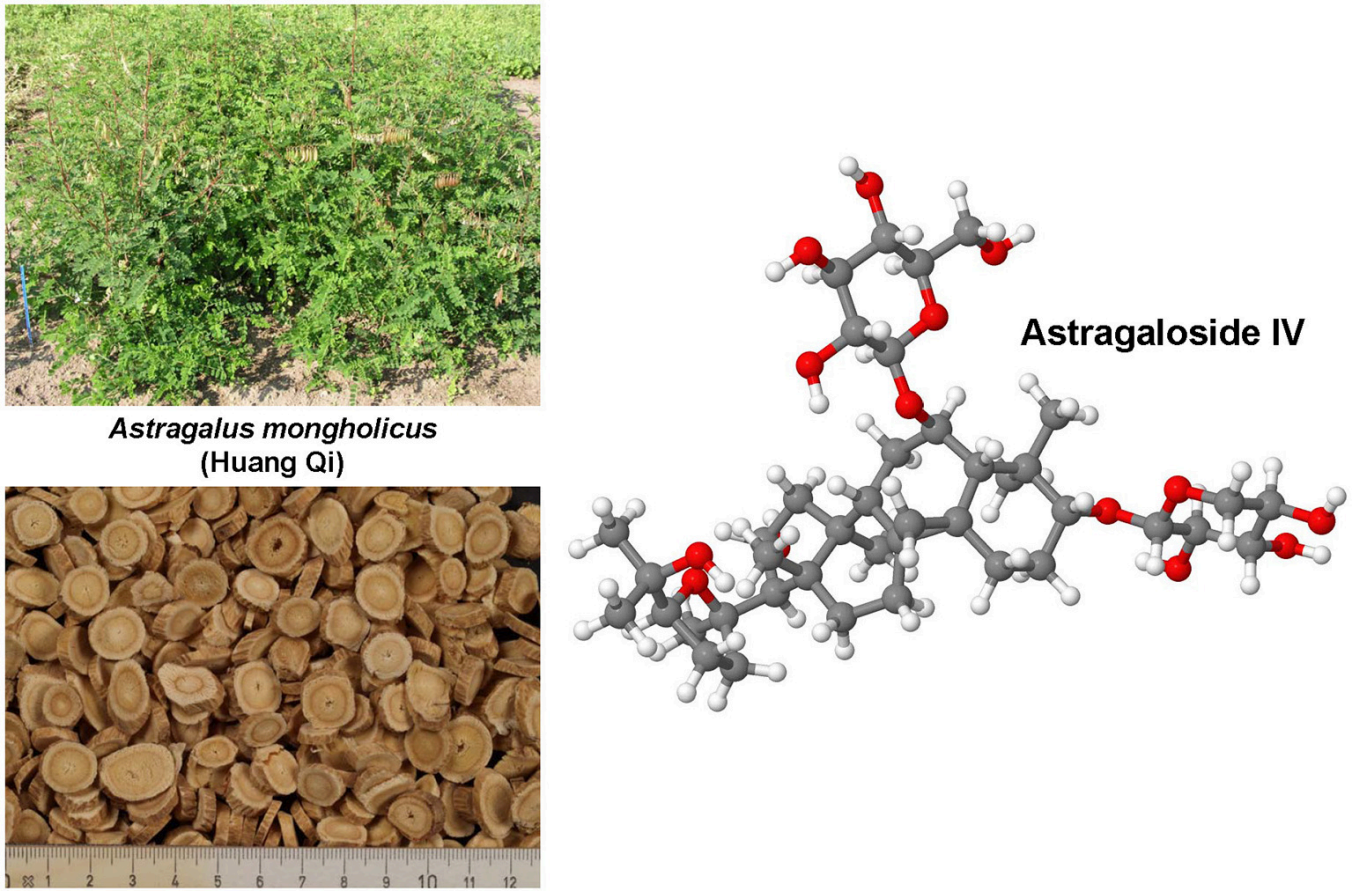
**Astragaloside IV**. This compound (CAS 84687-43-4) is a lanolin alcohol-shaped tetracyclic triterpenoid saponin with broad pharmacological activities affecting collagen metabolism and modulating pathogenesis of inflammation and viral infections. The images of *Astragalus mongholicus* were provided by Dr. Heidi Heuberger (LfL, Bayerische Landesanstalt für Landwirtschaft, Freising, Germany).

This saponin is found in highest concentrations in the roots of *Astragalus plants such as Astragalus propinquus* and *Astragalus mongholicus* (milk-vetch root, Huang Qi; Figure 11). Astragaloside IV effectively inhibited NF-κB activation and prevented inflammatory gene expression in LPS-treated mice (Zhang and Frei, 2015). It further suppressed collagen production in activated HSC by inhibition of oxidative stress-related pathways (Li et al., 2013). *In vivo* data obtained in porcine-serum-induced hepatic fibrosis in rats further showed that Astragaloside IV is therapeutically beneficial and efficiently decreasing liver damage and expression of pro-fibrogenic genes (Liu et al., 2009b).

### Compound 861

This herbal compound is an extract of 10 mixed herbs including *Salvia miltiorrhiza, Astragalus mongholicus*, and *Spatholobus suberectus* (Millettia root; Ji Xue Teng) as its main components. There are several studies that show that this herbal mixture evolves anti-fibrotic effects *in vitro*, most likely by counteracting activities of individual TGF-β signal components (Wang et al., 2004, 2008; Li et al., 2008b). This mixture has been shown experimentally to be effective in suppressing fibrogenesis, degradation of collagen matrix, and attenuation of TIMP-1 expression and HSC proliferation. Moreover, a clinical open trial performed with 2000 patients showed significant improvement of hepatic damage (Wang, 2000; Wang et al., 2004). In addition, the anti-fibrotic capacity of this drug was proven in a double blind, placebo-controlled clinical trial with 136 patients with HBV-related fibrosis that were treated for 24 weeks with that drug (Yin et al., 2004). In China, compound 861 is approved by the State food and Drug Administration and broadly marketed as an anti-fibrotic agent (Zhang and Schuppan, 2014).

### Fuzheng huayu (FZHY)

The FZHY formula is a preparation that consists of six TCM herbs, namely *Salvia miltiorrhizae, Cordyceps* (Chong Cao), *Semen Persicae* (Tan Ren), *Gynostemma Pentaphyllum* (Jiaogulan), Pollen Pini (Song Hua Fen), *Fructus Schisandrae Chinensis* (Wu Wei Zi) (Liu et al., 2009a). This herbal mixture display a large variety of biological actions in hepatic fibrosis. *In vitro* it inhibited cellular activation, proliferation, and collagen secretion in HSC and protects hepatocytes from oxidative stress and apoptosis, and further reduced MMP-2/9 activities in fibrotic liver tissue (Liu et al., 2009a). It was suggested that each of the individual ingredients of FZHY evolve their specific hepatoprotective activity that in sum prevent hepatic fibrosis. *Semen Persicae* decreased hepatic hydroxyproline, *Radix Salvia Miltiorrhizae* improved liver function, while *Cordyceps* and *Gynostemma Pentaphyllum* helped to decrease serum aminotransferase activity (Liu et al., 2009a). This herbal mixture was already studied in several clinical trials. In particular, a large well-designed Chinese multicenter study in which the efficacy and safety of FZHY capsules were tested showed that FZHY has good therapeutic effects on alleviating liver fibrosis in patients suffering from chronic HBV infection without any adverse effects (Liu et al., 2005b). This drug is approved in China and is currently tested in a FDA-approved trial in patients with chronic HCV (Zhang and Schuppan, 2014).

### Biejiaruangan compound (CBJRGC)

This decoction contains *Carapax trionycis* (turtle shell) and 10 herbs (e.g., panax, read peony root, cordyceps sinensis, forsythia). Serum collected from rats that were perfused with CBJRGC decreased the proliferation and expression of desmin, synapsin, and PDGF in a HSC line (Guo et al., 2004). This drug is approved in China and was the first TCM composition that has been officially used for treating hepatic fibrosis in China (Guo et al., 2004). Together with FZHY, CBJRGC reached sales of over $30 million in China (Zhang and Schuppan, 2014).

### Other TCM medicines

There are several other TCM available that are supposed to have hepatoprotective effects. Such a formulation is Yi Guan Jian (YGJ) that is composed out of *Radix Rehmanniae* (Shen Di Huang), *Fructus Lycii* (Gou Qi Zi), *Radix Glehnaia* (Sha Shen), *Radix Ophopogonis* (Mai Men Dong), *Radix Angelicae Sinensis* (Dang Gui), and *Fructus Meliae Toosendan* (Chuan Lian Zi). In DMN-treated rats, the oral administration of this traditional TCM prevented body weight loss, collagen accumulation, and expression of TIMP-1 and α-SMA (Lin et al., 2011a). A modified YGJ (mYGJ) in which three more herbs (*Astragalus mongholicus, Trionyx sinensis*, and *Eupolyphaga sinensis*) were added to YGJ was shown to effectively induce HSC apoptosis through ROS accumulation and induction of intrinsic apoptotic pathways (Lin et al., 2011b).

### LIV.52, LIV-42, livol, liver cure, livomyn, livfit, livactine, hepatomed, jigrine, tefroli, stimuliv, livfit, silybon, liverubin

A number of other polyherbal formulations are available that were not rigorously tested in animal experimentation for their therapeutic efficacy. Although their pharmacological action is not really understood, they are offered by various companies for the treatment of viral hepatitis, drug-induced liver disease, alcohol-induced hepatotoxicity, and cholestatic jaundice. Most of these drugs originated from India where actually more than 87 medical plants are used in the preparation of 33 patented herbal formulations (Ramadoss et al., 2012). First reports however, show that these compositions might be beneficial. Livactine reduced the hepatotoxic effect of paracetamol-induced liver injury in rats (Mayuren et al., 2010). Liv.52 also known also LiverCare is composed of ingredients from *Capparis spinosa* (Capers), *Cichorium intybus* (Wild chicory), *Mandur bhasma* (Ferric Oxide Calx), *Solanum nigrum* (Black Nightshade), *Terminalia arjuna* (Arjuna), *Cassia occidentalis* (Negro coffee), *Achillea millefolium* (Yarrow), and *Tamarix gallica* (Tamarisk). Livol (IHF-100) is a purely herbal product containing different plant ingredients. Livomyn contains 18 herbals in different quantities. Both, Livol and Livomyn showed hepatoprotective effects in rats treated with CCl_4_ (Sapakal et al., 2008). Livfit and Stimuliv each contain extracts from a number of different potent hepatoprotective herbs and are produced on the basis of Ayurveda. Hepatomed contains extracts from eight herbals and offered with indications against jaundice, infective hepatitis, liver cirrhosis, and hepatospleenomegaly. The polyherbal Jirgine contains aqueous extracts of 14 plants that evolve hepatoprotective effects during TAA-induced hepatic damage (Ahmad et al., 2002). Tefroli and Liverubin are each composed of six herbal and should be effective in drug-induced hepatitis, viral and alcoholic hepatitis as well as hepatic damage. Silybon and Liverubin are made out of an extract of the seeds from *Silybum marianum* (milk thistle). Its active ingredient is silymarin that has been tested already in several experimental models of hepatic fibrosis and recently turned out to be particular suitable to block progression of fibrosis in the early stages of liver injury (Clichici et al., 2015). Other drugs such as rographolide, neoandrographolide, picroside, kutkoside, phyllanthin, and hypophyllanthin that are ingredient of Phylannthus and other plants have been suggested as hepatoprotective drugs.

### Silymarin

**Figure 12:**
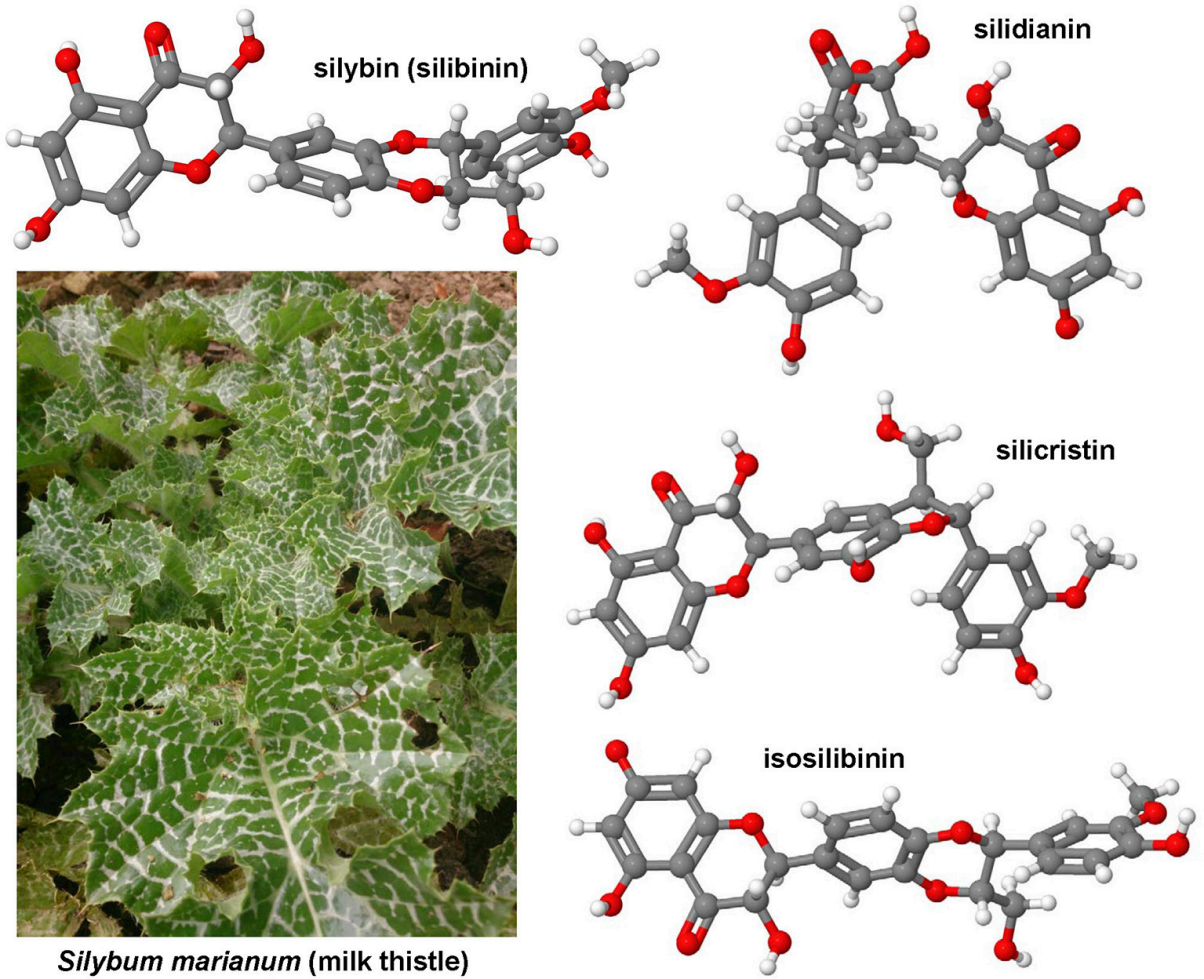
**Silymarin extract**. The extract of *silybum marianum* is a standardized herbal extract containing the four main components silibinin (CAS 22888-70-6), silidianin (CAS 29782-68-1), silicristin (CAS 33889-69-9), and isosilibinin (CAS 72581-71-6).

Silymarin is a standardized herbal extract of the milk thistle (*Silybum marianum*) that is native throughout the world. It contains four main components (silibinin, silidianin, silicristin, and isosilibinin). Curative, intragastric delivery of high doses of silymarin in rats is sufficient to increase the resolution of CCl_4_-induced fibrosis (Tsai et al., 2008) and to protect for decreased expression of fibrotic parameters such as CTGF in CCl_4_-induced hepatic fibrogenesis (Tzeng et al., 2013). Recently, gene expression profiling performed in HepG2 cells showed that silymarin (like glycyrrhizin and ursodeoxycholic acid) affect the expression of genes that are relevant for neurotransmission, and glucose- and lipid metabolisms (Hsiang et al., 2015). Silybin (also known as Silibinin; Figure 12) representing the most pharmacologically active substance of silymarin. It alone has hepatoprotective properties and is effective in preventing mitochondrial failure and inflammation (Serviddio et al., 2014).

### Xanthohumol

**Figure 13:**
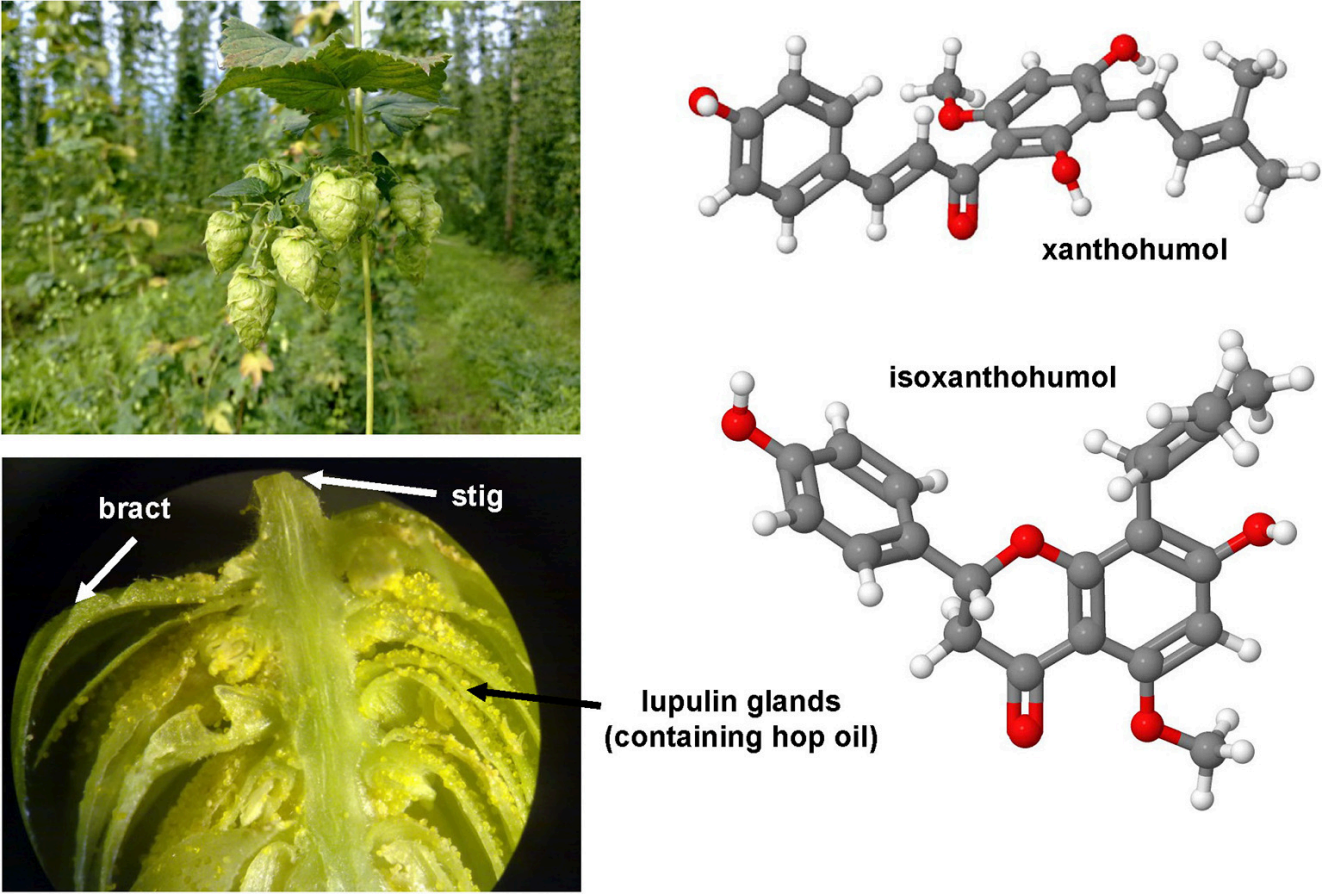
**Xanthohumol**. This principal prenylated chalcone (CAS 6754-58-1) of the hop plant (upper left panel) and its prenylated flavanone (i.e., isoxanthohumol, CAS 70872-29-6) have anti-inflammatory, antioxidant, hypoglycemic activities, and anticancer effects. Xanthohumol is included in high concentration in the hop oil that is produced from lupulin glands (lower left panel). Hop images were kindly provided by Dr. Claus Hellerbrand (Department of Internal Medicine I, University Hospital Regensburg. Regensburg, Germany).

This prenylated flavonoid (Figure 13) is the principal chalcone of the hop plant (*Humulus lupulus).* It is able to scavenge ROS and modulate the activity of a broad range of enzymes and pathways that are involved in pro-inflammatory signaling (Weiskirchen et al., 2015). It inhibits cyclooxygenases, expression of prostaglandin E2, and the NF-κB signaling pathway. Xanthohumol (XN) at 5 μM inhibits the activation of primary human HSC *in vitro*, reduces expression of pro-inflammatory genes, and induces apoptosis (Dorn et al., 2010). *In vivo*, it was shown that XN inhibited pro-inflammatory and pro-fibrogenic hepatic gene expression and suppressed hepatic NF-κB activity in mice that were subjected to CCl_4_ (Dorn et al., 2012). Several *in vitro* cell culture models, precision cut liver slices, *in vivo* experiments in rats and mice have consistently shown that XN reduces ROS formation and DNA damage in hepatocytes, has beneficial effects on fat metabolisms and protects against hepatocyte apoptosis and is considered as a cancer chemopreventive agent (Weiskirchen et al., 2015). It was therefore suggested already one decade ago that engineering of XN biosynthesis and other related prenylflavonoids from hops might be good targets for breeding or biotechnological modification with the aim to increase XN and levels for beer brewing (Stevens and Page, 2004).

## Other compounds with hepatoprotective, anti-inflammatory or anti-fibrotic effects

### Minoxidil

This drug (Supplementary Figure 12) is a FDA-approved medication for hair loss in men and women with antihypertensive activities. It is a piperidinopyrimidine derivative that was shown to inhibit fibroblast proliferation without causing cytotoxicity and confer a loss of lysyl hydroxylase activity (Murad and Pinnell, 1987). Later it was demonstrated that minoxidil is unlikely to serve as an anti-fibroticum *in vitro* (Zuurmond et al., 2005). Instead, it was noticed that minoxidil acts as a K(ATP) channel opener that accelerates DNA synthesis after partial hepatectomy without affecting the liver function (Yamasaki et al., 2005).

### Tetrandrine

This compound is the major pharmacologically active compound of *Stephania tetrandra* and its roots termed Han Fang Ji are widely used in TCM. It is a calcium channel blocker and potent smooth muscle relaxant and further has anti-inflammatory activity because it inhibits the degranulation of mast cells. Studies performed in mice showed that this drug inhibited NF-κB activity in Concanavalin A-induced hepatitis and prevented T-cell mediated liver injury (Feng et al., 2008). *In vitro* it inhibited proliferation of HCC cell lines by suppression of cell cycle progression at the G_2_/M phase (Yu and Ho, 2013) and promotes apoptosis of activated HSC (Yin et al., 2007).

### Caffeine

This water- and lipid-soluble methylxanthine alkaloid (Supplementary Figure 12) compound is a competitive receptor antagonist at all four adenosine receptors that are generally slowing down the metabolic activity of the body. A human association study conducted with 177 chronic hepatitis virus infected patients has suggested that caffeine consumption in form of regular coffee intake is associated with less severe hepatic fibrosis (Modi et al., 2010). At the cellular level, caffeine is able to increase HSC apoptosis, intracellular F-actin expression, cyclic AMP production, while inhibiting procollagen type Ic and α-SMA expression (Shim et al., 2013). In the same study, the expression of TGF-β and α-SMA in rats treated with TAA was significant lowered. These beneficial effects of coffee and caffeine were also determined in rats treated with CCl_4_ or TAA (Moreno et al., 2011; Arauz et al., 2013). Mechanistically, it is assumed that caffeine directly evolves its antifibrotic properties at a point in which either HSC activation is diminished or in which inflammatory and fibrotic processes are attenuated. However, there is another report showing that decaffeinated coffee is also fibroprotective in TAA-treated rats (Arauz et al., 2013). Moreover, some coffee (e.g., Turkish coffee) conversely impressed and potentiated liver hepatoxicity, inflammation and fibrosis in the CCl_4_ model (Poyrazoglu et al., 2008).

### Pirfenidone

This compound (Supplementary Figure 12) is approved in several countries for the treatment of idopathic pulmonary fibrosis. In this disease it prevents accumulation of inflammatory cell, proliferation of fibroblasts, expression of cytokines and ECM formation. It has however, a more general anti-fibrotic activity in lung, heart, kidney, and liver (Schaefer et al., 2011). In experimental cirrhosis that was either induced by chronic administration of CCl_4_ application, BDL surgery, or DMN in rats, pirfenidone evolved strong antioxidant properties and reduced expression of pro-fibrogenic genes and collagen, while genes associated with hepatic regeneration were increased (Tada et al., 2001; Salazar-Montes et al., 2008). Moreover, also when applied in a fully curative manner, the drug showed similar beneficial effects (García et al., 2002). In humans, this drug improved inflammation, fibrosis and steatosis in patients with chronic hepatitis C when applied for 2 years (Flores-Contreras et al., 2014). Mechanistically, an early study that applied this drug to cultured HSC demonstrated that was able to inhibit PDGF-induced activation of the Na^+^/H^+^ exchanger (Di Sario et al., 2002).

### N-acetyl-seryl-aspartyl-lysyl-proline (AcSDKP)

The tetrapeptide AcSDKP is normally present as an endogenous peptide in human plasma and circulating mononuclear cells. It is proteolytically released from its precursor thymosin-β4 by prolyl oligopeptidase (Suzuki et al., 2014). AcSDKP evolved anti-fibrotic effects in rats that were either subjected to BDL or treated with CCl_4_, most likely by direct exerting anti-fibrogenic effects on HSC (Chen et al., 2010; Zhang et al., 2012a). In addition, this substance inhibited fibroblast proliferation and collagen synthesis *in vitro* (Peng et al., 2001).

### Prostaglandins

Prostaglandins are a group of biological mediators produced by a cascade that requires cyclooxygenases and prostaglandin synthases. Individual prostaglandins have attracted much interest in many research fields because they are majorly responsible for pain and inflammation. Prostaglandin E2 and derivatives significantly delayed the deposition of hepatic collagen in nutritional injury in rats (Ruwart et al., 1988). Moreover, Prostaglandin E suppressed PDGF activity in cultured HSC (Beno and Davis, 1995) and was effective in preventing activation of HSC in *Schistosoma japonicum* infected rabbits (Zou et al., 2007).

### Fluorofenidone

This pyridone agent (Supplementary Figure 12) is also known as AKF-PD was initially introduced as a drug that exerts strong anti-fibrotic effect in kidney. Preliminary reports performed in DMN-treated rats and in different immortalized HSC cell lines (LX-2, CFSC) showed that it attenuates hepatic fibrosis by suppressing HSC proliferation and activation by blocking MAPK signaling (Peng et al., 2013b). In primary rat HSC fluorofenidone caused G_0_/G_1_ cell cycle arrest by reducing expression of cyclin D1 and cyclin E (Peng et al., 2014).

### Vitamin D

Vitamin D receptor ligands such as calcipotriol (Supplementary Figure 12) inhibit HSC activation and counteract progression of CCl_4_-induced hepatic fibrosis in mice (Ding et al., 2013). It antagonized TGF-β signaling *via* VDR/Smad3 genomic crosstalk by blocking Smad residency on chromatin and compromising acetylation of histone H3 thereby suppressing pro-fibrogenic gene expression (Ding et al., 2013). Although this anti-fibrotic activity was reproduced in the TAA model in mice, Vitamin D failed to ameliorate established fibrosis and further induced a high mortality rate in BDL rats (Abramovitch et al., 2015).

## Bile acids and FXR antagonists

Bear bile known as “Tang Ban Cao” that is highly enriched in ursodeoxycholic acid (UDCA) was applied in TCM already long before introduction of modern medicine. There it is used as an anti-inflammatory formulation reducing the effects of alcohol over-consumption and restoring liver homeostasis. Bile acids act as physiological emulgators and signal *via* a large bunch of specialized bile acid receptors such as Farnesoid X receptors (FXR), transmembrane G-protein-coupled receptors, pregnane X receptor, vitamin D receptor, and constitutive androstane receptor (Schaap et al., 2014). In hepatology, bile acid receptors are versatile drug targets. In particular, endogenous (CDCA, doxycholic acid, cholic acid, lithocholic acid) and (semi-)synthetic FXR agonists (obeticholic acid) are applied to cure various types of liver diseases (Supplementary Figure 13). In general, these substances have beneficial effects on glucose metabolisms, enhance insulin sensitivity, reduce hepatic lipogenesis, and increase β-oxidation. Moreover, FXR receptors control the synthesis of bile acid synthesis and are therefore key regulators of gut microbiota. Therefore, these receptors, their pathway and the microbiota itself provide endless therapeutic drug targets to treat hepatic inflammation and fibrosis (Sayin et al., 2013). Recently, a multicentre trial with obeticholic acid was performed showing that this drug is suitable to improve histology in patients with non-cirrhotic, non-alcoholic steatohepatitis (Neuschwander-Tetri et al., 2015).

## Fish oil and omega-3 fatty acids

**Figure 14:**
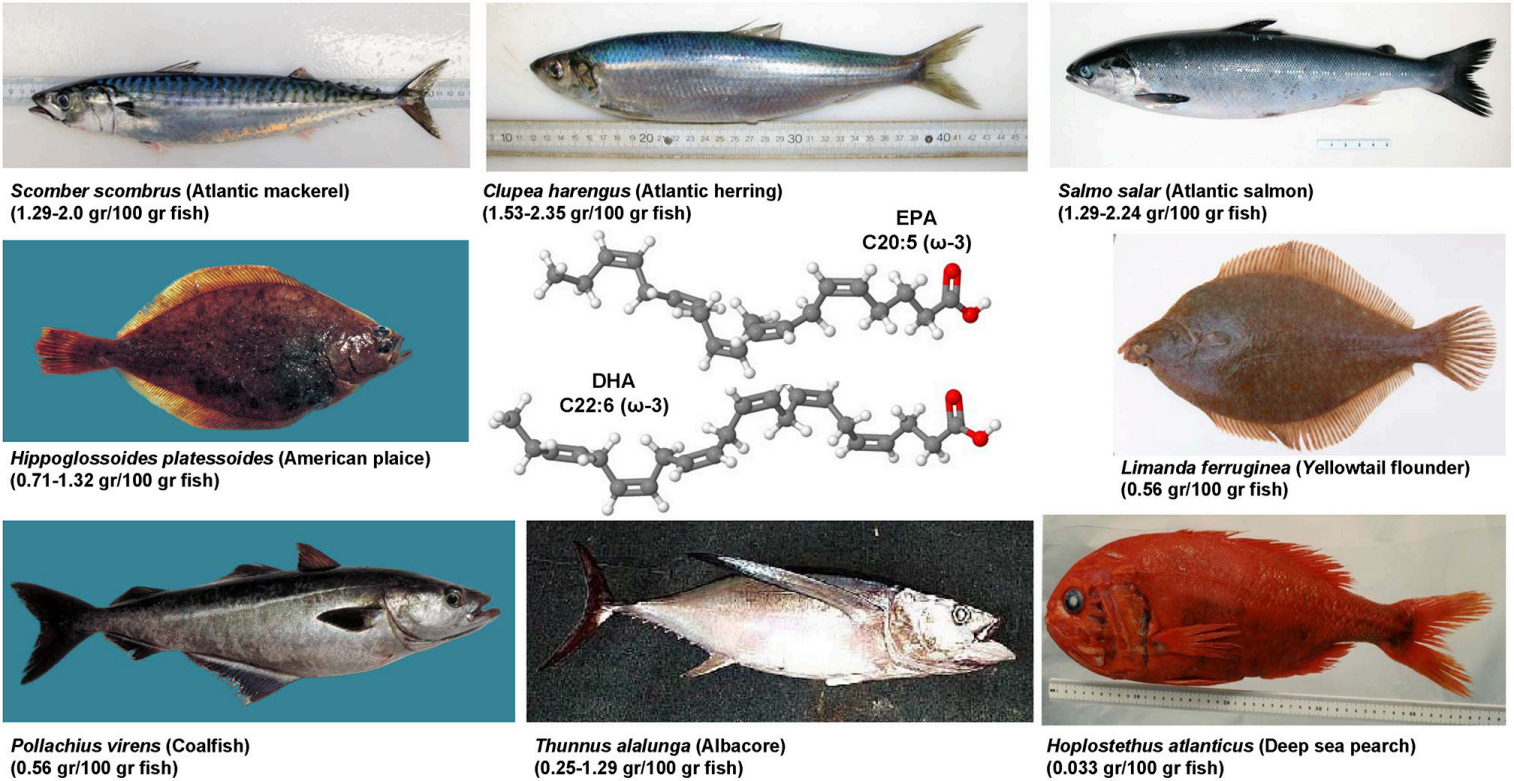
**Fish as a dietary source of ω-3 fatty acids**. Cold-water fishes such as salmon, herring, mackerel, and others are dietary sources of ω-3 fatty acids. They are characterized by a double bond at the third carbon atom from the end of the carbon chain. The depicted fishes contain high concentrations of eicosapentaenoic acid (EPA, CAS 10417-94-4) and docosahexyenoic acid (DHA, CAS 6217-54-5). The grams of ω-3 fatty acids per 100 gr fish is given in parentheses. The different fish images were published with the kind permission of Mr. Claude Nozères, Government of Canada, Department of Fisheries and Oceans (www.marinespecies.org/carms/photogallery.php).

Fish oil-based lipid emulsions that contain high concentrations of the omega-3 (ω-3) polyunsaturated fatty acids such as eicosapentaenoic acid (EPA) and docosahexaenoic acid (DHA) have an inflammation dampering effect on hepatic inflammation by interfering with the arachinodonic acid pathway and inhibition of pro-inflammatory cytokines (Schmöcker et al., 2007). Cold-water fishes are the most widely available dietary source of EPA and DHA that contain up to 2.35% ω-3 fatty acids (Figure 14). In mice, ω-3 fatty acid supplementation prevented the pathogenesis of hepatic steatosis and CCl_4_-induced hepatic fibrosis (Alwayn et al., 2005; Shaaban et al., 2014). Likewise, the Menhaden diet enriched in ω-3 fatty acids reduced hepatic necrosis and inflammation in mice that underwent BDL (Lee et al., 2008). In contrast, the dietary supplementation of fish ω-3 fatty acids in rats that underwent BDL significantly impaired liver functions suggesting that ω-3 might be of potential hazard during biliary atresia (Chen et al., 2013). In a very recent report, it was demonstrated that DHA but not EPA attenuates Western diet-induced hepatic fibrosis in mice lacking the low-density lipoprotein receptor (Lytle et al., 2015). This study further revealed that the pre-treatment of human LX-2 cells with DHA, but not other unsaturated fatty acids, blocked TGF-β-mediated induction of collagen expression. This data suggests that the different ω-3 fatty acids might have diverse biological effects on the pathogenesis of hepatic fibrosis and that more defined fish oils enriched with a specific ω-3 fatty might be therapeutically more useful.

## Marine bioactive compounds

**Figure 15:**
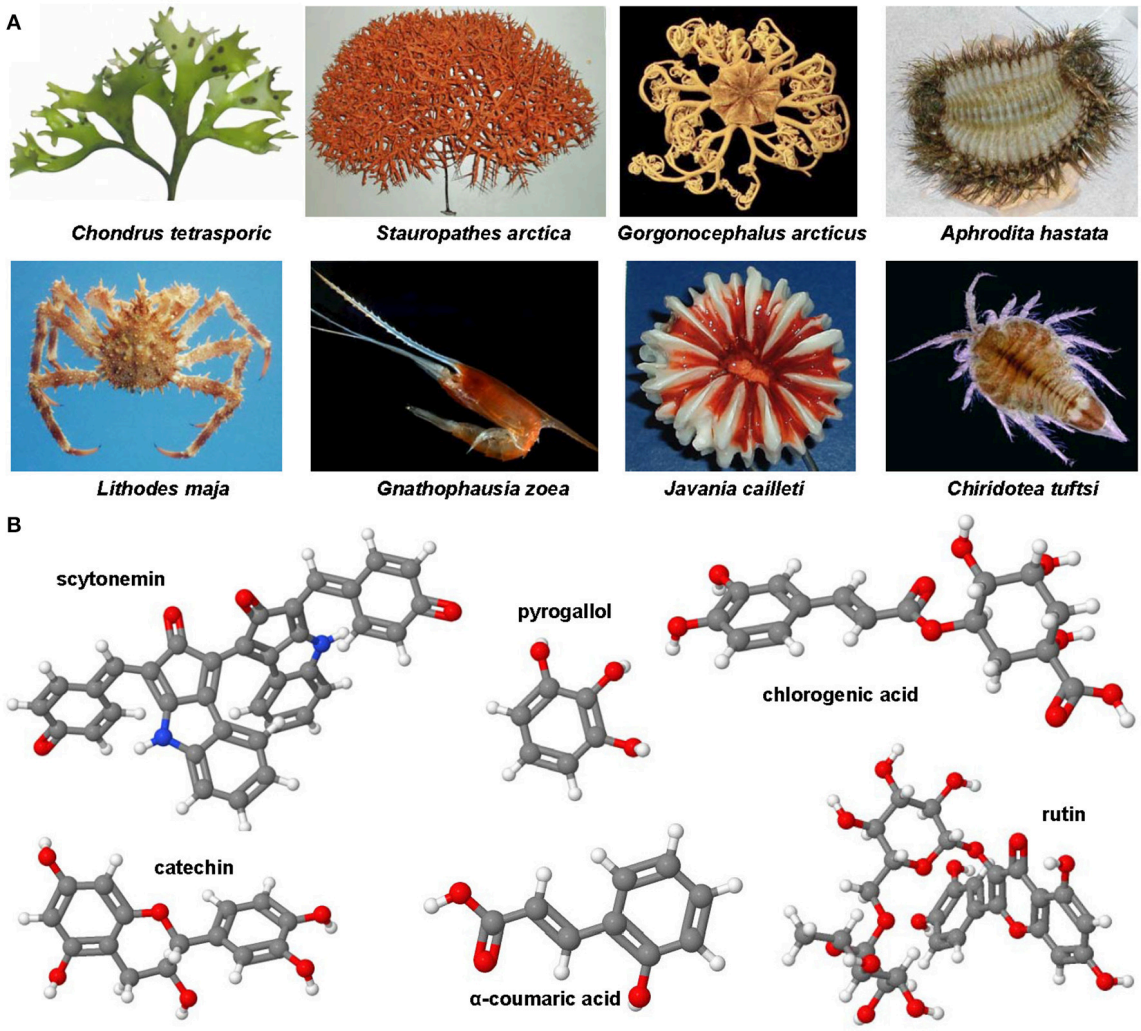
**Bioactive compounds derived from marine organisms. (A)** There is a large variety of organisms that may serve as a biological source for anti-fibrotic compounds. **(B)** Scytonemin (CAS 152075-98-4), pyrogallol (CAS 87-66-1), chlorogenic acid (CAS 327-97-9), catechin, (CAS 154-23-4), α-coumaric acid (CAS 614-60-8), and rutin (CAS 153-18-4) are substances with different structures, that however have similar anti-inflammatory and anti-fibrotic activities. The individual figures in **(A)** were published with the kind permission of Mr. Claude Nozères, Government of Canada, Department of Fisheries and Oceans (www.marinespecies.org/carms/photogallery.php).

There are an increasing number of potent drugs originating from the marine environment (Figure 15). The marine-derived bioactive compounds are structurally and biologically intriguing, and more than 30,000 compounds with unique structures and diverse pharmacological activities have been isolated so far. In hepatology research, several marine-derived compounds with antioxidant or antifibrotic potential were already tested. Some of these drugs targeting the activity of COX-1, COX-2, or phospholipase A2 thereby inhibiting eicosanoid production (e.g., pacifenol, epitacondiol, stypotriol). Others inhibit NF-κB activity (hymenialdisine), prevent TGF-β-induced Smad phosphorylation (hyrtiosal), or are direct antioxidants (fucoxanthin, astaxanthin, β-chitosan, diverse polysaccharides), or affect receptor tyrosine kinases (philinopside A). In addition, beneficial effects of diverse fish oil supplements (e.g., menhaden oil) were reported. For more details about marine hepatoprotective compounds and their beneficial effects in hepatic fibrosis were recently summarized (Nair et al., 2015).

## Conclusions and perspectives

Preclinical work from many laboratories has identified and evaluated hundreds of substances or mixtures of components showing highly beneficial effects in therapy of experimental hepatic fibrosis. They counteract intracellular ROS formation, prevent hepatic infiltration with circulating blood cells, or target pro-inflammatory and pro-fibrotic signaling pathways or mediators involved in ECM generation or turnover. Scientists and clinicians agree that it is now the time to estimate if these encouraging findings can be translated to the clinic. In particular, a first screen for safety and efficacy would provide urgently needed data on pharmacodynamics and pharmacokinetics of these “pipeline drugs.” Moreover, it will be necessary to test in volunteers if the beneficial effects of these drugs can be reproduced in humans. Of course, the transfer to human is somewhat hampered because most of these drugs were only tested in models of ongoing fibrogenesis and information about the curative effects are still missing. Therefore, in future it would be more sensible if drug candidates are tested in models of established fibrosis than to confirm their hepatoprotective or anti-fibrotic effects in other disease models. This would not only expand the knowledge of the usability of a specific drug but further helps to make science in fibrosis research more cost-effective and minimize the number of animals used in preclinical experiments requested by the 3R guiding principles proposed by Russell and Burch.

## Funding

The author's laboratory is supported by grants from the German Research Foundation (DFG, SFB/TRR57 P13) and the Interdisciplinary Centre for Clinical Research within the Faculty of Medicine at the RWTH Aachen University (IZKF, project E7-6).

### Conflict of interest statement

The author declares that the research was conducted in the absence of any commercial or financial relationships that could be construed as a potential conflict of interest.

## Supplementary material

Supplementary Figure 1 **Sulfur-containing antioxidants with therapeutic potential in hepatic fibrosis**. Glutathione (CAS 70-18-8), N-acetyl-L-cysteine (CAS 616-91-1), S-nitroso-N-acetylcysteine (CAS 56577-02-7), S-adenosyl-L-methionine (CAS 29908-03-0), S-allylcysteine (CAS 21593-77-1), bucillamine (CAS 65002-17-7), lipoic acid (CAS 1200-22-2), and taurine (CAS 107-35-7) were shown to evolve beneficial effects in experimental hepatic fibrosis.

Supplementary Figure 2 **The Nicotinamide adenine dinucleotide phosphate oxidases (NOXs) and their inhibition**. Nicotinamide (CAS 98-92-0) and nicotinic acid (CAS 59-67-6) serve as nicotinamide adenine dinucleotide precursors. NADPH oxidases (NOXs) catalyzes the transfer of electrons form NADPH to molecular oxygen thereby producing superoxide and other ROS. GKT137831 is a dual inhibitor showing high affinity for NOX1 and NOX4 but not for other isoforms, while decursin (CAS:5928-25-6) was found experimentally to suppress NOX1, NOX2 and NOX4.

Supplementary Figure 3 **HMG-CoA reductase inhibitors**. Atorvastatin (CAS 134523-00-5), fluvastatin (CAS 93957-54-1), rosuvastatin (CAS 287714-41-4), pitavastatin (CAS 147511-69-1), pravastatin (CAS 81093-37-0), lovastatin (CAS 75330-75-5), and simvastatin (CAS 79902-63-9) are presentative statins that interfere with hepatic fibrogenesis by inhibiting HMG-CoA reductase thereby preventing formation of mevalonate from HMG-CoA.

Supplementary Figure 4 **Thiazolidinediones in liver healthy**. All members of this class (e.g., pioglitazone, CAS 111025-46-8; rosiglitazone, CAS 122320-73-4) are derivatives of the parent compound 2,4-thiazolidinedione (CAS 2295-31-0). These compounds that are also known as glitazones activate nuclear receptors (PPARs) that lead to modulation of target gene transcription.

Supplementary Figure 5 **Therapeutic polysaccharides targeting galectins**. The carbohydrate-based compounds GR-MD-02 and GM-CT-01 (DAVANAT) bind to galectin-1 and galectin-3 thereby preventing its inflammation- and fibrosis-associated chemoattraction. The backbone of GM-CT-01 is a proprietary galactomannan polysaccharide composed of (1 → 4)-linked β-D-mannopyranosyl units, to which single α-D-galactopyranosyl is attached by (1 → 6)-linkage. The mannose to galactose ratio is approximately 1.7 with mean molecular weight of approximately 50 kDa. Likewise, GR-MD-02 is a proprietary galactoarabino-rhamnogalacturonate polymer that is comprised predominantly of 1,4-linked galacturonic acid, with β-galactose and arabinose side chains of approximately 50 kDa (not shown). Both polysaccharides bind with high affinity to galectins, thereby preventing their pro-inflammatory function in recruting inflammatory cells to the diseased liver tissue. The depicted human galectin structures were generated with the Ribbons software (ver 3.0) using the structure coordinates that are deposited in the RCSB Protein Data Bank under accession nos. and 1GZW (galectin-1) and 1A3K (galectin-3), respectively.

Supplementary Figure 6 **RGD-peptide and integrins**. Integrin function can be blocked by the tripeptide RGD or substances that have a similar structural arrangement such as the non-peptidic RGD mimetic SF-6,5. Both substances bind with high affinity to regions within integrins.

Supplementary Figure 7 **Inhibitors of collagen synthesis and fibroblast proliferation**. Malotilate (CAS 59937-28-9), transilast (CAS 53902-12-8), HOE 077 (CAS 128075-79-6), S 0885 (CAS 117517-22-3) β-aminopropionitrile (CAS 151-18-8), and safironil (CAS 134377-69-8) are drugs that interfere with fibroblastic proliferation and expression or synthesis of collagen.

Supplementary Figure 8 **Histone deacetylase and acetyltransferase blockers**. The *curcuma longa* ingredient curcumin (CAS 458-37-7), trichostatin A (TSA, CAS 58880-19-6), and valproic acid (CAS 99-66-1) marked under a multitude of brand names are potent histone deacetylase blocker, while the green tea ingredient epigallocatechin-3-gallate (EGCG, CAS 989-51-5) interferes with the activity of acetyltransferases.

Supplementary Figure 9 **Angiotensin receptor antagonism**. The Angiotensin axis can be blocked by ACE inhibitors (e.g., perindopril, CAS 107133-36-8), selective receptor blockers (losartan, CAS 114798-26-4; candesartan, CAS 139481-59-7), or direct renin inhibitors (e.g., aliskiren; CAS 173334-57-1).

Supplementary Figure 10 **Endothelin-1**. ET-1 (CAS 117399-94-7) is a potent vasoconstrictor involved in the formation of portal hypertension. The major biological active peptide consists of 21 residues. It is stepwise processed from a 212 aa precursor form. The mature peptide contains two disulfide bonds (disulfide bridges: 1-15 and 3-11). The X-ray structure of mature human ET-1 that is deposited in the RCSB Protein Data Base under accession no. 1EDN shows that the entire carboxy terminal tail (residues 16-21) that is crucial for receptor binding and vasoactivity foms a helix.

Supplementary Figure 11 **RTK inhibitor substances**. Numerous studies demonstrated that Imatinib (CAS 152459-95-5), nilotinib (CAS 641571-10-0), sunitinib (CAS 557795-19-4), sorafenib (CAS 284461-73-0), and orantinib (CAS 252916-29-3) have beneficial activities in hepatic fibrosis by targeting diverse receptor kinases (for details see text).

Supplementary Figure 12 **Miscellaneous compounds with hepatoprotective, anti-inflammatory or anti-fibrotic effects**. Vitamin D (CAS 67-97-0), tetrandrine (CAS 518-34-3), caffeine (CAS 58-08-2), pirfenidone (CAS 53179-13-8), and minoxidil (CAS 38304-91-5) are compounds that showed anti-inflammatory and anti-fibrotic activities in various fibrosis models.

Supplementary Figure 13 **Bile acids and FXR antagonists**. Obeticholic acid (OCA, CAS 459789-99-2) is a semi-synthetic bile acid derivate related to Ursodeoxycholate (UDCA, CAS 128-13-2) used to treat cholestatic liver disease. In contrast to UDCA, OCA and other bile acids (cholic acid, lithocholic acid, and deoxycholic acid) are FXR ligands.

## Abbreviations

ACE: angiotensin-converting enzyme
α-SMA: α-smooth muscle actin
CAS: Chemical Abstracts Service
CCl_4_: carbon tetrachloride
CTGF: connective tissue growth factor
DHA: docosahexaenoic acid
DMN: dimethylnitrosamine
ECM: extracellular matrix
EGCG: Epigallocatechin-3-gallate
EGF: epidermal growth factor
EPA: eicosapentaenoic acid
FGF: fibroblast growth factor
FZHY: Fuzheng Huayu
GSH: reduced glutathione
HAT: histone acetyl transferases
HCC: hepatocellular carcinoma
HDAC: Histone deacetylase(s)
HGF: hepatocyte growth factor
HMG-CoA: hydroxy-3.methly-glutaryl-CoA
HSC: hepatic stellate cell(s)
IFN: interferon
IGF: insulin-like growth factor
IL(s): interleukin(s)
LOXL2: Lysyl oxidase-like-2
MCD: methionine-choline-deficient
MMP: metalloproteinase(s)
NAC: N-acetyl-L-cysteine
NASH: non-alcoholic steatohepatitis
NOX: NADPH oxidases
Nrf2: nuclear factor erythroid-2-related factor 2
PDGF: platelet-derived growth factor
ROS: reactive oxygen species
RTK: receptor tyrosine kinases
SAC: S-allylcysteine
SAM: S-adenosyl-L-methionine
SNAC: S-Nitroso-N-acetylcysteine
SPARC: secreted protein acidic and rich in cysteine
TAA: thioacetamide
TGF-β: transforming growth factor-β
TNF-α: tumor necrosis factor-α
VEGF: vascular endothelial growth factor
XN: xanthohumol.

## References

[B1] Abdelazim S. A. Darwish H. A. Ali S. A. Rizk M. Z. Kadry M. O. (2015). Potential antifibrotic and angiostatic impact of idebenone, carnosine and vitamin E in nano-sized titanium dioxide-induced liver injury. Cell. Physiol. Biochem. 35, 2402–2411. 10.1159/000374041 25896716

[B2] Abdelaziz R. R. Elkashef W. F. Said E. (2015). Tranilast reduces serum IL-6 and IL-13 and protects against thioacetamide-induced acute liver injury and hepatic encephalopathy. Environ. Toxicol. Pharmacol. 40, 259–267. 10.1016/j.etap.2015.06.019 26164743

[B3] Abramovitch S. Sharvit E. Weisman Y. Bentov A. Brazowski E. Cohen G.. (2015). Vitamin D inhibits development of liver fibrosis in an animal model but cannot ameliorate established cirrhosis. Am. J. Physiol. Gastrointest. Liver Physiol. 308, G112–G120. 10.1152/ajpgi.00132.2013 25214398

[B4] Abreu J. G. Ketpura N. I. Reversade B. De Robertis E. M. (2002). Connective-tissue growth factor (CTGF) modulates cell signalling by BMP and TGF-β. Nat. Cell Biol. 4, 599–604. 10.1038/ncb826 12134160 PMC2387275

[B5] Adikwu E. Deo O. (2013). Hepatoprotective effect of vitamin C (ascorbic acid). Pharmacol. Pharm. 4, 84–92. 10.4236/pp.2013.41012

[B6] Aher J. S. Khan S. Jain S. Tikoo K. Jena G. (2015). Valproate ameliorates thioacetamide-induced fibrosis by hepatic stellate cell inactivation. Hum. Exp. Toxicol. 34, 44–55. 10.1177/0960327114531992 24812151

[B7] Ahmad A. Pillai K. K. Najmi A. K. Ahmad S. J. Pal S. N. Balani D. K. (2002). Evaluation of hepatoprotective potential of jigrine post-treatment against thioacetamide induced hepatic damage. J. Ethnopharmacol. 79, 35–41. 10.1016/S0378-8741(01)00349-X 11744293

[B8] Ala-Kokko L. Stenbäck F. Ryhänen L. (1989). Preventive effect of malotilate on dimethylnitrosamine-induced liver fibrosis in the rat. J. Lab. Clin. Med. 113, 177–183. 2915182

[B9] Al-Attar A. M. (2011). Hepatoprotective influence of vitamin C on thioacetamide-induced liver cirrhosis in Wistar male rats. Pharmacol. Toxicol. 6, 218–233. 10.3923/jpt.2011.218.233

[B10] Alwayn I. P. Gura K. Nosé V. Zausche B. Javid P. Garza J.. (2005). Omega-3 fatty acid supplementation prevents hepatic steatosis in a murine model of nonalcoholic fatty liver disease. Pediatr. Res. 57, 445–452. 10.1203/01.PDR.0000153672.43030.75 15659701

[B11] Amália P. M. Possa M. N. Augusto M. C. Francisca L. S. (2007). Quercetin prevents oxidative stress in cirrhotic rats. Dig. Dis. Sci. 52, 2616–2621. 10.1007/s10620-007-9748-x 17431769

[B12] Arauz J. Moreno M. G. Cortés-Reynosa P. Salazar E. P. Muriel P. (2013). Coffee attenuates fibrosis by decreasing the expression of TGF-β and CTGF in a murine model of liver damage. J. Appl. Toxicol. 33, 970–979. 10.1002/jat.2788 22899499

[B13] Arauz J. Rivera-Espinoza Y. Shibayama M. Favari L. Flores-Beltrán R. E. Muriel P. (2015). Nicotinic acid prevents experimental liver fibrosis by attenuating the prooxidant process. Int. Immunopharmacol. 28, 244–251. 10.1016/j.intimp.2015.05.045 26093271

[B14] Argo C. K. Loria P. Caldwell S. H. Lonardo A. (2008). Statins in liver disease: a molehill, an iceberg, or neither? Hepatology 48, 662–669. 10.1002/hep.22402 18666246

[B15] Arias M. Sauer-Lehnen S. Treptau J. Janoschek N. Theuerkauf I. Buettner R.. (2003). Adenoviral expression of a transforming growth factor-β1 antisense mRNA is effective in preventing liver fibrosis in bile-duct ligated rats. BMC Gastroenterol. 3, 29. 10.1186/1471-230X-3-29 14565855 PMC270053

[B16] Atorrasagasti C. Aquino J. B. Hofman L. Alaniz L. Malvicini M. Garcia M.. (2011). SPARC downregulation attenuates the profibrogenic response of hepatic stellate cells induced by TGF-β1 and PDGF. Am. J. Physiol. Gastrointest. Liver Physiol. 300, G739–G748. 10.1152/ajpgi.00316.2010 21311029 PMC3094149

[B17] Atorrasagasti C. Peixoto E. Aquino J. B. Kippes N. Malvicini M. Alaniz L.. (2013). Lack of the matricellular protein SPARC (secreted protein, acidic and rich in cysteine) attenuates liver fibrogenesis in mice. PLoS ONE 8:e54962. 10.1371/journal.pone.0054962 23408952 PMC3569438

[B18] Barry-Hamilton V. Spangler R. Marshall D. McCauley S. Rodriguez H. M. Oyasu M.. (2010). Allosteric inhibition of lysyl oxidase-like-2 impedes the development of a pathologic microenvironment. Nat. Med. 16, 1009–1017. 10.1038/nm.2208 20818376

[B19] Bataller R. Gäbele E. Parsons C. J. Morris T. Yang L. Schoonhoven R.. (2005). Systemic infusion of angiotensin II exacerbates liver fibrosis in bile duct-ligated rats. Hepatology 41, 1046–1055. 10.1002/hep.20665 15841463

[B20] Bataller R. Schwabe R. F. Choi Y. H. Yang L. Paik Y. H. Lindquist J.. (2003). Schoonhoven R, Hagedorn CH, Lemasters JJ, Brenner DA. NADPH oxidase signal transduces angiotensin II in hepatic stellate cells and is critical in hepatic fibrosis. J. Clin. Invest. 112, 1383–1394. 10.1172/JCI18212 14597764 PMC228420

[B21] Baumgardner J. N. Shankar K. Hennings L. Albano E. Badger T. M. Ronis M. J. (2008). N-acetylcysteine attenuates progression of liver pathology in a rat model of nonalcoholic steatohepatitis. J. Nutr. 138, 1872–1879. 18806095 10.1093/jn/138.10.1872 PMC2935161

[B22] Beno D. W. Davis B. H. (1995). Prostaglandin E suppresses hepatic fibrosis: section I. The *in vivo* approach; Section II. The *in vitro* approach. Am. J. Ther. 2, 687–705. 10.1097/00045391-199509000-00018 11854847

[B23] Beyer C. Distler J. H. (2013). Tyrosine kinase signaling in fibrotic disorders: translation of basic research to human disease. Biochim. Biophys. Acta 1832, 897–904. 10.1016/j.bbadis.2012.06.008 22728287

[B24] Bickel M. Baader E. Brocks D. G. Engelbart K. Günzler V. Schmidts H. L.. (1991). Beneficial effects of inhibitors of prolyl 4-hydroxylase in CCl4-induced fibrosis of the liver in rats. J. Hepatol. 13, S26–S33. 10.1016/0168-8278(91)90005-v 1667666

[B25] Bickel M. Baringhaus K. H. Gerl M. Günzler V. Kanta J. Schmidts L.. (1998). Selective inhibition of hepatic collagen accumulation in experimental liver fibrosis in rats by a new prolyl 4-hydroxylase inhibitor. Hepatology 28, 404–411. 10.1002/hep.510280217 9696004

[B26] Boettcher E. Csako G. Pucino F. Wesley R. Loomba R. (2012). Meta-analysis: pioglitazone improves liver histology and fibrosis in patients with non-alcoholic steatohepatitis. Aliment. Pharmacol. Ther. 35, 66–75. 10.1111/j.1365-2036.2011.04912.x 22050199 PMC3488596

[B27] Bondareva A. Downey C. M. Ayres F. Liu W. Boyd S. K. Hallgrimsson B.. (2009). The lysyl oxidase inhibitor, beta-aminopropionitrile, diminishes the metastatic colonization potential of circulating breast cancer cells. PLoS ONE 4:e5620. 10.1371/journal.pone.0005620 19440335 PMC2680032

[B28] Bonefeld K. Møller S. (2011). Insulin-like growth factor-I and the liver. Liver Int. 31, 911–919. 10.1111/j.1478-3231.2010.02428.x 21733081

[B29] Borkham-Kamphorst E. Alexi P. Tihaa L. Haas U. Weiskirchen R. (2015b). Platelet-derived growth factor-D modulates extracellular matrix homeostasis and remodeling through TIMP-1 induction and attenuation of MMP-2 and MMP-9 gelatinase activities. Biochem Biophys Res Commun. 457, 307–313. 10.1016/j.bbrc.2014.12.106 25576870

[B30] Borkham-Kamphorst E. Herrmann J. Stoll D. Treptau J. Gressner A. M. Weiskirchen R. (2004). Dominant-negative soluble PDGF-beta receptor inhibits hepatic stellate cell activation and attenuates liver fibrosis. Lab. Invest. 84, 766–777. 10.1038/labinvest.3700094 15077122

[B31] Borkham-Kamphorst E. Kovalenko E. van Roeyen C. R. Gassler N. Bomble M. Ostendorf T.. (2008). Platelet-derived growth factor isoform expression in carbon tetrachloride-induced chronic liver injury. Lab. Invest. 88, 1090–1100. 10.1038/labinvest.2008.71 18663351

[B32] Borkham-Kamphorst E. Meurer S. K. van de Leur E. Haas U. Tihaa L. Weiskirchen R. (2015a). PDGF-D signaling in portal myofibroblasts and hepatic stellate cells proves identical to PDGF-B via both PDGF receptor type α and β. Cell Signal. 27, 1305–1314. 10.1016/j.cellsig.2015.03.012 25819339

[B33] Borkham-Kamphorst E. Schaffrath C. van de Leur E. Haas U. Tihaa L. Meurer S. K.. (2014). The anti-fibrotic effects of CCN1/CYR61 in primary portal myofibroblasts are mediated through induction of reactive oxygen species resulting in cellular senescence, apoptosis and attenuated TGF-β signaling. Biochim Biophys Acta 1843, 902–914. 10.1016/j.bbamcr.2014.01.023 24487063

[B34] Borrás C. Gambini J. López-Grueso R. Pallardó F. V. Viña J. (2010). Direct antioxidant and protective effect of estradiol on isolated mitochondria. Biochim. Biophys. Acta. 1802, 205–211. 10.1016/j.bbadis.2009.09.007 19751829

[B35] Bors W. Michel C. Stettmaier K. Lu Y. Foo L. Y. (2004). Antioxidant mechanisms of polyphenolic caffeic acid oligomers, constituents of Salvia officinalis. Biol. Res. 37, 301–311. 10.4067/S0716-97602004000200017 15455660

[B36] Bruck R. Hershkoviz R. Lider O. Aeed H. Zaidel L. Matas Z.. (1996). Inhibition of experimentally-induced liver cirrhosis in rats by a nonpeptidic mimetic of the extracellular matrix-associated Arg-Gly-Asp epitope. J. Hepatol. 24, 731–738. 10.1016/S0168-8278(96)80270-4 8835749

[B37] Bührer M. Le Cotonnec J. Y. Wermeille M. Bircher J. (1986). Treatment of liver disease with malotilate. A pharmacokinetic and pharmacodynamic phase II study in cirrhosis. Eur. J. Clin. Pharmacol. 30, 407–416. 10.1007/BF00607952 3743616

[B38] Calvaruso V. Maimone S. Gatt A. Tuddenham E. Thursz M. Pinzani M.. (2008). Coagulation and fibrosis in chronic liver disease. Gut 57, 1722–1727. 10.1136/gut.2008.150748 19022928

[B39] Camino A. M. Atorrasagasti C. Maccio D. Prada F. Salvatierra E. Rizzo M.. (2008). Adenovirus-mediated inhibition of SPARC attenuates liver fibrosis in rats. J. Gene Med. 10, 993–1004. 10.1002/jgm.1228 18615449

[B40] Campbell J. S. Hughes S. D. Gilbertson D. G. Palmer T. E. Holdren M. S. Haran A. C.. (2005). Platelet-derived growth factor C induces liver fibrosis, steatosis, and hepatocellular carcinoma. Proc. Natl. Acad. Sci. U.S.A. 102, 3389–3394. 10.1073/pnas.0409722102 15728360 PMC552940

[B41] Cao Q. Mak K. M. Lieber C. S. (2002). Dilinoleoylphosphatidylcholine prevents transforming growth factor-beta1-mediated collagen accumulation in cultured rat hepatic stellate cells. J. Lab. Clin. Med. 139, 202–210. 10.1067/mlc.2002.121853 12024107

[B42] Chai N. L. Fu Q. Shi H. Cai C. H. Wan J. Xu S. P.. (2012). Oxymatrine liposome attenuates hepatic fibrosis via targeting hepatic stellate cells. World J. Gastroenterol. 18, 4199–4206. 10.3748/wjg.v18.i31.4199 22919254 PMC3422802

[B43] Chainani-Wu N. (2003). Safety and anti-inflammatory activity of curcumin: a component of tumeric (*Curcuma longa*). J. Altern. Complement Med. 9, 161–168. 10.1089/107555303321223035 12676044

[B44] Chang B. Nishikawa M. Nishiguchi S. Inoue M. (2005). L-carnitine inhibits hepatocarcinogenesis via protection of mitochondria. Int. J. Cancer 113, 719–729. 10.1002/ijc.20636 15499623

[B45] Chávez E. Reyes-Gordillo K. Segovia J. Shibayama M. Tsutsumi V. Vergara P.. (2008). Resveratrol prevents fibrosis, NF-kappaB activation and TGF-β increases induced by chronic CCl4 treatment in rats. J. Appl. Toxicol. 28, 35–43. 10.1002/jat.1249 17429801

[B46] Chen C. C. Ho C. Y. Chaung H. C. Tain Y. L. Hsieh C. S. Kuo F. Y.. (2013). Fish omega-3 fatty acids induce liver fibrosis in the treatment of bile duct-ligated rats. Dig. Dis. Sci. 58, 440–447. 10.1007/s10620-012-2489-5 23203732

[B47] Chen Y. W. Liu B. W. Zhang Y. J. Chen Y. W. Dong G. F. Ding X. D.. (2010). Preservation of basal AcSDKP attenuates carbon tetrachloride-induced fibrosis in the rat liver. J. Hepatol. 53, 528–536. 10.1016/j.jhep.2010.03.027 20646773

[B48] Cheng A. L. Shen Y. C. Zhu A. X. (2011). Targeting fibroblast growth factor receptor signaling in hepatocellular carcinoma. Oncology 81, 372–380. 10.1159/000335472 22269894

[B49] Chiang D. J. Pritchard M. T. Nagy L. E. (2011). Obesity, diabetes mellitus, and liver fibrosis. Am. J. Physiol. Gastrointest. Liver Physiol. 300, G697–G702. 10.1152/ajpgi.00426.2010 21350183 PMC3094133

[B50] Choi H. S. Kang J. W. Lee S. M. (2015). Melatonin attenuates carbon tetrachloride-induced liver fibrosis via inhibition of necroptosis. Transl. Res. 166, 292–303. 10.1016/j.trsl.2015.04.002 25936762

[B51] Choi K. C. Jung M. G. Lee Y. H. Yoon J. C. Kwon S. H. Kang H. B.. (2009). Epigallocatechin-3-gallate, a histone acetyltransferase inhibitor, inhibits EBV-induced B lymphocyte transformation via suppression of RelA acetylation. Cancer Res. 69, 583–592. 10.1158/0008-5472.CAN-08-2442 19147572

[B52] Choi Y. J. Kim da H. Kim S. J. Kim J. Jeong S. I. Chung C. H.. (2014). Decursin attenuates hepatic fibrogenesis through interrupting TGF-β-mediated NAD(P)H oxidase activation and Smad signaling *in vivo* and *in vitro*. Life Sci. 108, 94–103. 10.1016/j.lfs.2014.05.012 24880074

[B53] Chtourou Y. Fetoui H. Jemai R. Ben Slima A. Makni M. Gdoura R. (2015). Naringenin reduces cholesterol-induced hepatic inflammation in rats by modulating matrix metalloproteinases-2, 9 via inhibition of nuclear factor κB pathway. Eur. J. Pharmacol. 746, 96–105. 10.1016/j.ejphar.2014.10.027 25446569

[B54] Clichici S. Olteanu D. Nagy A. L. Oros A. Filip A. Mircea P. A. (2015). Silymarin inhibits the progression of fibrosis in the early stages of liver injury in CCl_4_-treated rats. J. Med. Food 18, 290–298. 10.1089/jmf.2013.0179 25133972

[B55] Cong M. Liu T. Wang P. Fan X. Yang A. Bai Y.. (2013). Antifibrotic effects of a recombinant adeno-associated virus carrying small interfering RNA targeting TIMP-1 in rat liver fibrosis. Am. J. Pathol. 182, 1607–1616. 10.1016/j.ajpath.2013.01.036 23474083

[B56] Corpechot C. Barbu V. Wendum D. Kinnman N. Rey C. Poupon R.. (2002). Hypoxia-induced VEGF and collagen I expressions are associated with angiogenesis and fibrogenesis in experimental cirrhosis. Hepatology 35, 1010–1021. 10.1053/jhep.2002.32524 11981751

[B57] Crespo I. San-Miguel B. Fernández A. Ortiz de Urbina J. González-Gallego J. Tuñón M. J. (2015). Melatonin limits the expression of profibrogenic genes and ameliorates the progression of hepatic fibrosis in mice. Transl. Res. 165, 346–357. 10.1016/j.trsl.2014.10.003 25445210

[B58] Crosas-Molist E. Fabregat I. (2015). Role of NADPH oxidases in the redox biology of liver fibrosis. Redox Biol. 6, 106–111. 10.1016/j.redox.2015.07.005 26204504 PMC4804101

[B59] Czochra P. Klopcic B. Meyer E. Herkel J. Garcia-Lazaro J. F. Thieringer F.. (2006). Liver fibrosis induced by hepatic overexpression of PDGF-B in transgenic mice. J. Hepatol. 45, 419–428. 10.1016/j.jhep.2006.04.010 16842882

[B60] Dang Z. C. Audinot V. Papapoulos S. E. Boutin J. A. Löwik C. W. (2003). Peroxisome proliferator-activated receptor gamma (PPARgamma) as a molecular target for the soy phytoestrogen genistein. J. Biol. Chem. 278, 962–967. 10.1074/jbc.M209483200 12421816

[B61] Das S. K. DesAulniers J. Dyck J. R. Kassiri Z. Oudit G. Y. (2015). Resveratrol mediates therapeutic hepatic effects in acquired and genetic murine models of iron-overload. Liver Int. [Epub ahead of print]. 10.1111/liv.12893 26077449

[B62] De Bleser P. J. Xu G. Rombouts K. Rogiers V. Geerts A. (1999). Glutathione levels discriminate between oxidative stress and transforming growth factor-beta signaling in activated rat hepatic stellate cells. J. Biol. Chem. 274, 33881–33887. 10.1074/jbc.274.48.33881 10567349

[B63] de Gouville A. C. Boullay V. Krysa G. Pilot J. Brusq J. M. Loriolle F.. (2005). Inhibition of TGF-beta signaling by an ALK5 inhibitor protects rats from dimethylnitrosamine-induced liver fibrosis. Br. J. Pharmacol. 145, 166–177. 10.1038/sj.bjp.0706172 15723089 PMC1576127

[B64] Dekel R. Zvibel I. Brill S. Brazovsky E. Halpern Z. Oren R. (2003). Gliotoxin ameliorates development of fibrosis and cirrhosis in a thioacetamide rat model. Dig. Dis. Sci. 48, 1642–1647. 10.1023/A:1024792529601 12924662

[B65] De Minicis S. Seki E. Paik Y. H. Osterreicher C. H. Kodama Y. Kluwe J.. (2010). Role and cellular source of nicotinamide adenine dinucleotide phosphate oxidase in hepatic fibrosis. Hepatology 52, 1420–1430. 10.1002/hep.23804 20690191 PMC2947612

[B66] Demiroren K. Dogan Y. Kocamaz H. Ozercan I. H. Ilhan S. Ustundag B.. (2014). Protective effects of L-carnitine, N-acetylcysteine and genistein in an experimental model of liver fibrosis. Clin. Res. Hepatol. Gastroenterol. 38, 63–72. 10.1016/j.clinre.2013.08.014 24239319

[B67] Deng Y. R. Ma H. D. Tsuneyama K. Yang W. Wang Y. H. Lu F. T.. (2013). STAT3-mediated attenuation of CCl4-induced mouse liver fibrosis by the protein kinase inhibitor sorafenib. J. Autoimmun. 46, 25–34. 10.1016/j.jaut.2013.07.008 23948302

[B68] Devi S. L. Anuradha C. V. (2010). Oxidative and nitrosative stress in experimental rat liver fibrosis: protective effect of taurine. Environ. Toxicol. Pharmacol. 29, 104–110. 10.1016/j.etap.2009.11.005 21787590

[B69] Devi S. L. Viswanathan P. Anuradha C. V. (2010). Regression of liver fibrosis by taurine in rats fed alcohol: effects on collagen accumulation, selected cytokines and stellate cell activation. Eur. J. Pharmacol. 647, 161–170. 10.1016/j.ejphar.2010.08.011 20813107

[B70] Ding N. Yu R. T. Subramaniam N. Sherman M. H. Wilson C. Rao R.. (2013). A vitamin D receptor/SMAD genomic circuit gates hepatic fibrotic response. Cell 153, 601–613. 10.1016/j.cell.2013.03.028 23622244 PMC3673534

[B71] Ding X. Saxena N. K. Lin S. Xu A. Srinivasan S. Anania F. A. (2005). The roles of leptin and adiponectin: a novel paradigm in adipocytokine regulation of liver fibrosis and stellate cell biology. Am. J. Pathol. 166, 1655–1669. 10.1016/S0002-9440(10)62476-5 15920151 PMC1602420

[B72] Ding Y. Sun X. Chen Y. Deng Y. Qian K. (2015). Epigallocatechin gallate attenuated non-alcoholic steatohepatitis induced by methionine- and choline-deficient diet. Eur. J. Pharmacol. 761, 405–412. 10.1016/j.ejphar.2015.05.005 25967348

[B73] Di Sario A. Bendia E. Svegliati Baroni G. Ridolfi F. Casini A. Ceni E.. (2002). Effect of pirfenidone on rat hepatic stellate cell proliferation and collagen production. J. Hepatol. 37, 584–591. 10.1016/S0168-8278(02)00245-3 12399223

[B74] Dokmanovic M. Clarke C. Marks P. A. (2007). Histone deacetylase inhibitors: overview and perspectives. Mol. Cancer Res. 5, 981–989. 10.1158/1541-7786.MCR-07-0324 17951399

[B75] Domitrović R. Jakovac H. Tomac J. Sain I. (2009). Liver fibrosis in mice induced by carbon tetrachloride and its reversion by luteolin. Toxicol. Appl. Pharmacol. 241, 311–321. 10.1016/j.taap.2009.09.001 19747501

[B76] Dorn C. Heilmann J. Hellerbrand C. (2012). Protective effect of xanthohumol on toxin-induced liver inflammation and fibrosis. Int. J. Clin. Exp. Pathol. 5, 29–36. 10.1055/s-0031-1295738 22295144 PMC3267483

[B77] Dorn C. Kraus B. Motyl M. Weiss T. S. Gehrig M. Schölmerich J.. (2010). Xanthohumol, a chalcon derived from hops, inhibits hepatic inflammation and fibrosis. Mol. Nutr. Food Res. 54, S205–S213. 10.1002/mnfr.200900314 20087858

[B78] Du G. J. Zhang Z. Wen X. D. Yu C. Calway T. Yuan C. S.. (2012). Epigallocatechin Gallate (EGCG) is the most effective cancer chemopreventive polyphenol in green tea. Nutrients 4, 1679–1691. 10.3390/nu4111679 23201840 PMC3509513

[B79] Du S. L. Pan H. Lu W. Y. Wang J. Wu J. Wang J. Y. (2007). Cyclic Arg-Gly-Asp peptide-labeled liposomes for targeting drug therapy of hepatic fibrosis in rats. J. Pharmacol. Exp. Ther. 322, 560–568. 10.1124/jpet.107.122481 17510318

[B80] Duplantier J. G. Dubuisson L. Senant N. Freyburger G. Laurendeau I. Herbert J. M.. (2004). A role for thrombin in liver fibrosis. Gut 53, 1682–1687. 10.1136/gut.2003.032136 15479692 PMC1774287

[B81] Dvorak Z. Ulrichova J. Pichard-Garcia L. Modriansky M. Maurel P. (2002). Comparative effect of colchicine and colchiceine on cytotoxicity and CYP gene expression in primary human hepatocytes. Toxicol In Vitro 16, 219–227. 10.1016/S.0887-2333(02)00004-8 12020594

[B82] EASL. European Association for Study of Liver (2015). EASL Recommendations on Treatment of Hepatitis C. J. Hepatol. 63, 199–236. 10.1016/j.jhep.2015.03.025 25911336

[B83] Efe C. (2013). Drug induced autoimmune hepatitis and TNF-α blocking agents: is there a real relationship? Autoimmun. Rev. 12, 337–339. 10.1016/j.autrev.2012.03.010 22841985

[B84] Elinav E. Ali M. Bruck R. Brazowski E. Phillips A. Shapira Y.. (2009). Competitive inhibition of leptin signaling results in amelioration of liver fibrosis through modulation of stellate cell function. Hepatology 49, 278–286. 10.1002/hep.22584 19065677

[B85] El-Karef A. Yoshida T. Gabazza E. C. Nishioka T. Inada H. Sakakura T.. (2007). Deficiency of tenascin-C attenuates liver fibrosis in immune-mediated chronic hepatitis in mice. J. Pathol. 211, 86–94. 10.1002/path.2099 17121418

[B86] El Taghdouini A. Najimi M. Sancho-Bru P. Sokal E. van Grunsven L. A. (2015). *In vitro* reversion of activated primary human hepatic stellate cells. Fibrogenesis Tissue Repair 8:14. 10.1186/s13069-015-0031-z 26251672 PMC4527231

[B87] EU Parliament (2010). Directive 2010/63/EU of the European Parliament and of the Council on the Protection of Animals Used for Scientific Purposes. Official Journal of the European Union 2010. L276/33–L276/79 (22 September 2010). Available online at: http://eur-lex.europa.eu/legal-content/EN/TXT/?uri=CELEX%3A32010L0063

[B88] EU Parliament (2013). Seventh Report from the Commission to the Council and the European Parliament on the Statistics on the Number of Animals Used for Experimental and Other Scientific Purposes in the Member States of the European Union in the Year 2011 {WD(2013) 497} (5 December 2013). Available online at: http://eur-lex.europa.eu/legal-content/EN/TXT/?uri=CELEX:52013DC0859

[B89] Feng D. Mei Y. Wang Y. Zhang B. Wang C. Xu L. (2008). Tetrandrine protects mice from concanavalin A-induced hepatitis through inhibiting NF-kappaB activation. Immunol Lett. 121, 127–133. 10.1016/j.imlet.2008.10.001 18992279

[B90] Ferguson B. S. McKinsey T. A. (2015). Non-sirtuin histone deacetylases in the control of cardiac aging. J. Mol. Cell Cardiol. 83, 14–20. 10.1016/j.yjmcc.2015.03.010 25791169 PMC4459895

[B91] Fiorucci S. Antonelli E. Distrutti E. Severino B. Fiorentina R. Baldoni M.. (2004). PAR1 antagonism protects against experimental liver fibrosis. Role of proteinase receptors in stellate cell activation. Hepatology 39, 365–375. 10.1002/hep.20054 14767989

[B92] Flores-Contreras L. Sandoval-Rodríguez A. S. Mena-Enriquez M. G. Lucano-Landeros S. Arellano-Olivera I. Alvarez-Álvarez A.. (2014). Treatment with pirfenidone for two years decreases fibrosis, cytokine levels and enhances CB2 gene expression in patients with chronic hepatitis C. BMC Gastroenterol. 14:131. 10.1186/1471-230X-14-131 25064094 PMC4236537

[B93] Foo N. P. Lin S. H. Lee Y. H. Wu M. J. Wang Y. J. (2011). α-Lipoic acid inhibits liver fibrosis through the attenuation of ROS-triggered signaling in hepatic stellate cells activated by PDGF and TGF-β. Toxicology. 282, 39–46. 10.1016/j.tox.2011.01.009 21251946

[B94] Friedman S. L. (2008). Hepatic stellate cells: protean, multifunctional, and enigmatic cells of the liver. Physiol. Rev. 88, 125–172. 10.1152/physrev.00013.2007 18195085 PMC2888531

[B95] Frizell E. Liu S. L. Abraham A. Ozaki I. Eghbali M. Sage E. H.. (1995). Expression of SPARC in normal and fibrotic livers. Hepatology 21, 847–854. 10.1016/0270-9139(95)90540-5 7875683

[B96] Fuchs B. C. Hoshida Y. Fujii T. Wei L. Yamada S. Lauwers G. Y.. (2014). Epidermal growth factor receptor inhibition attenuates liver fibrosis and development of hepatocellular carcinoma. Hepatology 59, 1577–1590. 10.1002/hep.26898 24677197 PMC4086837

[B97] Gaça M. D. Zhou X. Benyon R. C. (2002). Regulation of hepatic stellate cell proliferation and collagen synthesis by proteinase-activated receptors. J. Hepatol. 36, 362–369. 10.1016/S0168-8278(01)00285-9 11867180

[B98] Galicia-Moreno M. Rodríguez-Rivera A. Reyes-Gordillo K. Segovia J. Shibayama M. Tsutsumi V.. (2009). N-acetylcysteine prevents carbon tetrachloride-induced liver cirrhosis: role of liver transforming growth factor-beta and oxidative stress. Eur. J. Gastroenterol. Hepatol. 21, 908–914. 10.1097/MEG.0b013e32831f1f3a 19398917

[B99] Gao R. P. Brigstock D. R. (2009). Connective tissue growth factor hammerhead ribozyme attenuates human hepatic stellate cell function. World J. Gastroenterol. 15, 3807–3813. 10.3748/wjg.15.3807 19673024 PMC2726461

[B100] García L. Hernández I. Sandoval A. Salazar A. Garcia J. Vera J.. (2002). Pirfenidone effectively reverses experimental liver fibrosis. J. Hepatol. 37, 797–805. 10.1016/S0168-8278(02)00272-6 12445421

[B101] García-Trevijano E. R. Iraburu M. J. Fontana L. Domínguez-Rosales J. A. Auster A. Covarrubias-Pinedo A.. (1999). Transforming growth factor beta1 induces the expression of alpha1(I) procollagen mRNA by a hydrogen peroxide-C/EBPbeta-dependent mechanism in rat hepatic stellate cells. Hepatology 29, 960–970. 10.1002/hep.510290346 10051504

[B102] Ge W. S. Wang Y. J. Wu J. X. Fan J. G. Chen Y. W. Zhu L. (2014). β-catenin is overexpressed in hepatic fibrosis and blockage of Wnt/β-catenin signaling inhibits hepatic stellate cell activation. Mol. Med. Rep. 9, 2145–2151. 10.3892/mmr.2014.2099 24691643 PMC4055486

[B103] Gellibert F. de Gouville A. C. Woolven J. Mathews N. Nguyen V. L. Bertho-Ruault C. (2006). Discovery of 4-{4-[3-(pyridin-2-yl)-1H-pyrazol-4-yl]pyridin-2-yl}-N-(tetrahydro-2H-pyran-4-yl)benzamide (GW788388): a potent, selective, and orally active transforming growth factor-beta type I receptor inhibitor. J. Med. Chem. 49, 2210–2221. 10.1021/jm0509905 16570917

[B104] George J. Tsutsumi M. (2007). siRNA-mediated knockdown of connective tissue growth factor prevents N-nitrosodimethylamine-induced hepatic fibrosis in rats. Gene Ther. 14, 790–803. 10.1038/sj.gt.3302929 17344905

[B105] Georges P. C. Hui J. J. Gombos Z. McCormick M. E. Wang A. Y. Uemura M.. (2007). Increased stiffness of the rat liver precedes matrix deposition: implications for fibrosis. Am. J. Physiol. Gastrointest. Liver. Physiol. 293, G1147–G1154. 10.1152/ajpgi.00032.2007 17932231

[B106] Godichaud S. Krisa S. Couronné B. Dubuisson L. Mérillon J. M. Desmoulière A.. (2000). Deactivation of cultured human liver myofibroblasts by trans-resveratrol, a grapevine-derived polyphenol. Hepatology 31, 922–931. 10.1053/he.2000.5848 10733549

[B107] Greenspoon N. Hershkoviz R. Alon R. Varon D. Shenkman B. Marx G.. (1993). Structural analysis of integrin recognition and the inhibition of integrin-mediated cell functions by novel nonpeptidic surrogates of the Arg-Gly-Asp sequence. Biochemistry 32, 1001–1008. 10.1021/bi00055a002 8093840

[B108] Gressner A. M. Krull N. Bachem M. G. (1994). Regulation of proteoglycan expression in fibrotic liver and cultured fat-storing cells. Pathol. Res. Pract. 190, 864–882. 10.1016/S0344-0338(11)80990-8 7899135

[B109] Gressner A. M. Weiskirchen R. Breitkopf K. Dooley S. (2002). Roles of TGF-β in hepatic fibrosis. Front. Biosci. 7, d793–d807. 10.2741/gressner 11897555

[B110] Gressner A. M. Weiskirchen R. (2006). Modern pathogenetic concepts of liver fibrosis suggest stellate cells and TGF-beta as major players and therapeutic targets. J. Cell. Mol. Med. 10, 76–99. 10.1111/j.1582-4934.2006.tb00292.x 16563223 PMC3933103

[B111] Gressner O. A. Lahme B. Demirci I. Gressner A. M. Weiskirchen R. (2007). Differential effects of TGF-beta on connective tissue growth factor (CTGF/CCN2) expression in hepatic stellate cells and hepatocytes. J. Hepatol. 47, 699–710. 10.1016/j.jhep.2007.05.015 17629588

[B112] Guo S. G. Zhang W. Jiang T. Dai M. Zhang L. F. Meng Y. C.. (2004). Influence of serum collected from rat perfused with compound Biejiaruangan drug on hepatic stellate cells. World J. Gastroenterol. 10, 1487–1494. 15133859 10.3748/wjg.v10.i10.1487 PMC4656290

[B113] Guo Y. Xiao L. Sun L. Liu F. (2012). Wnt/beta-catenin signaling: a promising new target for fibrosis diseases. Physiol. Res. 61, 337–346. 22670697 10.33549/physiolres.932289

[B114] Hagens W. I. Olinga P. Meijer D. K. Groothuis G. M. Beljaars L. Poelstra K. (2006). Gliotoxin non-selectively induces apoptosis in fibrotic and normal livers. Liver Int. 26, 232–239. 10.1111/j.1478-3231.2005.01212.x 16448462

[B115] Hammerich L. Tacke F. (2014). Interleukins in chronic liver disease: lessons learned from experimental mouse models. Clin. Exp. Gastroenterol. 7, 297–306. 10.2147/CEG.S43737 25214799 PMC4158890

[B116] Handy J. A. Fu P. P. Kumar P. Mells J. E. Sharma S. Saxena N. K.. (2011). Adiponectin inhibits leptin signalling via multiple mechanisms to exert protective effects against hepatic fibrosis. Biochem. J. 440, 385–395. 10.1042/BJ20102148 21846328 PMC3226855

[B117] Hantgan R. R. Paumi C. Rocco M. Weisel J. W. (1999). Effects of ligand-mimetic peptides Arg-Gly-Asp-X (X = Phe, Trp, Ser) on alphaIIbbeta3 integrin conformation and oligomerization. Biochemistry 38, 14461–14474. 10.1021/bi9907680 10545168

[B118] Hara Y. Yamashita T. Oishi N. Nio K. Hayashi T. Nomura Y.. (2015). TSU-68 ameliorates hepatocellular carcinoma growth by inhibiting microenvironmental platelet-derived growth factor signaling. Anticancer Res. 35, 1423–1431. 25750293

[B119] Harrison S. A. Torgerson S. Hayashi P. Ward J. Schenker S. (2003). Vitamin E and vitamin C treatment improves fibrosis in patients with nonalcoholic steatohepatitis. Am. J. Gastroenterol. 98, 2485–2490. 10.1111/j.1572-0241.2003.08699.x 14638353

[B120] Hemmann S. Graf J. Roderfeld M. Roeb E. (2007). Expression of MMPs and TIMPs in liver fibrosis—a systematic review with special emphasis on anti-fibrotic strategies. J. Hepatol. 46, 955–975. 10.1016/j.jhep.2007.02.003 17383048

[B121] Henderson N. C. Mackinnon A. C. Farnworth S. L. Poirier F. Russo F. P. Iredale J. P.. (2006). Galectin-3 regulates myofibroblast activation and hepatic fibrosis. Proc. Natl. Acad. Sci. U.S.A. 103, 5060–5065. 10.1073/pnas.0511167103 16549783 PMC1458794

[B122] Hennenberg M. Trebicka J. Kohistani Z. Stark C. Nischalke H. D. Krämer B.. (2011). Hepatic and HSC-specific sorafenib effects in rats with established secondary biliary cirrhosis. Lab. Invest. 91, 241–251. 10.1038/labinvest.2010.148 20921950

[B123] Heuberger H. Bauer R. Friedl F. Heubl G. Hummelsberger J. Nögel R.. (2010). Cultivation and breeding of Chinese medicinal plants in Germany. Planta Med. 76, 1956–1962. 10.1055/s-0030-1250528 21077027

[B124] Heymann F. Hamesch K. Weiskirchen R. Tacke F. (2015). The concanavalin A model of acute hepatitis in mice. Lab. Anim. 49, 12–20. 10.1177/0023677215572841 25835734

[B125] Hong F. Chou H. Fiel M. I. Friedman S. L. (2013). Antifibrotic activity of sorafenib in experimental hepatic fibrosis: refinement of inhibitory targets, dosing, and window of efficacy *in vivo*. Dig. Dis. Sci. 58, 257–264. 10.1007/s10620-012-2325-y 22918681 PMC3543488

[B126] Hong S. W. Jung K. H. Zheng H. M. Lee H. S. Suh J. K. Park I. S.. (2010). The protective effect of resveratrol on dimethylnitrosamine-induced liver fibrosis in rats. Arch. Pharm. Res. 33, 601–609. 10.1007/s12272-010-0415-y 20422370

[B127] Horwitz L. D. (2003). Bucillamine: a potent thiol donor with multiple clinical applications. Cardiovasc. Drug. Rev. 21, 77–90. 10.1111/j.1527-3466.2003.tb00107.x 12847560

[B128] Housset C. Rockey D. C. Bissell D. M. (1993). Endothelin receptors in rat liver: lipocytes as a contractile target for endothelin 1. Proc. Natl. Acad. Sci. U.S.A. 90, 9266–9270. 10.1073/pnas.90.20.9266 8415690 PMC47548

[B129] Hsiang C. Y. Lin L. J. Kao S. T. Lo H. Y. Chou S. T. Ho T. Y. (2015). Glycyrrhizin, silymarin, and ursodeoxycholic acid regulate a common hepatoprotective pathway in HepG2 cells. Phytomedicine 22, 768–777. 10.1016/j.phymed.2015.05.053 26141764

[B130] Hu Y. B. Li D. G. Lu H. M. (2007). Modified synthetic siRNA targeting tissue inhibitor of metalloproteinase-2 inhibits hepatic fibrogenesis in rats. J. Gene Med. 9, 217–229. 10.1002/jgm.1009 17351970

[B131] Huang C. K. Yu T. de la Monte S. M. Wands J. R. Derdak Z. Kim M. (2015). Restoration of Wnt/β-catenin signaling attenuates alcoholic liver disease progression in a rat model. J. Hepatol. 63, 191–198. 10.1016/j.jhep.2015.02.030 25724365 PMC4475483

[B132] Huang Q. Huang R. Zhang S. Lin J. Wei L. He M.. (2013). Protective effect of genistein isolated from Hydrocotyle sibthorpioides on hepatic injury and fibrosis induced by chronic alcohol in rats. Toxicol. Lett. 217, 102–110. 10.1016/j.toxlet.2012.12.014 23274713

[B133] Humbert M. Segal E. S. Kiely D. G. Carlsen J. Schwierin B. Hoeper M. M. (2007). Results of European post-marketing surveillance of bosentan in pulmonary hypertension. Eur. Respir. J. 30, 338–344. 10.1183/09031936.00138706 17504794

[B134] Hunter T. (2009). Tyrosine phosphorylation: thirty years and counting. Curr. Opin. Cell Biol. 21, 140–146. 10.1016/j.ceb.2009.01.028 19269802 PMC2670436

[B135] Ikeda H. Inao M. Fujiwara K. (1996). Inhibitory effect of tranilast on activation and transforming growth factor beta 1 expression in cultured rat stellate cells. Biochem. Biophys. Res. Commun. 227, 322–327. 10.1006/bbrc.1996.1508 8878516

[B136] Ikura Y. Iwasa Y. Ueda M. (2010). Valproic acid administration for hepatic fibrosis: a balance between antifibrotic efficacy and hepatotoxicity. Hepatology 51, 2227–2228. 10.1002/hep.23688 20513011

[B137] Inagaki Y. Higashi K. Kushida M. Hong Y. Y. Nakao S. Higashiyama R.. (2008). Hepatocyte growth factor suppresses profibrogenic signal transduction via nuclear export of Smad3 with galectin-7. Gastroenterology 134, 1180–1190. 10.1053/j.gastro.2008.01.014 18395096

[B138] Isaji M. Nakajoh M. Naito J. (1987). Selective inhibition of collagen accumulation by N-(3,4-dimethoxycinnamoyl)anthranilic acid (N-5′) in granulation tissue. Biochem. Pharmacol. 36, 469–474. 10.1016/0006-2952(87)90353-4 2435288

[B139] Ishikawa H. Takaki A. Tsuzaki R. Yasunaka T. Koike K. Shimomura Y.. (2014). L-carnitine prevents progression of non-alcoholic steatohepatitis in a mouse model with upregulation of mitochondrial pathway. PLoS ONE 9:e100627. 10.1371/journal.pone.0100627 24983359 PMC4077577

[B140] Iwamoto H. Sakai H. Kotoh K. Nakamuta M. Nawata H. (1999). Soluble Arg-Gly-Asp peptides reduce collagen accumulation in cultured rat hepatic stellate cells. Dig. Dis. Sci. 44, 1038–1045. 10.1023/A:1026633302985 10235616

[B141] Jackson B. C. Nebert D. W. Vasiliou V. (2010). Update of human and mouse matrix metalloproteinase families. Hum. Genomics 4, 194–201. 20368140 10.1186/1479-7364-4-3-194 PMC3525976

[B142] Jang M. Cai L. Udeani G. O. Slowing K. V. Thomas C. F. Beecher C. W.. (1997). Cancer chemopreventive activity of resveratrol, a natural product derived from grapes. Science 275, 218–220. 10.1126/science.275.5297.218 8985016

[B143] Jong C. J. Azuma J. Schaffer S. (2012). Mechanism underlying the antioxidant activity of taurine: prevention of mitochondrial oxidant production. Amino. Acids 42, 2223–2232. 10.1007/s00726-011-0962-7 21691752

[B144] Junnarkar S. P. Tapuria N. Mani A. Dijk S. Fuller B. Seifalian A. M.. (2010). Attenuation of warm ischemia-reperfusion injury in the liver by bucillamine through decreased neutrophil activation and Bax/Bcl-2 modulation. J. Gastroenterol. Hepatol. 25, 1891–1899. 10.1111/j.1440-1746.2010.06312.x 21092002

[B145] Kaimori A. Potter J. J. Choti M. Ding Z. Mezey E. Koteish A. A. (2010). Histone deacetylase inhibition suppresses the transforming growth factor beta1-induced epithelial-to-mesenchymal transition in hepatocytes. Hepatology 52, 1033–1045. 10.1002/hep.23765 20564330

[B146] Kandarkar S. V. Sawant S. S. Ingle A. D. Deshpande S. S. Maru G. B. (1998). Subchronic oral hepatotoxicity of turmeric in mice–histopathological and ultrastructural studies. Indian J. Exp. Biol. 36, 675–679. 9782784

[B147] Karsdal M. A. Manon-Jensen T. Genovese F. Kristensen J. H. Nielsen M. J. Sand J. M.. (2015). Novel insights into the function and dynamics of extracellular matrix in liver fibrosis. Am. J. Physiol. Gastrointest. Liver Physiol. 308, G807–G830. 10.1152/ajpgi.00447.2014 25767261 PMC4437019

[B148] Katayama K. Saito M. Kawaguchi T. Endo R. Sawara K. Nishiguchi S.. (2014). Effect of zinc on liver cirrhosis with hyperammonemia: a preliminary randomized, placebo-controlled double-blind trial. Nutrition 30, 1409–1414. 10.1016/j.nut.2014.04.018 25280421

[B149] Kawaguchi K. Sakaida I. Tsuchiya M. Omori K. Takami T. Okita K. (2004). Pioglitazone prevents hepatic steatosis, fibrosis, and enzyme-altered lesions in rat liver cirrhosis induced by a choline-deficient L-amino acid-defined diet. Biochem. Biophys. Res. Commun. 315, 187–195. 10.1016/j.bbrc.2004.01.038 15013444

[B150] Kaya-Dagistanli F. Tanriverdi G. Altinok A. Ozyazgan S. Ozturk M. (2013). The effects of alpha lipoic acid on liver cells damages and apoptosis induced by polyunsaturated fatty acids. Food Chem. Toxicol. 53, 84–93. 10.1016/j.fct.2012.11.026 23200892

[B151] Kendall T. J. Hennedige S. Aucott R. L. Hartland S. N. Vernon M. A. Benyon R. C.. (2009). p75 Neurotrophin receptor signaling regulates hepatic myofibroblast proliferation and apoptosis in recovery from rodent liver fibrosis. Hepatology 49, 901–910. 10.1016/j.fct.2012.11.026 19072833

[B152] Kershenobich D. Vargas F. Garcia-Tsao G. Perez Tamayo R. Gent M. Rojkind M. (1988). Colchicine in the treatment of cirrhosis of the liver. N. Engl. J. Med. 318, 1709–1713. 10.1056/NEJM198806303182602 3287167

[B153] Kim J. Y. Kim K. M. Nan J. X. Zhao Y. Z. Park P. H. Lee S. J.. (2003). Induction of apoptosis by tanshinone I via cytochrome c release in activated hepatic stellate cells. Pharmacol. Toxicol. 92, 195–200. 10.1034/j.1600-0773.2003.920410.x 12753423

[B154] Kim K. H. Chen C. C. Monzon R. I. Lau L. F. (2013). Matricellular protein CCN1 promotes regression of liver fibrosis through induction of cellular senescence in hepatic myofibroblasts. Mol. Cell Biol. 33, 2078–2090. 10.1128/MCB.00049-13 23508104 PMC3647960

[B155] Kim do Y. Chung S. I. Ro S. W. Paik Y. H. Lee J. I. Jung M. K.. (2013). Combined effects of an antioxidant and caspase inhibitor on the reversal of hepatic fibrosis in rats. Apoptosis 18, 1481–1491. 10.1007/s10495-013-0896-5 24045874

[B156] Kinoshita K. Limuro Y. Otogawa K. Saika S. Inagaki Y. Nakajima Y.. (2007). Adenovirus-mediated expression of BMP-7 suppresses the development of liver fibrosis in rats. Gut 56, 706–714. 10.1136/gut.2006.092460 17127702 PMC1942155

[B157] Klein S. Klösel J. Schierwagen R. Körner C. Granzow M. Huss S.. (2012). Atorvastatin inhibits proliferation and apoptosis, but induces senescence in hepatic myofibroblasts and thereby attenuates hepatic fibrosis in rats. Lab. Invest. 92, 1440–1450. 10.1038/labinvest.2012.106 22890553

[B158] Kloubert V. Rink L. (2015). Zinc as a micronutrient and its preventive role of oxidative damage in cells. Food Funct. 6, 3195–3204. 10.1039/C5FO00630A 26286461

[B159] Klyosov A. Zomer E. Platt D. (2012). DAVANAT® (GM-CT-01) and colon cancer: preclinical and clinical (phase I and II) studies. Glycobiol. Drug Design 89–130. 10.1021/bk-2012-1102.ch004

[B160] Kojima-Yuasa A. Umeda K. Ohkita T. Opare Kennedy D. Nishiguchi S. Matsui-Yuasa I. (2005). Role of reactive oxygen species in zinc deficiency-induced hepatic stellate cell activation. Free Radic. Biol. Med. 39, 631–640. 10.1016/j.freeradbiomed.2005.04.015 16085181

[B161] Kondou H. Mushiake S. Etani Y. Miyoshi Y. Michigami T. Ozono K. (2003). A blocking peptide for transforming growth factor-β1 activation prevents hepatic fibrosis *in vivo*. J. Hepatol. 39, 742–748. 10.1016/S0168-8278(03)00377-5 14568256

[B162] Kotoh K. Nakamuta M. Kohjima M. Fukushima M. Morizono S. Kobayashi N.. (2004). Arg-Gly-Asp (RGD) peptide ameliorates carbon tetrachloride-induced liver fibrosis via inhibition of collagen production and acceleration of collagenase activity. Int. J. Mol. Med. 14, 1049–1053. 10.3892/ijmm.14.6.1049 15547672

[B163] Kuo W. L. Yu M. C. Lee J. F. Tsai C. N. Chen T. C. Chen M. F. (2012). Imatinib mesylate improves liver regeneration and attenuates liver fibrogenesis in CCL4-treated mice. J. Gastrointest. Surg. 16, 361–369. 10.1007/s11605-011-1764-7 22068968

[B164] Lan T. Kisseleva T. Brenner D. A. (2015). Deficiency of NOX1 or NOX4 prevents liver inflammation and fibrosis in mice through inhibition of hepatic stellate cell activation. PLoS ONE 10:e0129743. 10.1371/journal.pone.0129743 26222337 PMC4519306

[B165] Lazaro R. Wu R. Lee S. Zhu N. L. Chen C. L. French S. W.. (2015). Osteopontin deficiency does not prevent but promotes alcoholic neutrophilic hepatitis in mice. Hepatology 61, 129–140. 10.1002/hep.27383 25132354 PMC4280361

[B166] Leclercq I. A. Sempoux C. Stärkel P. Horsmans Y. (2006). Limited therapeutic efficacy of pioglitazone on progression of hepatic fibrosis in rats. Gut. 55, 1020–1029. 10.1136/gut.2005.079194 16484506 PMC1856308

[B167] Lee K. C. Chan C. C. Yang Y. Y. Hsieh Y. C. Huang Y. H. Lin H. C. (2013). Aliskiren attenuates steatohepatitis and increases turnover of hepatic fat in mice fed with a methionine and choline deficient diet. PLoS ONE 8:e77817. 10.1371/journal.pone.0077817 24204981 PMC3804600

[B168] Lee M. H. Yoon S. Moon J. O. (2004a). The flavonoid naringenin inhibits dimethylnitrosamine-induced liver damage in rats. Biol. Pharm. Bull. 27, 72–76. 10.1248/bpb.27.72 14709902

[B169] Lee S. Kim S. Le H. D. Meisel J. Strijbosch R. A. Nose V.. (2008). Reduction of hepatocellular injury after common bile duct ligation using omega-3 fatty acids. J. Pediatr. Surg. 43, 2010–2015. 10.1016/j.jpedsurg.2008.05.030 18970933

[B170] Lee S. H. Seo G. S. Park Y. N. Yoo T. M. Sohn D. H. (2004b). Effects and regulation of osteopontin in rat hepatic stellate cells. Biochem. Pharmacol. 68, 2367–2378. 10.1016/j.bcp.2004.08.022 15548383

[B171] Lee T. Y. Chang H. H. Wang G. J. Chiu J. H. Yang Y. Y. Lin H. C. (2006). Water-soluble extract of Salvia miltiorrhiza ameliorates carbon tetrachloride-mediated hepatic apoptosis in rats. J. Pharm Pharmacol. 58, 659–665. 10.1211/jpp.58.5.0011 16640835

[B172] Lemoine M. Serfaty L. Cervera P. Capeau J. Ratziu V. (2014). Hepatic molecular effects of rosiglitazone in human non-alcoholic steatohepatitis suggest long-term pro-inflammatory damage. Hepatol. Res. 44, 1241–1247. 10.1111/hepr.12244 24118921

[B173] Leung T. M. Wang X. Kitamura N. Fiel M. I. Nieto N. (2013). Osteopontin delays resolution of liver fibrosis. Lab Invest. 93, 1082–1089. 10.1038/labinvest.2013.104 23999249

[B174] Li G. Li D. Xie Q. Shi Y. Jiang S. Jin Y. (2008a). RNA interfering connective tissue growth factor prevents rat hepatic stellate cell activation and extracellular matrix production. J. Gene Med. 10, 1039–1047. 10.1002/jgm.1223 18613219

[B175] Li G. Xie Q. Shi Y. Li D. Zhang M. Jiang S.. (2006). Inhibition of connective tissue growth factor by siRNA prevents liver fibrosis in rats. J. Gene Med. 8, 889–900. 10.1002/jgm.894 16652398

[B176] Li J. Li X. Xu W. Wang S. Hu Z. Zhang Q.. (2015a). Antifibrotic effects of luteolin on hepatic stellate cells and liver fibrosis by targeting AKT/mTOR/p70S6K and TGFβ/Smad signalling pathways. Liver Int. 35, 1222–1233. 10.1111/liv.12638 25040634

[B177] Li L. Zhao X. Y. Wang B. E. (2008b). Down-regulation of transforming growth factor beta 1/activin receptor-like kinase 1 pathway gene expression by herbal compound 861 is related to deactivation of LX-2 cells. World J. Gastroenterol. 14, 2894–2899. 10.3748/wjg.14.2894 18473417 PMC2710734

[B178] Li M. Wang X. F. Shi J. J. Li Y. P. Yang N. Zhai S.. (2015b). Caffeic acid phenethyl ester inhibits liver fibrosis in rats. World J. Gastroenterol. 21, 3893–3903. 10.3748/wjg.v21.i13.3893 25852274 PMC4385536

[B179] Li X. Wang X. Han C. Wang X. Xing G. Zhou L.. (2013). Astragaloside IV suppresses collagen production of activated hepatic stellate cells via oxidative stress-mediated p38 MAPK pathway. Free Radic. Biol. Med. 60, 168–176. 10.1016/j.freeradbiomed.2013.02.027 23459070

[B180] Lian N. Jiang Y. Zhang F. Jin H. Lu C. Wu X.. (2015). Curcumin regulates cell fate and metabolism by inhibiting hedgehog signaling in hepatic stellate cells. Lab. Invest. 95, 790–803. 10.1038/labinvest.2015.59 25938627

[B181] Lin H. J. Chen J. Y. Lin C. F. Kao S. T. Cheng J. C. Chen H. L.. (2011a). Hepatoprotective effects of Yi Guan Jian, an herbal medicine, in rats with dimethylnitrosamine-induced liver fibrosis. J. Ethnopharmacol. 134, 953–960. 10.1016/j.jep.2011.02.013 21333722

[B182] Lin H. J. Tseng C. P. Lin C. F. Liao M. H. Chen C. M. Kao S. T.. (2011b). A Chinese herbal decoction, modified Yi Guan Jian, induces apoptosis in hepatic stellate cells through an ROS-mediated mitochondrial/caspase pathway. Evid. Based Complement Alternat Med. 2011:459531. 10.1155/2011/459531 20976079 PMC2957151

[B183] Lin Y. L. Lee T. F. Huang Y. J. Huang Y. T. (2006). Antiproliferative effect of salvianolic acid A on rat hepatic stellate cells. J. Pharm. Pharmacol. 58, 933–939. 10.1211/jpp.58.7.0008 16805953

[B184] Liu C. Hu Y. Xu L. Liu C. Liu P. (2009a). Effect of Fuzheng Huayu formula and its actions against liver fibrosis. Chin. Med. 4:12. 10.1186/1749-8546-4-12 19558726 PMC2720970

[B185] Liu H. Wei W. Sun W. Y. Li X. (2009b). Protective effects of astragaloside IV on porcine-serum-induced hepatic fibrosis in rats and *in vitro* effects on hepatic stellate cells. J. Ethnopharmacol. 122, 502–508. 10.1016/j.jep.2009.01.035 19429320

[B186] Liu H. L. Chen Y. Cui G. H. Zhou J. F. (2005a). Curcumin, a potent anti-tumor reagent, is a novel histone deacetylase inhibitor regulating B-NHL cell line Raji proliferation. Acta Pharmacol. Sin. 26, 603–609. 10.1111/j.1745-7254.2005.00081.x 15842781

[B187] Liu L. Wei J. Huo X. Fang S. Yao D. Gao J.. (2012). The *Salvia miltiorrhiza* monomer IH764-3 induces apoptosis of hepatic stellate cells *in vivo* in a bile duct ligation-induced model of liver fibrosis. Mol. Med. Rep. 6, 1231–1238. 10.3892/mmr.2012.1076 22971838

[B188] Liu P. Hu Y. Y. Liu C. Xu L. M. Liu C. H. Sun K. W.. (2005b). Multicenter clinical study on Fuzhenghuayu capsule against liver fibrosis due to chronic hepatitis B. World J. Gastroenterol. 11, 2892–2899. 10.3748/wjg.v11.i19.2892 15902724 PMC4305655

[B189] Liu Q. Zhang Y. Lin Z. Shen H. Chen L. Hu L.. (2010). Danshen extract 15,16-dihydrotanshinone I functions as a potential modulator against metabolic syndrome through multi-target pathways. J. Steroid Biochem. Mol. Biol. 120, 155–163. 10.1016/j.jsbmb.2010.03.090 20380878

[B190] Liu X. Wang W. Hu H. Tang N. Zhang C. Liang W.. (2006). Smad3 specific inhibitor, naringenin, decreases the expression of extracellular matrix induced by TGF-beta1 in cultured rat hepatic stellate cells. Pharm. Res. 23, 82–89. 10.1007/s11095-005-9043-5 16341574

[B191] Lorena D. Darby I. A. Gadeau A. P. Leen L. L. Rittling S. Porto L. C.. (2006). Osteopontin expression in normal and fibrotic liver. altered liver healing in osteopontin-deficient mice. J. Hepatol. 44, 383–390. 10.1016/j.jhep.2005.07.024 16221502

[B192] Lu J. Chen B. Li S. Sun Q. (2014). Tryptase inhibitor APC 366 prevents hepatic fibrosis by inhibiting collagen synthesis induced by tryptase/protease-activated receptor 2 interactions in hepatic stellate cells. Int. Immunopharmacol. 20, 352–357. 10.1016/j.intimp.2014.04.001 24735816

[B193] Lytle K. A. Depner C. M. Wong C. P. Jump D. B. (2015). Docosahexaenoic acid attenuates Western diet induced hepatic fibrosis in *Ldlr*^−∕−^ mice by targeting the TGFβ-Smad3 pathway. J. Lipid Res. 56, 1936–1946. 10.1194/jlr.M061275 26315048 PMC4583081

[B194] Maguire J. J. Davenport A. P. (2015). Endothelin receptors and their antagonists. Semin. Nephrol. 35, 125–136. 10.1016/j.semnephrol.2015.02.002 25966344 PMC4437774

[B195] Majumder S. Piguet A. C. Dufour J. F. Chatterjee S. (2013). Study of the cellular mechanism of Sunitinib mediated inactivation of activated hepatic stellate cells and its implications in angiogenesis. Eur. J. Pharmacol. 705, 86–95. 10.1016/j.ejphar.2013.02.026 23454556

[B196] Manea S. A. Constantin A. Manda G. Sasson S. Manea A. (2015). Regulation of Nox enzymes expression in vascular pathophysiology: focusing on transcription factors and epigenetic mechanisms. Redox Biol. 5, 358–366. 10.1016/j.redox.2015.06.012 26133261 PMC4501559

[B197] Mannaerts I. Nuytten N. R. Rogiers V. Vanderkerken K. van Grunsven L. A. Geerts A. (2010). Chronic administration of valproic acid inhibits activation of mouse hepatic stellate cells *in vitro* and *in vivo*. Hepatology 51, 603–614. 10.1002/hep.23334 19957378

[B198] Marcolin E. San-Miguel B. Vallejo D. Tieppo J. Marroni N. González-Gallego J.. (2012). Quercetin treatment ameliorates inflammation and fibrosis in mice with nonalcoholic steatohepatitis. J. Nutr. 142, 1821–1828. 10.3945/jn.112.165274 22915297

[B199] Marra F. Tacke F. (2014). Roles for chemokines in liver disease. Gastroenterology 147, 577–594.e1. 10.1053/j.gastro.2014.06.043 25066692

[B200] Martin I. V. Borkham-Kamphorst E. Zok S. van Roeyen C. R. Eriksson U. Boor P. (2013). Platelet-derived growth factor (PDGF)-C neutralization reveals differential roles of PDGF receptors in liver and kidney fibrosis. Am. J. Pathol. 18, 2107–2117. 10.1016/j.ajpath.2012.09.006 23141925

[B201] Mayuren C. Reddy V. V. Priya S. V. Devi V. A. (2010). Protective effect of Livactine against CCl(4) and paracetamol induced hepatotoxicity in adult Wistar rats. N. Am. J. Med. Sci. 2, 491–495. 10.4297/najms.2010.2491 22558553 PMC3339113

[B202] Mazo D. F. de Oliveira M. G. Pereira I. V. Cogliati B. Stefano J. T. de Souza G. F.. (2013). S-nitroso-N-acetylcysteine attenuates liver fibrosis in experimental nonalcoholic steatohepatitis. Drug. Des. Devel. Ther. 7, 553–563. 10.3892/mmr.2014.2995 23843692 PMC3702228

[B203] Mazor D. Greenberg L. Shamir D. Meyerstein D. Meyerstein N. (2006). Antioxidant properties of bucillamine: possible mode of action. Biochem. Biophys. Res. Commun. 349, 1171–1175. 10.1016/j.bbrc.2006.08.155 16970913

[B204] Meira Martins L. A. Vieira M. Q. Ilha M. de Vasconcelos M. Biehl H. B. Lima D. B.. (2015). The interplay between apoptosis, mitophagy and mitochondrial biogenesis induced by resveratrol can determine activated hepatic stellate cells death or survival. Cell Biochem. Biophys. 71, 657–672. 10.1007/s12013-014-0245-5 25234614

[B205] Meurer S. K. Lahme B. Tihaa L. Weiskirchen R. Gressner A. M. (2005). N-acetyl-L-cysteine suppresses TGF-beta signaling at distinct molecular steps: the biochemical and biological efficacy of a multifunctional, antifibrotic drug. Biochem. Pharmacol. 70, 1026–1034. 10.1016/j.bcp.2005.07.001 16098950

[B206] Min A. K. Kim M. K. Seo H. Y. Kim H. S. Jang B. K. Hwang J. S.. (2010). α-lipoic acid inhibits hepatic PAI-1 expression and fibrosis by inhibiting the TGF-β signaling pathway. Biochem. Biophys. Res. Commun. 393, 536–541. 10.1016/j.bbrc.2010.02.050 20153726

[B207] Modi A. A. Feld J. J. Park Y. Kleiner D. E. Everhart J. E. Liang T. J.. (2010). Increased caffeine consumption is associated with reduced hepatic fibrosis. Hepatology 51, 201–209. 10.1002/hep.23279 20034049 PMC2801884

[B208] Monga S. P. (2015). β-Catenin signaling and roles in liver homeostasis, injury, and tumorigenesis. Gastroenterology 148, 1294–1310. 10.1053/j.gastro.2015.02.056 25747274 PMC4494085

[B209] Morell C. M. Strazzabosco M. (2014). Notch signaling and new therapeutic options in liver disease. J. Hepatol. 60, 885–890. 10.1016/j.jhep.2013.11.028 24308992

[B210] Moreno M. G. Chávez E. Aldaba-Muruato L. R. Segovia J. Vergara P. Tsutsumi V.. (2011). Coffee prevents CCl(4)-induced liver cirrhosis in the rat. Hepatol. Int. 5, 857–863. 10.1007/s12072-010-9247-6 21484136

[B211] Morgan T. R. Weiss D. G. Nemchausky B. Schiff E. R. Anand B. Simon F.. (2005). Colchicine treatment of alcoholic cirrhosis: a randomized, placebo-controlled clinical trial of patient survival. Gastroenterology 128, 882–890. 10.1053/j.gastro.2005.01.057 15825072

[B212] Munshi M. K. Uddin M. N. Glaser S. S. (2011). The role of the renin-angiotensin system in liver fibrosis. Exp. Biol. Med. (Maywood) 236, 557–566. 10.1258/ebm.2011.010375 21508249

[B213] Muntoni S. Rojkind M. Muntoni S. (2010). Colchicine reduces procollagen III and increases pseudocholinesterase in chronic liver disease. World J. Gastroenterol. 16, 2889–2894. 10.3748/wjg.v16.i23.2889 20556834 PMC2887584

[B214] Murad S. Pinnell S. R. (1987). Suppression of fibroblast proliferation and lysyl hydroxylase activity by minoxidil. J. Biol. Chem. 262, 11973–11978. 2442156

[B215] Nair D. G. Weiskirchen R. Al-Musharafi S. K. (2015). The use of marine-derived bioactive compounds as potential hepatoprotective agents. Acta Pharmacol. Sin. 36, 158–170. 10.1038/aps.2014.114 25500871 PMC4326787

[B216] Nakamura M. Bhatnagar A. Sadoshima J. (2012). Overview of pyridine nucleotides review series. Circ. Res. 111, 604–610. 10.1161/CIRCRESAHA.111.247924 22904040 PMC3523884

[B217] Nan Y. M. Fu N. Wu W. J. Liang B. L. Wang R. Q. Zhao S. X.. (2009). Rosiglitazone prevents nutritional fibrosis and steatohepatitis in mice. Scand. J. Gastroenterol. 44, 358–365. 10.1080/00365520802530861 18991162

[B218] Nature (2007). Hard to swallow. Nature 448, 105–106. 17625521 10.1038/448106a

[B219] Nava-Ocampo A. A. Suster S. Muriel P. (1997). Effect of colchiceine and ursodeoxycholic acid on hepatocyte and erythrocyte membranes and liver histology in experimentally induced carbon tetrachloride cirrhosis in rats. Eur. J. Clin. Invest. 27, 77–84. 10.1046/j.1365-2362.1997.910615.x 9041381

[B220] Neuschwander-Tetri B. A. Loomba R. Sanyal A. J. Lavine J. E. van Natta M. L. Abdelmalek M. F. (2015). NASH Clinical Research Network. Farnesoid X nuclear receptor ligand obeticholic acid for non-cirrhotic, non-alcoholic steatohepatitis (FLINT): a multicentre, randomised, placebo-controlled trial. Lancet 385, 956–965. 10.1016/S0140-6736(14)61933-4 25468160 PMC4447192

[B221] Niki T. Rombouts K. De Bleser P. De Smet K. Rogiers V. Schuppan D.. (1999). A histone deacetylase inhibitor, trichostatin A, suppresses myofibroblastic differentiation of rat hepatic stellate cells in primary culture. Hepatology 29, 858–867. 10.1002/hep.510290328 10051490

[B222] Nishimura N. Naora K. Hirano H. Iwamoto K. (2001). Changes in the dissolution of tolbutamide by a traditional Chinese medicine, Sho-saiko-to (Xiao Chaihu Tang). Biol. Pharm. Bull. 24, 409–413. 10.1248/bpb.24.409 11305604

[B223] Oakley F. Trim N. Constandinou C. M. Ye W. Gray A. M. Frantz G.. (2003). Hepatocytes express nerve growth factor during liver injury: evidence for paracrine regulation of hepatic stellate cell apoptosis. Am. J. Pathol. 163, 1849–1858. 10.1016/S0002-9440(10)63544-4 14578185 PMC1892444

[B224] Okada Y. Yamaguchi K. Nakajima T. Nishikawa T. Jo M. Mitsumoto Y.. (2013). Rosuvastatin ameliorates high-fat and high-cholesterol diet-induced nonalcoholic steatohepatitis in rats. Liver Int. 33, 301–311. 10.1111/liv.12033 23295058

[B225] Okuyama H. Nakamura H. Shimahara Y. Uyama N. Kwon Y. W. Kawada N.. (2005). Overexpression of thioredoxin prevents thioacetamide-induced hepatic fibrosis in mice. J. Hepatol. 42, 117–123. 10.1016/j.jhep.2004.09.020 15629516

[B226] Olteanu D. Nagy A. Dudea M. Filip A. Muresan A. Catoi C.. (2012). Hepatic and systemic effects of rosuvastatin on an experimental model of bile duct ligation in rats. J. Physiol. Pharmacol. 63, 483–496. 23211302

[B227] Orr J. G. Leel V. Cameron G. A. Marek C. J. Haughton E. L. Elrick L. J.. (2004). Mechanism of action of the antifibrogenic compound gliotoxin in rat liver cells. Hepatology 40, 232–242. 10.1002/hep.20254 15239107

[B228] Oseini A. M. Roberts L. R. (2009). PDGFRalpha: a new therapeutic target in the treatment of hepatocellular carcinoma? Expert. Opin. Ther. Targets 13, 443–454. 10.1517/14728220902719233 19335066

[B229] Padayatty S. J. Katz A. Wang Y. Eck P. Kwon O. Lee J. H.. (2003). Vitamin C as an antioxidant: evaluation of its role in disease prevention. J. Am. Coll. Nutr. 22, 18–35. 10.1080/07315724.2003.10719272 12569111

[B230] Paik Y. H. Yoon Y. J. Lee H. C. Jung M. K. Kang S. H. Chung S. I.. (2011). Antifibrotic effects of magnesium lithospermate B on hepatic stellate cells and thioacetamide-induced cirrhotic rats. Exp. Mol. Med. 43, 341–349. 10.3858/emm.2011.43.6.037 21499011 PMC3128912

[B231] Pan T. L. Wang P. W. (2012). Explore the molecular mechanism of apoptosis induced by tanshinone IIA on activated rat hepatic stellate cells. Evid. Based Complement Alternat. Med. 2012:734987. 10.1155/2012/734987 23346212 PMC3546466

[B232] Parajuli D. R. Zhao Y. Z. Jin H. Chi J. H. Li S. Y. Kim Y. C.. (2015). Anti-fibrotic effect of PF2401-SF, a standardized fraction of Salvia miltiorrhiza, in thioacetamide-induced experimental rats liver fibrosis. Arch. Pharm. Res. 38, 549–555. 10.1007/s12272-014-0425-2 25005065

[B233] Park J. H. Ryu S. H. Choi E. K. Ahn S. D. Park E. Choi K. C.. (2015a). SKI2162, an inhibitor of the TGF-β type I receptor (ALK5), inhibits radiation-induced fibrosis in mice. Oncotarget 6, 4171–4179. 10.18632/oncotarget.2878 25686821 PMC4414180

[B234] Park J. K. Ki M. R. Lee H. R. Hong I. H. Ji A. R. Ishigami A.. (2010). Vitamin C deficiency attenuates liver fibrosis by way of up-regulated peroxisome proliferator-activated receptor-γ expression in senescence marker protein 30 knockout mice. Hepatology 51, 1766–1777. 10.1002/hep.23499 20162732

[B235] Park S. A. Kim M. J. Park S. Y. Kim J. S. Lee S. J. Woo H. A.. (2015b). EW-7197 inhibits hepatic, renal, and pulmonary fibrosis by blocking TGF-β/Smad and ROS signaling. Cell. Mol. Life Sci. 72, 2023–2039. 10.1007/s00018-014-1798-6 25487606 PMC11113926

[B236] Patouraux S. Rousseau D. Rubio A. Bonnafous S. Lavallard V. J. Lauron J.. (2014). Osteopontin deficiency aggravates hepatic injury induced by ischemia-reperfusion in mice. Cell Death Dis. 5:e1208. 10.1038/cddis.2014.174 24810044 PMC4047890

[B237] Peng H. Carretero O. A. Raij L. Yang F. Kapke A. Rhaleb N. E. (2001). Antifibrotic effects of N-acetyl-seryl-aspartyl-Lysyl-proline on the heart and kidney in aldosterone-salt hypertensive rats. Hypertension 37, 794–800. 10.1161/01.HYP.37.2.794 11230375 PMC6824419

[B238] Peng J. Li X. Feng Q. Chen L. Xu L. Hu Y. (2013a). Anti-fibrotic effect of *Cordyceps sinensis* polysaccharide: Inhibiting HSC activation, TGF-β1/Smad signalling, MMPs and TIMPs. Exp. Biol. Med. (Maywood) 238, 668–677. 10.1177/1535370213480741 23918878

[B239] Peng Y. Yang H. Wang N. Ouyang Y. Yi Y. Liao L.. (2014). Fluorofenidone attenuates hepatic fibrosis by suppressing the proliferation and activation of hepatic stellate cells. Am. J. Physiol. Gastrointest. Liver Physiol. 306, G253–G263. 10.1152/ajpgi.00471.2012 24337009

[B240] Peng Y. Yang H. Zhu T. Zhao M. Deng Y. Liu B.. (2013b). The antihepatic fibrotic effects of fluorofenidone via MAPK signalling pathways. Eur. J. Clin. Invest. 43, 358–368. 10.1111/eci.12053 23438945

[B241] Pessayre D. Mansouri A. Haouzi D. Fromenty B. (1999). Hepatotoxicity due to mitochondrial dysfunction. Cell Biol. Toxicol. 15, 367–373. 10.1023/A:1007649815992 10811531

[B242] Pfalzer A. C. Choi S. W. Tammen S. A. Park L. K. Bottiglieri T. Parnell L. D.. (2014). S-adenosylmethionine mediates inhibition of inflammatory response and changes in DNA methylation in human macrophages. Physiol. Genomics 46, 617–623. 10.1152/physiolgenomics.00056.2014 25180283

[B243] Poo J. L. Feldmann G. Moreau A. Gaudin C. Lebrec D. (1993). Early colchicine administration reduces hepatic fibrosis and portal hypertension in rats with bile duct ligation. J. Hepatol. 19, 90–94. 10.1016/S0168-8278(05)80181-3 8301049

[B244] Poyrazoglu O. K. Bahcecioglu I. H. Ataseven H. Metin K. Dagli A. F. Yalniz M.. (2008). Effect of unfiltered coffee on carbon tetrachloride-induced liver injury in rats. Inflammation 31, 408–413. 10.1007/s10753-008-9092-0 19009339

[B245] Rafacho B. P. Stice C. P. Liu C. Greenberg A. S. Ausman L. M. Wang X. D. (2015). Inhibition of diethylnitrosamine-initiated alcohol-promoted hepatic inflammation and precancerous lesions by flavonoid luteolin is associated with increased sirtuin 1 activity in mice. Hepatobiliary Surg. Nutr. 4, 124–134. 10.3978/j.issn.2304-3881.2014.08.06 26005679 PMC4405419

[B246] Ramadoss S. Kannan K. Balamurugan K. Jeganathan N. S. Manavalan R. (2012). Evaluation of hepato-protective activity in the ethanolic extract of Sida rhombifolia Linn. against paracetamol-induced hepatic injury in albino rats. RJPBCS 3, 497–501.

[B247] Rockey D. C. Chung J. J. (1996). Endothelin antagonism in experimental hepatic fibrosis.Implications for endothelin in the pathogenesis of wound healing. J. Clin. Invest. 98, 1381–1388. 10.1172/JCI118925 8823303 PMC507564

[B248] Roderfeld M. Weiskirchen R. Wagner S. Berres M. L. Henkel C. Grötzinger J.. (2006). Inhibition of hepatic fibrogenesis by matrix metalloproteinase-9 mutants in mice. FASEB J. 20, 444–454. 10.1096/fj.05-4828com 16507762

[B249] Rodríguez-Pascual F. Busnadiego O. González-Santamaría J. (2014). The profibrotic role of endothelin-1: is the door still open for the treatment of fibrotic diseases? Life Sci. 118, 156–164. 10.1016/j.lfs.2013.12.024 24378671

[B250] Rojkind M. Kershenobich D. (1975). Effect of colchicine on collagen, albumin and transferrin synthesis by cirrhotic rat liver slices. Biochim. Biophys. Acta 378, 415–423. 10.1016/0005-2787(75)90186-0 1115789

[B251] Rombouts K. Kisanga E. Hellemans K. Wielant A. Schuppan D. Geerts A. (2003). Effect of HMG-CoA reductase inhibitors on proliferation and protein synthesis by rat hepatic stellate cells. J. Hepatol. 38, 564–572. 10.1016/S0168-8278(03)00051-5 12713866

[B252] Russell W. M. S. Burch R. L. (1959). The Principles of Humane Experimental Technique. London: Methuen & Co Ltd, 1959 *Reprinted by UFAW, Potters Bar, Herts, 1992* Available online at: http://altweb.jhsph.edu/pubs/books/humane_exp/het-toc

[B253] Ruwart M. J. Rush B. D. Snyder K. F. Peters K. M. Appelman H. D. Henley K. S. (1988). 16,16-Dimethyl prostaglandin E2 delays collagen formation in nutritional injury in rat liver. Hepatology 8, 61–64. 10.1002/hep.1840080112 3338720

[B254] Sakaida I. Matsumura Y. Akiyama S. Hayashi K. Ishige A. Okita K. (1998). Herbal medicine Sho-saiko-to (TJ-9) prevents liver fibrosis and enzyme-altered lesions in rat liver cirrhosis induced by a choline-deficient L-amino acid-defined diet. J. Hepatol. 28, 298–306. 10.1016/0168-8278(88)80017-5 9514543

[B255] Sakaida I. Uchida K. Hironaka K. Okita K. (1999). Prolyl 4-hydroxylase inhibitor (HOE 077) prevents TIMP-1 gene expression in rat liver fibrosis. J. Gastroenterol. 34, 376–377. 10.1007/s005350050277 10433015

[B256] Salazar-Montes A. Ruiz-Corro L. López-Reyes A. Castrejón-Gómez E. Armendáriz-Borunda J. (2008). Potent antioxidant role of pirfenidone in experimental cirrhosis. Eur. J. Pharmacol. 595, 69–77. 10.1016/j.ejphar.2008.06.110 18652820

[B257] Sánchez-Valle V. Chávez-Tapia N. C. Uribe M. Méndez-Sánchez N. (2012). Role of oxidative stress and molecular changes in liver fibrosis: a review. Curr. Med. Chem. 19, 4850–4860. 10.2174/092986712803341520 22709007

[B258] Sanderson N. Factor V. Nagy P. Kopp J. Kondaiah P. Wakefield L.. (1995). Hepatic expression of mature transforming growth factor beta 1 in transgenic mice results in multiple tissue lesions. Proc. Natl. Acad. Sci. U.S.A. 92, 2572–2576. 10.1073/pnas.92.7.2572 7708687 PMC42260

[B259] San-Miguel B. Crespo I. Sánchez D. I. González-Fernández B. Ortiz de Urbina J. J. Tuñón M. J.. (2015). Melatonin inhibits autophagy and endoplasmic reticulum stress in mice with carbon tetrachloride-induced fibrosis. J. Pineal. Res. 59, 151–162. 10.1111/jpi.12247 25958928

[B260] Sapakal V. D. Ghadge R. V. Adnaik R. S. Naikwade N. S. Magdum C. S. (2008). Comparative hepatoprotective activity of Liv-52 and livomyn against carbon tetrachloride-induced hepatic injury in rats. Int. J. Green Pharm. 2, 79–82. 10.4103/0973-8258.41175

[B261] Sayin S. I. Wahlström A. Felin J. Jäntti S. Marschall H. U. Bamberg K.. (2013). Gut microbiota regulates bile acid metabolism by reducing the levels of tauro-beta-muricholic acid, a naturally occurring FXR antagonist. Cell Metab. 17, 225–235. 10.1016/j.cmet.2013.01.003 23395169

[B262] Schaap F. G. Trauner M. Jansen P. L. (2014). Bile acid receptors as targets for drug development. Nat. Rev. Gastroenterol. Hepatol. 11, 55–67. 10.1038/nrgastro.2013.151 23982684

[B263] Schaefer C. J. Ruhrmund D. W. Pan L. Seiwert S. D. Kossen K. (2011). Antifibrotic activities of pirfenidone in animal models. Eur. Respir. Rev. 20, 85–97. 10.1183/09059180.00001111 21632796 PMC9487788

[B264] Schmöcker C. Weylandt K. H. Kahlke L. Wang J. Lobeck H. Tiegs G.. (2007). Omega-3 fatty acids alleviate chemically induced acute hepatitis by suppression of cytokines. Hepatology 45, 864–869. 10.1002/hep.21626 17393517

[B265] Schultz-Cherry S. Murphy-Ullrich J. E. (1993). Thrombospondin causes activation of latent transforming growth factor-beta secreted by endothelial cells by a novel mechanism. J. Cell Biol. 122, 923–932. 10.1083/jcb.122.4.923 8349738 PMC2119591

[B266] Semwal R. B. Semwal D. K. Combrinck S. Viljoen A. M. (2015). Gingerols and shogaols: important nutraceutical principles from ginger. Phytochemistry 117, 554–568. 10.1016/j.phytochem.2015.07.012 26228533

[B267] Serviddio G. Bellanti F. Stanca E. Lunetti P. Blonda M. Tamborra R.. (2014). Silybin exerts antioxidant effects and induces mitochondrial biogenesis in liver of rat with secondary biliary cirrhosis. Free Radic. Biol. Med. 73, 117–126. 10.1016/j.freeradbiomed.2014.05.002 24819445

[B268] Shaaban A. A. Shaker M. E. Zalata K. R. El-kashef H. A. Ibrahim T. M. (2014). Modulation of carbon tetrachloride-induced hepatic oxidative stress, injury and fibrosis by olmesartan and omega-3. Chem. Biol. Interact. 207, 81–91. 10.1016/j.cbi.2013.10.008 24144775

[B269] Shaker M. E. Salem H. A. Shiha G. E. Ibrahim T. M. (2011a). Nilotinib counteracts thioacetamide-induced hepatic oxidative stress and attenuates liver fibrosis progression. Fundam. Clin. Pharmacol. 25, 248–257. 10.1111/j.1472-8206.2010.00824.x 20408881

[B270] Shaker M. E. Zalata K. R. Mehal W. Z. Shiha G. E. Ibrahim T. M. (2011b). Comparison of imatinib, nilotinib and silymarin in the treatment of carbon tetrachloride-induced hepatic oxidative stress, injury and fibrosis. Toxicol. Appl. Pharmacol. 252, 165–175. 10.1016/j.taap.2011.02.004 21316382 PMC3895503

[B271] Shen K. Feng X. Su R. Xie H. Zhou L. Zheng S. (2015). Epigallocatechin 3-gallate ameliorates bile duct ligation induced liver injury in mice by modulation of mitochondrial oxidative stress and inflammation. PLoS ONE 10:e0126278. 10.1371/journal.pone.0126278 25955525 PMC4425400

[B272] Shi F. Sheng Q. Xu X. Huang W. Kang Y. J. (2015). Zinc supplementation suppresses the progression of bile duct ligation-induced liver fibrosis in mice. Exp. Biol. Med. (Maywood) 240, 1197–1204. 10.1177/1535370214558026 25432983 PMC4935358

[B273] Shiha G. E. Abu-Elsaad N. M. Zalata K. R. Ibrahim T. M. (2014). Tracking anti-fibrotic pathways of nilotinib and imatinib in experimentally induced liver fibrosis: an insight. Clin. Exp. Pharmacol. Physiol. 41, 788–797. 10.1111/1440-1681.12286 25115651

[B274] Shim S. G. Jun D. W. Kim E. K. Saeed W. K. Lee K. N. Lee H. L.. (2013). Caffeine attenuates liver fibrosis via defective adhesion of hepatic stellate cells in cirrhotic model. J. Gastroenterol. Hepatol. 28, 1877–1884. 10.1111/jgh.12317 23808892

[B275] Shimizu I. Ma Y. R. Mizobuchi Y. Liu F. Miura T. Nakai Y.. (1999). Effects of Sho-saiko-to, a Japanese herbal medicine, on hepatic fibrosis in rats. Hepatology 29, 149–160. 10.1002/hep.510290108 9862861

[B276] Shinkawa H. Takemura S. Minamiyama Y. Kodai S. Tsukioka T. Osada-Oka M.. (2009). S-allylcysteine is effective as a chemopreventive agent against porcine serum-induced hepatic fibrosis in rats. Osaka City Med. J. 55, 61–69. 20088405

[B277] Shirin H. Sharvit E. Aeed H. Gavish D. Bruck R. (2013). Atorvastatin and rosuvastatin do not prevent thioacetamide induced liver cirrhosis in rats. World J. Gastroenterol. 19, 241–248. 10.3748/wjg.v19.i2.241 23345947 PMC3547559

[B278] Stefano J. T. Cogliati B. Santos F. Lima V. M. Mazo D. C. Matte U.. (2011). S-Nitroso-N-acetylcysteine induces de-differentiation of activated hepatic stellate cells and promotes antifibrotic effects *in vitro*. Nitric Oxide 25, 360–365. 10.1016/j.niox.2011.07.001 21820071

[B279] Stevens J. F. Page J. E. (2004). Xanthohumol and related prenylflavonoids from hops and beer: to your good health! Phytochemistry 65, 1317–1330. 10.1016/j.phytochem.2004.04.025 15231405

[B280] Su T. H. Shiau C. W. Jao P. Liu C. H. Liu C. J. Tai W. T.. (2015). Sorafenib and its derivative SC-1 exhibit antifibrotic effects through signal transducer and activator of transcription 3 inhibition. Proc. Natl. Acad. Sci. U.S.A. 112, 7243–7248. 10.1073/pnas.1507499112 26039995 PMC4466718

[B281] Surapaneni K. M. Jainu M. (2014). Pioglitazone, quercetin and hydroxy citric acid effect on hepatic biomarkers in Non Alcoholic Steatohepatitis. Pharmacognosy Res. 6, 153–162. 10.4103/0974-8490.129037 24761121 PMC3996753

[B282] Susnea I. Weiskirchen R. (2015). Trace metal imaging in diagnostic of hepatic metal disease. Mass Spectrom. Rev. [Epub ahead of print]. 10.1002/mas.21454 25677057

[B283] Suzuki Y. Katagiri F. Sato F. Fujioka K. Sato Y. Fujioka T.. (2014). Significant decrease in plasma N-acetyl-seryl-aspartyl-lysyl-proline level in patients with end stage renal disease after kidney transplantation. Biol. Pharm. Bull. 37, 1075–1079. 10.1248/bpb.b13-00992 24882420

[B284] Tada S. Nakamuta M. Enjoji M. Sugimoto R. Iwamoto H. Kato M.. (2001). Pirfenidone inhibits dimethylnitrosamine-induced hepatic fibrosis in rats. Clin. Exp. Pharmacol. Physiol. 28, 522–527. 10.1046/j.1440-1681.2001.03481.x 11422218

[B285] Thabut D. Routray C. Lomberk G. Shergill U. Glaser K. Huebert R.. (2011). Complementary vascular and matrix regulatory pathways underlie the beneficial mechanism of action of sorafenib in liver fibrosis. Hepatology 54, 573–585. 10.1002/hep.24427 21567441 PMC3145033

[B286] Tahan V. Ozaras R. Canbakan B. Uzun H. Aydin S. Yildirim B.. (2004). Melatonin reduces dimethylnitrosamine-induced liver fibrosis in rats. J. Pineal. Res. 37, 78–84. 10.1111/j.1600-079X.2004.00137.x 15298665

[B287] Takahashi Y. Soejima Y. Kumagai A. Watanabe M. Uozaki H. Fukusato T. (2014). Inhibitory effects of Japanese herbal medicines sho-saiko-to and juzen-taiho-to on nonalcoholic steatohepatitis in mice. PLoS ONE 9:e87279. 10.1371/journal.pone.0087279 24466347 PMC3899375

[B288] Takase S. Matsuda Y. Yasuhara M. Takada A. (1989). Effects of malotilate treatment on alcoholic liver disease. Alcohol 6, 219–222. 10.1016/0741-8329(89)90021-9 2544209

[B289] Tang Y. (2015). Curcumin targets multiple pathways to halt hepatic stellate cell activation: updated mechanisms *in vitro* and *in vivo*. Dig. Dis. Sci. 60, 1554–1564. 10.1007/s10620-014-3487-6 25532502

[B290] Tipoe G. L. Leung T. M. Liong E. C. Lau T. Y. Fung M. L. Nanji A. A. (2010). Epigallocatechin-3-gallate (EGCG) reduces liver inflammation, oxidative stress and fibrosis in carbon tetrachloride (CCl4)-induced liver injury in mice. Toxicology 273, 45–52. 10.1016/j.tox.2010.04.014 20438794

[B291] Traber P. G. Chou H. Zomer E. Hong F. Klyosov A. Fiel M. I.. (2013). Regression of fibrosis and reversal of cirrhosis in rats by galectin inhibitors in thioacetamide-induced liver disease. PLoS ONE 8:e75361. 10.1371/journal.pone.0075361 24130706 PMC3793988

[B292] Trebicka J. Hennenberg M. Odenthal M. Shir K. Klein S. Granzow M.. (2010). Atorvastatin attenuates hepatic fibrosis in rats after bile duct ligation via decreased turnover of hepatic stellate cells. J. Hepatol. 53, 702–712. 10.1016/j.jhep.2010.04.025 20633948

[B293] Tsai J. H. Liu J. Y. Wu T. T. Ho P. C. Huang C. Y. Shyu J. C.. (2008). Effects of silymarin on the resolution of liver fibrosis induced by carbon tetrachloride in rats. J. Viral Hepat. 15, 508–514. 10.1111/j.1365-2893.2008.00971.x 18397225

[B294] Tsalkidou E. G. Tsaroucha A. K. Chatzaki E. Lambropoulou M. Papachristou F. Trypsianis G.. (2014). The effects of apigenin on the expression of Fas/FasL apoptotic pathway in warm liver ischemia-reperfusion injury in rats. Biomed. Res. Int. 2014:157216. 10.1155/2014/157216 25110657 PMC4109422

[B295] Tugues S. Fernandez-Varo G. Muñoz-Luque J. Ros J. Arroyo V. Rodés J.. (2007). Antiangiogenic treatment with sunitinib ameliorates inflammatory infiltrate, fibrosis, and portal pressure in cirrhotic rats. Hepatology 46, 1919–1926. 10.1002/hep.21921 17935226

[B296] Tzeng J. I. Chen M. F. Chung H. H. Cheng J. T. (2013). Silymarin decreases connective tissue growth factor to improve liver fibrosis in rats treated with carbon tetrachloride. Phytother Res. 27, 1023–1028. 10.1002/ptr.4829 22933420

[B297] Uchio K. Graham M. Dean N. M. Rosenbaum J. Desmoulière A. (2004). Down-regulation of connective tissue growth factor and type I collagen mRNA expression by connective tissue growth factor antisense oligonucleotide during experimental liver fibrosis. Wound Repair Regen. 12, 60–66. 10.1111/j.1067-1927.2004.012112.x-1 14974966

[B298] Unger T. Chung O. Csikos T. Culman J. Gallinat S. Gohlke P.. (1996). Angiotensin receptors. J. Hypertens. Suppl. 14, S95–S103. 9120691

[B299] Urtasun R. Lopategi A. George J. Leung T. M. Lu Y. Wang X.. (2012). Osteopontin, an oxidant stress sensitive cytokine, up-regulates collagen-I via integrin α(V)β(3) engagement and PI3K/pAkt/NFκB signaling. Hepatology 55, 594–608. 10.1002/hep.24701 21953216 PMC3561739

[B300] Visse R. Nagase H. (2003). Matrix metalloproteinases and tissue inhibitors of metalloproteinases: structure, function, and biochemistry. Circ. Res. 92, 827–839. 10.1161/01.RES.0000070112.80711.3D 12730128

[B301] Wang B. E. (2000). Treatment of chronic liver diseases with traditional Chinese medicine. J. Gastroenterol. Hepatol. 15, E67–E70. 10.1046/j.1440-1746.2000.02100.x 10921385

[B302] Wang E. Chen F. Hu X. Yuan Y. (2014). Protective effects of apigenin against furan-induced toxicity in mice. Food Funct. 5, 1804–1812. 10.1039/C4FO00038B 24914499

[B303] Wang J. Leclercq I. Brymora J. M. Xu N. Ramezani-Moghadam M. London R. M.. (2009). Kupffer cells mediate leptin-induced liver fibrosis. Gastroenterology 137, 713–723. 10.1053/j.gastro.2009.04.011 19375424 PMC2757122

[B304] Wang L. Wang B. E. Wang J. Xiao P. G. Tan X. H. (2008). Herbal compound 861 regulates mRNA expression of collagen synthesis- and degradation-related genes in human hepatic stellate cells. World J. Gastroenterol. 14, 1790–1794. 10.3748/wjg.14.1790 18350612 PMC2695921

[B305] Wang L. Wang J. Wang B. E. Xiao P. G. Qiao Y. J. Tan X. H. (2004). Effects of herbal compound 861 on human hepatic stellate cell proliferation and activation. World J. Gastroenterol. 10, 2831–2835. 10.3748/wjg.v10.i19.2831 15334680 PMC4572112

[B306] Wang R. Yu X. Y. Guo Z. Y. Wang Y. J. Wu Y. Yuan Y. F. (2012). Inhibitory effects of salvianolic acid B on CCl(4)-induced hepatic fibrosis through regulating NF-κB/IκBα signaling. J. Ethnopharmacol. 144, 592–598. 10.1016/j.jep.2012.09.048 23041223

[B307] Wang W. Zhao C. Zhou J. Zhen Z. Wang Y. Shen C. (2013). Simvastatin ameliorates liver fibrosis via mediating nitric oxide synthase in rats with non-alcoholic steatohepatitis-related liver fibrosis. PLoS ONE 8:e76538. 10.1371/journal.pone.0076538 24098525 PMC3788732

[B308] Wang Y. Gao J. Zhang D. Zhang J. Ma J. Jiang H. (2010). New insights into the antifibrotic effects of sorafenib on hepatic stellate cells and liver fibrosis. J. Hepatol. 53, 132–144. 10.1016/j.jhep.2010.02.027 20447716

[B309] Wang Y. J. Wang S. S. Bickel M. Guenzler V. Gerl M. Bissell D. M. (1998). Two novel antifibrotics, HOE 077 and Safironil, modulate stellate cell activation in rat liver injury: differential effects in males and females. Am. J. Pathol. 152, 279–287. 9422545 PMC1858123

[B310] Wasser S. Ho J. M. Ang H. K. Tan C. E. (1998). Salvia miltiorrhiza reduces experimentally-induced hepatic fibrosis in rats. J. Hepatol. 29, 760–771. 10.1016/S0168-8278(98)80257-2 9833914

[B311] Weiskirchen R. (2011). CCN proteins in normal and injured liver. Front. Biosci. (Landmark Ed). 16, 1939–1961. 10.2741/3832 21196275

[B312] Weiskirchen R. Mahli A. Weiskirchen S. Hellerbrand C. (2015). The hop constituent xanthohumol exhibits hepatoprotective effects and inhibits the activation of hepatic stellate cells at different levels. Front. Physiol. 6:140. 10.3389/fphys.2015.00140 25999863 PMC4422013

[B313] Wen J. (2007). Sho-saiko-to, a clinically documented herbal preparation for treating chronic liver disease. HerbalGram 73, 34–43.

[B314] Wong C. C. Tse A. P. Huang Y. P. Zhu Y. T. Chiu D. K. Lai R. K.. (2014). Lysyl oxidase-like 2 is critical to tumor microenvironment and metastatic niche formation in hepatocellular carcinoma. Hepatology 60, 1645–1658. 10.1002/hep.27320 25048396

[B315] Wright M. C. Issa R. Smart D. E. Trim N. Murray G. I. Primrose J. N.. (2001). Gliotoxin stimulates the apoptosis of human and rat hepatic stellate cells and enhances the resolution of liver fibrosis in rats. Gastroenterology 121, 685–698. 10.1053/gast.2001.27188 11522753

[B316] Xia J. L. Dai C. Michalopoulos G. K. Liu Y. (2006). Hepatocyte growth factor attenuates liver fibrosis induced by bile duct ligation. Am. J. Pathol. 168, 1500–1512. 10.2353/ajpath.2006.050747 16651617 PMC1606599

[B317] Yamada H. Tajima S. Nishikawa T. Murad S. Pinnell S. R. (1994). Tranilast, a selective inhibitor of collagen synthesis in human skin fibroblasts. J. Biochem. 116, 892–897. 7533764 10.1093/oxfordjournals.jbchem.a124612

[B318] Yamasaki K. Takemura S. Minamiyama Y. Hai S. Yamamoto S. Kodai S.. (2005). Minoxidil, a K(ATP) channel opener, accelerates DNA synthesis following partial hepatectomy in rats. Biofactors 23, 15–23. 10.1002/biof.5520230103 15817995

[B319] Yang J. J. Tao H. Li J. (2014a). Hedgehog signaling pathway as key player in liver fibrosis: new insights and perspectives. Expert Opin. Ther. Targets 18, 1011–1021. 10.1517/14728222.2014.927443 24935558

[B320] Yang J. Hou Y. Ji G. Song Z. Liu Y. Dai G.. (2014b). Targeted delivery of the RGD-labeled biodegradable polymersomes loaded with the hydrophilic drug oxymatrine on cultured hepatic stellate cells and liver fibrosis in rats. Eur. J. Pharm. Sci. 52, 180–190. 10.1016/j.ejps.2013.11.017 24296297

[B321] Yang L. Kwon J. Popov Y. Gajdos G. B. Ordog T. Brekken R. A.. (2014c). Vascular endothelial growth factor promotes fibrosis resolution and repair in mice. Gastroenterology 146, 1339–1350.e1. 10.1053/j.gastro.2014.01.061 24503129 PMC4001704

[B322] Yang L. Qu M. Wang Y. Duan H. Chen P. Wang Y.. (2013). Trichostatin A inhibits transforming growth factor-β-induced reactive oxygen species accumulation and myofibroblast differentiation via enhanced NF-E2-related factor 2-antioxidant response element signaling. Mol. Pharmacol. 83, 671–680. 10.1124/mol.112.081059 23284002

[B323] Yin M. F. Lian L. H. Piao D. M. Nan J. X. (2007). Tetrandrine stimulates the apoptosis of hepatic stellate cells and ameliorates development of fibrosis in a thioacetamide rat model. World J. Gastroenterol. 13, 1214–1220. 10.3748/wjg.v13.i8.1214 17451202 PMC4146996

[B324] Yin S. S. Wang B. E. Wang T. L. Jia J. D. Qian L. X. (2004). [The effect of Cpd 861 on chronic hepatitis B related fibrosis and early cirrhosis: a randomized, double blind, placebo controlled clinical trial]. Zhonghua Gan Zang Bing Za Zhi. 12, 467–470. 15329205

[B325] Yoo N. Y. Jeon S. Nam Y. Park Y. J. Won S. B. Kwon Y. H. (2015). Dietary supplementation of genistein alleviates liver inflammation and fibrosis mediated by a methionine-choline-deficient diet in db/db mice. J. Agric. Food Chem. 63, 4305–4311. 10.1021/acs.jafc.5b00398 25885479

[B326] Yoshiji H. Kuriyama S. Yoshii J. Ikenaka Y. Noguchi R. Nakatani T.. (2001). Angiotensin-II type 1 receptor interaction is a major regulator for liver fibrosis development in rats. Hepatology 34, 745–750. 10.1053/jhep.2001.28231 11584371

[B327] Younes M. Siegers C. P. (1985). Effect of malotilate on paracetamol-induced hepatotoxicity. Toxicol. Lett. 25, 143–146. 10.1016/0378-4274(85)90074-8 4002245

[B328] Yu C. Wang F. Jin C. Huang X. Miller D. L. Basilico C.. (2003). Role of fibroblast growth factor type 1 and 2 in carbon tetrachloride-induced hepatic injury and fibrogenesis. Am. J. Pathol. 163, 1653–1662. 10.1016/S0002-9440(10)63522-5 14507672 PMC1868310

[B329] Yu C. Wang F. Jin C. Wu X. Chan W. K. McKeehan W. L. (2002). Increased carbon tetrachloride-induced liver injury and fibrosis in FGFR4-deficient mice. Am. J. Pathol. 161, 2003–2010. 10.1016/S0002-9440(10)64478-1 12466116 PMC1850898

[B330] Yu D. K. Zhang C. X. Zhao S. S. Zhang S. H. Zhang H. Cai S. Y.. (2015). The anti-fibrotic effects of epigallocatechin-3-gallate in bile duct-ligated cholestatic rats and human hepatic stellate LX-2 cells are mediated by the PI3K/Akt/Smad pathway. Acta Pharmacol. Sin. 36, 473–482. 10.1038/aps.2014.155 25832428 PMC4387300

[B331] Yu V. W. Ho W. S. (2013). Tetrandrine inhibits hepatocellular carcinoma cell growth through the caspase pathway and G2/M phase. Oncol Rep. 29, 2205–2210. 10.3892/or.2013.2352 23525490

[B332] Yuhua Z. Wanhua R. Chenggang S. Jun S. Yanjun W. Chunqing Z. (2008). Disruption of connective tissue growth factor by short hairpin RNA inhibits collagen synthesis and extracellular matrix secretion in hepatic stellate cells. Liver Int. 28, 632–639. 10.1111/j.1478-3231.2008.01730.x 18433392

[B333] Zhang F. Zhuge Y. Z. Li Y. J. Gu J. X. (2014). S-adenosylmethionine inhibits the activated phenotype of human hepatic stellate cells via Rac1 and matrix metalloproteinases. Int. Immunopharmacol. 19, 193–200. 10.1016/j.intimp.2014.01.021 24495518

[B334] Zhang J. Gao Y. Qian S. Liu X. Zu H. (2011a). Physicochemical and pharmacokinetic characterization of a spray-dried malotilate emulsion. Int. J. Pharm. 414, 186–192. 10.1016/j.ijpharm.2011.05.032 21619915

[B335] Zhang J. Liu X. Lei X. Wang L. Guo L. Zhao G.. (2010). Discovery and synthesis of novel luteolin derivatives as DAT agonists. Bioorg. Med. Chem. 18, 7842–7848. 10.1016/j.bmc.2010.09.049 20971650

[B336] Zhang J. J. Wang Y. L. Feng X. B. Song X. D. Liu W. B. (2011b). Rosmarinic acid inhibits proliferation and induces apoptosis of hepatic stellate cells. Biol. Pharm. Bull. 34, 343–348. 10.1248/bpb.34.343 21372382

[B337] Zhang L. Schuppan D. (2014). Traditional Chinese Medicine (TCM) for fibrotic liver disease: hope and hype. J. Hepatol. 61, 166–168. 10.1016/j.jhep.2014.03.009 24780816

[B338] Zhang L. Xu L. M. Chen Y. W. Ni Q. W. Zhou M. Qu C. Y.. (2012a). Antifibrotic effect of N-acetyl-seryl-aspartyl-lysyl-proline on bile duct ligation induced liver fibrosis in rats. World J. Gastroenterol. 18, 5283–5288. 10.3748/wjg.v18.i37.5283 23066324 PMC3468862

[B339] Zhang M. Song G. Minuk G. Y. (1996). Effects of hepatic stimulator substance, herbal medicine, selenium/vitamin E, and ciprofloxacin on cirrhosis in the rat. Gastroenterology 110, 1150–1155. 10.1053/gast.1996.v110.pm8613004 8613004

[B340] Zhang W. Wu R. Zhang F. Xu Y. Liu B. Yang Y.. (2012b). Thiazolidinediones improve hepatic fibrosis in rats with non-alcoholic steatohepatitis by activating the adenosine monophosphate-activated protein kinase signalling pathway. Clin. Exp. Pharmacol. Physiol. 39, 1026–1033. 10.1111/1440-1681.12020 23127227

[B341] Zhang W. J. Frei B. (2015). Astragaloside IV inhibits NF- κ B activation and inflammatory gene expression in LPS-treated mice. Mediators Inflamm. 2015:274314. 10.1155/2015/274314 25960613 PMC4415625

[B342] Zou W. L. Yang Z. Zang Y. J. Li D. J. Liang Z. P. Shen Z. Y. (2007). Inhibitory effects of prostaglandin E1 on activation of hepatic stellate cells in rabbits with schistosomiasis. Hepatobiliary Pancreat. Dis. Int. 6, 176–181. 17374578

[B343] Zuurmond A. M. van der Slot-Verhoeven A. J. van Dura E. A. De Groot J. Bank R. A. (2005). Minoxidil exerts different inhibitory effects on gene expression of lysyl hydroxylase 1, 2, and 3: implications for collagen cross-linking and treatment of fibrosis. Matrix Biol. 24, 261–270. 10.1016/j.matbio.2005.04.002 15908192

