# Supplementary figures and images for: Hepatoprotective and Anti-fibrotic Agents: It's Time to Take the Next Step
Source: Front Pharmacol. 2016 Jan 7;6:303. doi: 10.3389/fphar.2015.00303 (PMC4703795; doi:10.3389/fphar.2015.00303)

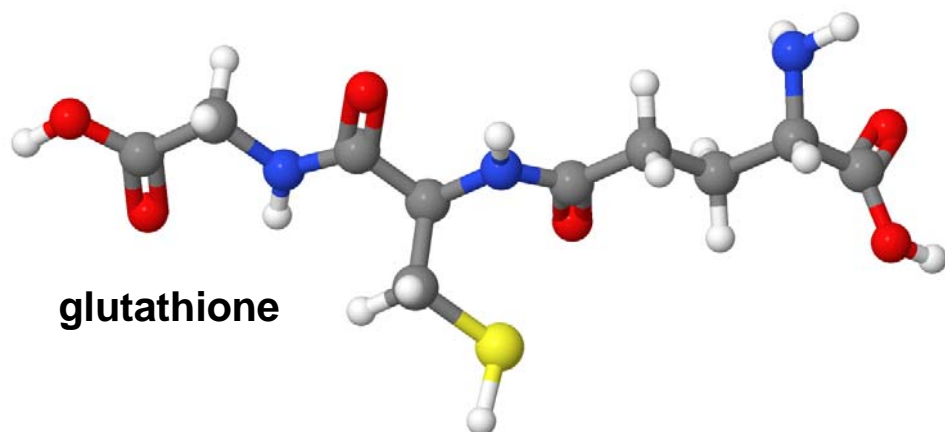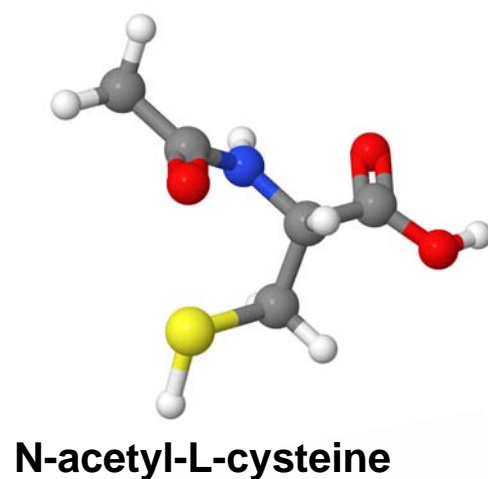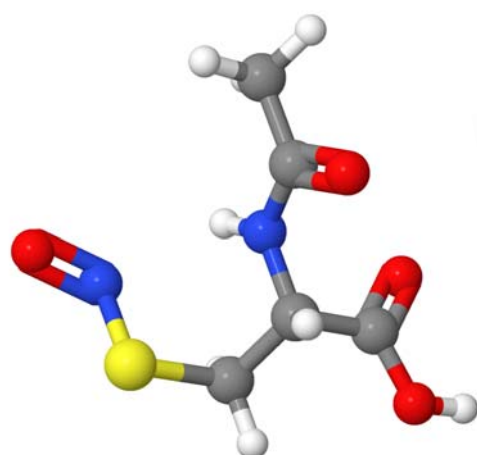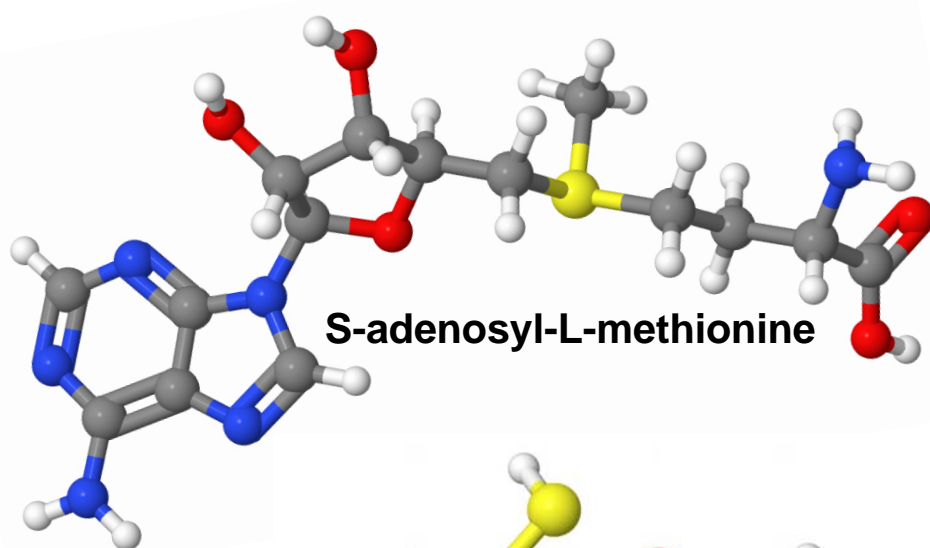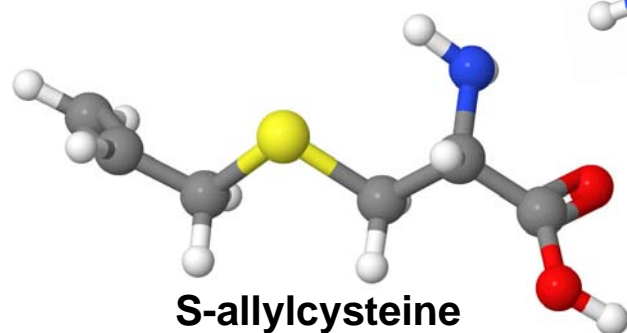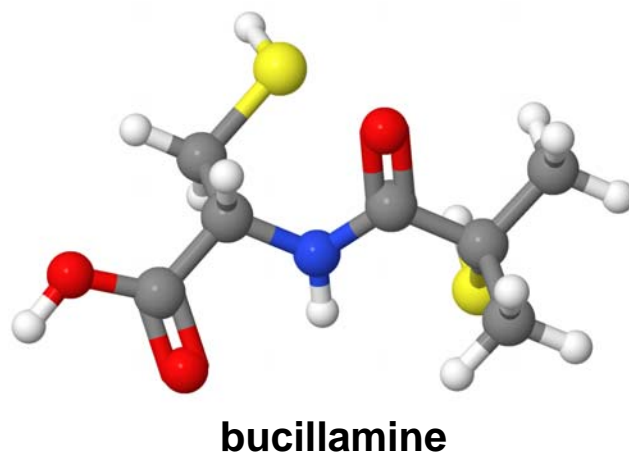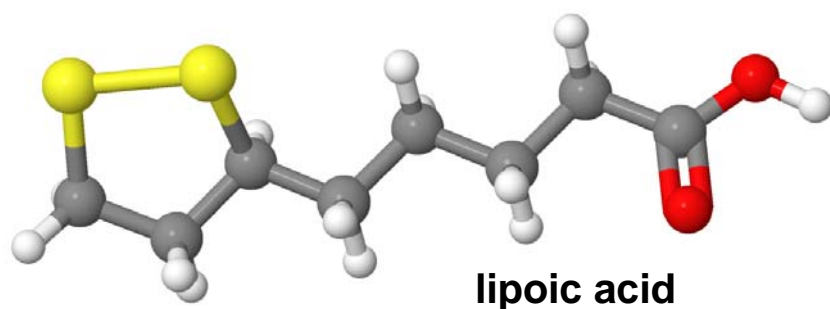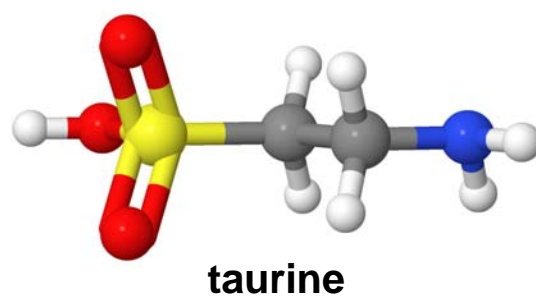

Supplement: Supplementary Figure 1 — Sulfur-containing antioxidants with therapeutic potential in hepatic fibrosis. Glutathione (CAS 70-18-8), N-acetyl-L-cysteine (CAS 616-91-1), S-nitroso-N-acetylcysteine (CAS 56577-02-7), S-adenosyl-L-methionine (CAS 29908-03-0), S-allylcysteine (CAS 21593-77-1), bucillamine (CAS 65002-17-7), lipoic acid (CAS 1200-22-2), and taurine (CAS 107-35-7) were shown to evolve beneficial effects in experimental hepatic fibrosis. [file Image1.PDF]

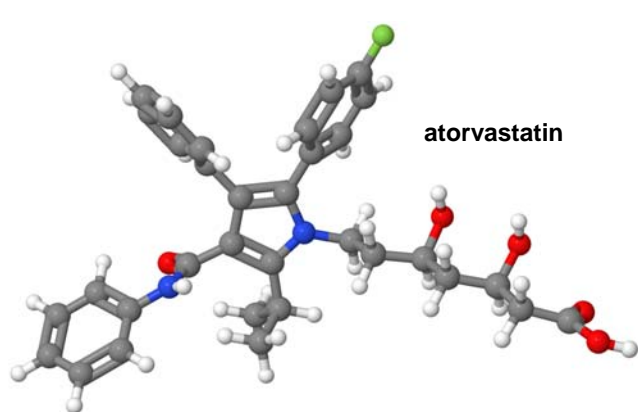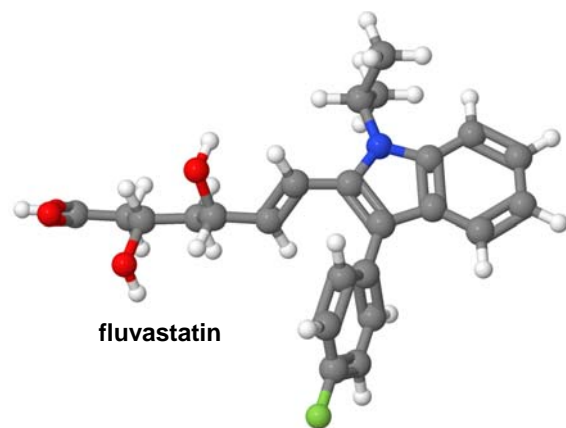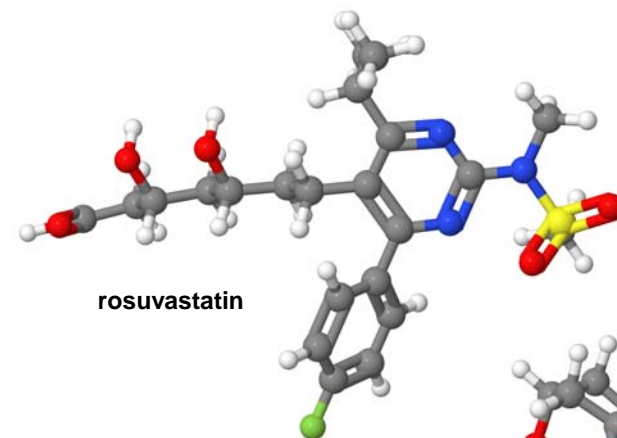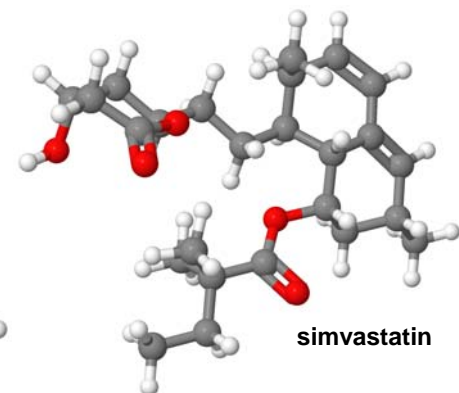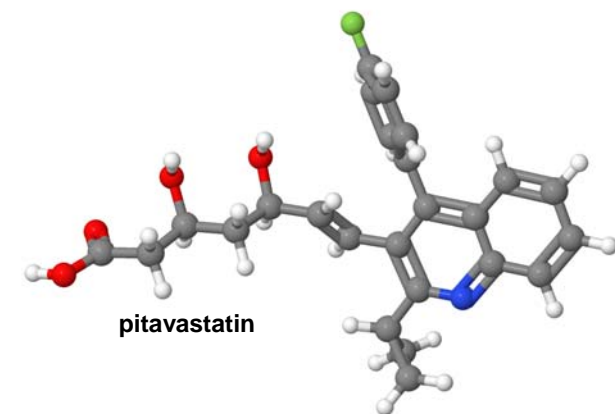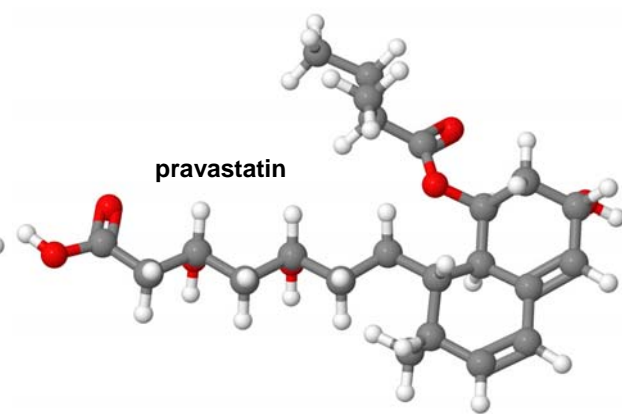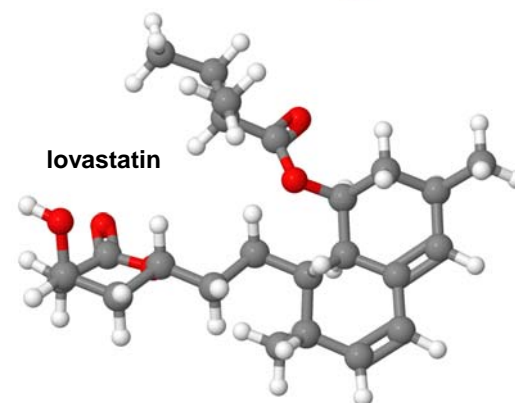

Supplement: Supplementary Figure 3 — HMG-CoA reductase inhibitors. Atorvastatin (CAS 134523-00-5), fluvastatin (CAS 93957-54-1), rosuvastatin (CAS 287714-41-4), pitavastatin (CAS 147511-69-1), pravastatin (CAS 81093-37-0), lovastatin (CAS 75330-75-5), and simvastatin (CAS 79902-63-9) are presentative statins that interfere with hepatic fibrogenesis by inhibiting HMG-CoA reductase thereby preventing formation of mevalonate from HMG-CoA. [file Image3.PDF]

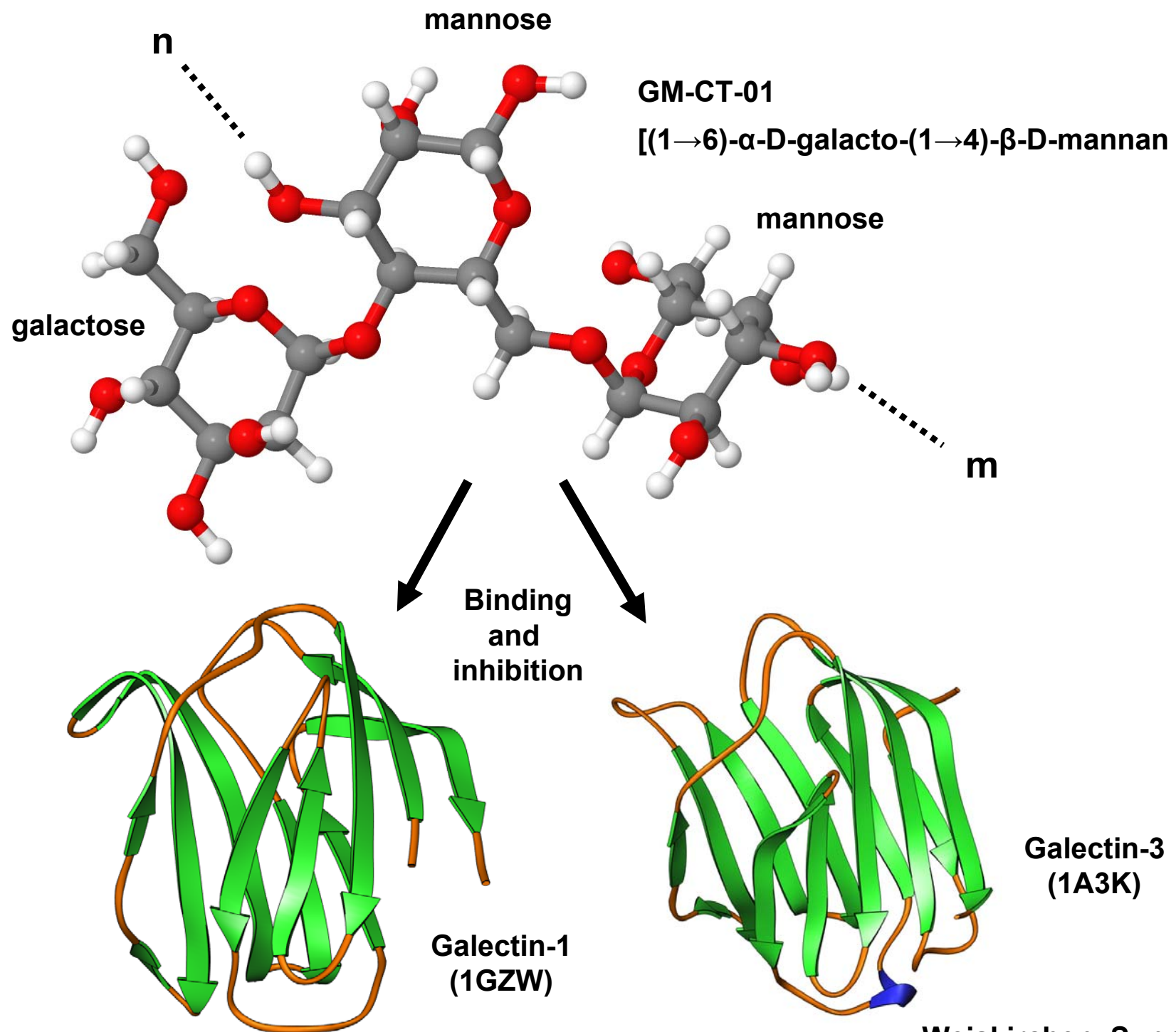

Supplement: Supplementary Figure 5 — Therapeutic polysaccharides targeting galectins. The carbohydrate-based compounds GR-MD-02 and GM-CT-01 (DAVANAT) bind to galectin-1 and galectin-3 thereby preventing its inflammation- and fibrosis-associated chemoattraction. The backbone of GM-CT-01 is a proprietary galactomannan polysaccharide composed of (1 → 4)-linked β-D-mannopyranosyl units, to which single α-D-galactopyranosyl is attached by (1 → 6)-linkage. The mannose to galactose ratio is approximately 1.7 with mean molecular weight of approximately 50 kDa. Likewise, GR-MD-02 is a proprietary galactoarabino-rhamnogalacturonate polymer that is comprised predominantly of 1,4-linked galacturonic acid, with β-galactose and arabinose side chains of approximately 50 kDa (not shown). Both polysaccharides bind with high affinity to galectins, thereby preventing their pro-inflammatory function in recruting inflammatory cells to the diseased liver tissue. The depicted human galectin structures were generated with the Ribbons software (ver 3.0) using the structure coordinates that are deposited in the RCSB Protein Data Bank under accession nos. and 1GZW (galectin-1) and 1A3K (galectin-3), respectively. [file Image5.PDF]

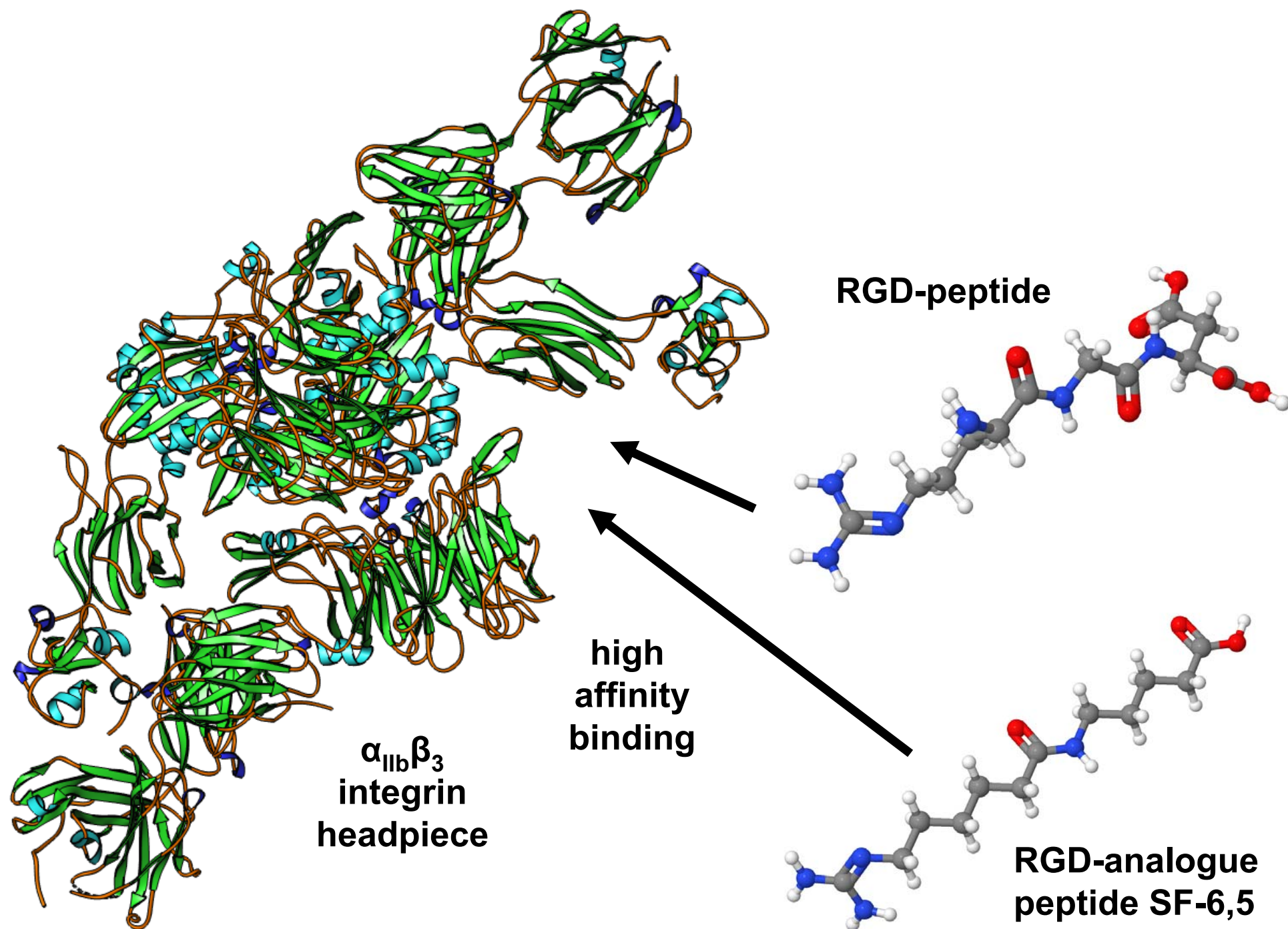

Supplement: Supplementary Figure 6 — RGD-peptide and integrins. Integrin function can be blocked by the tripeptide RGD or substances that have a similar structural arrangement such as the non-peptidic RGD mimetic SF-6,5. Both substances bind with high affinity to regions within integrins. [file Image6.PDF]

**malotilate**

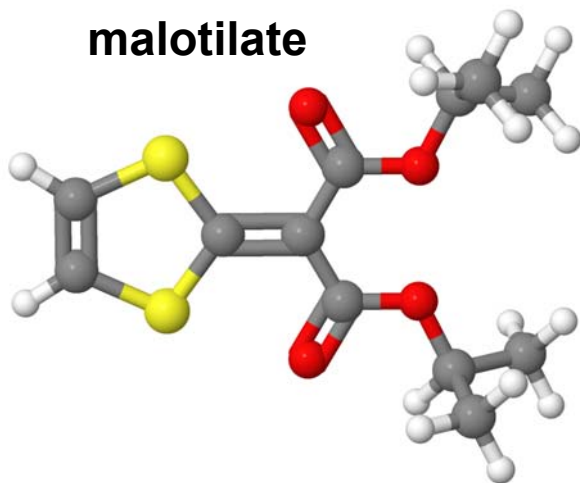

**tranilast**

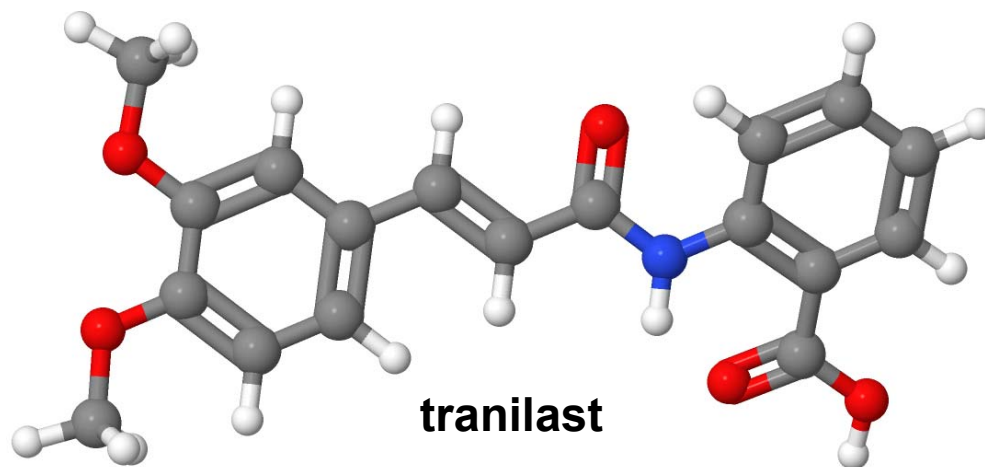

**HOE 077**

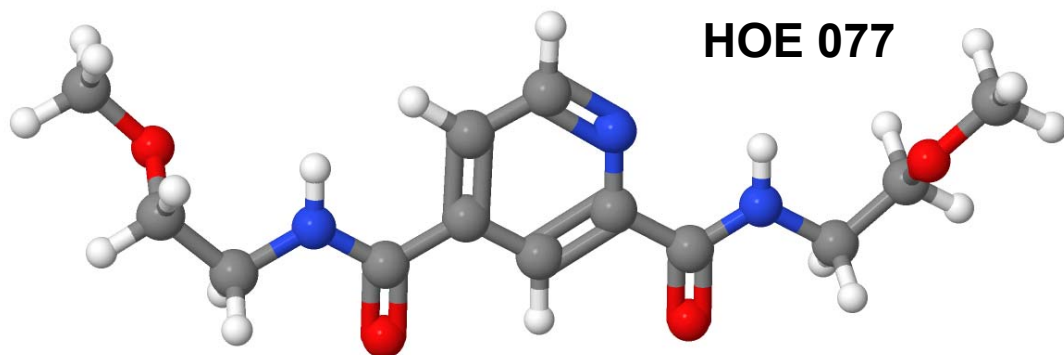

**S 0885**

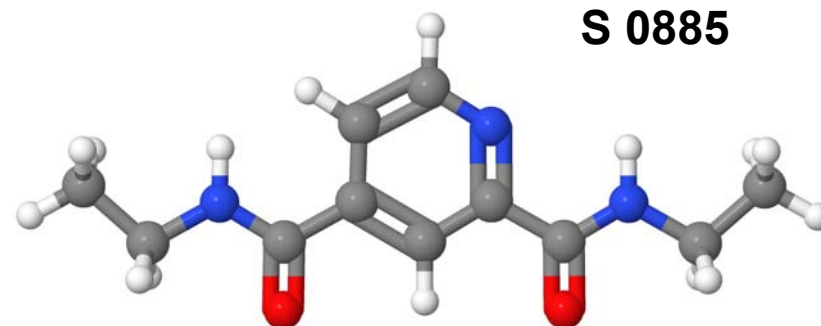

**safironil**

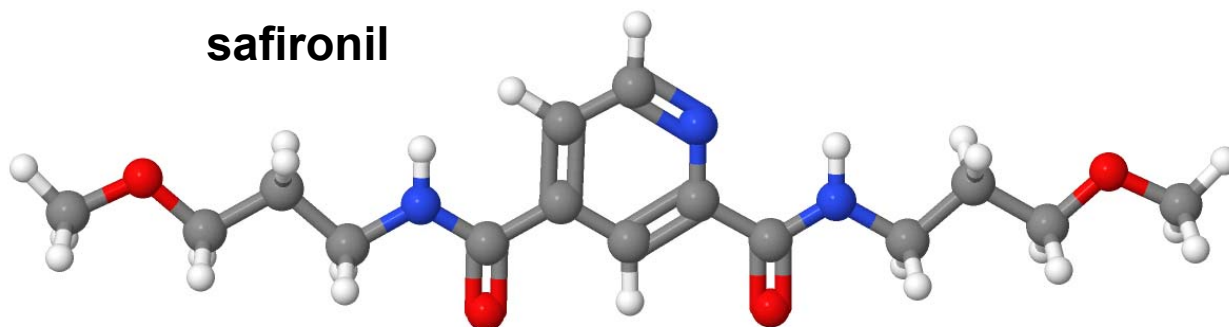

**$\beta$ -aminopropionitrile**

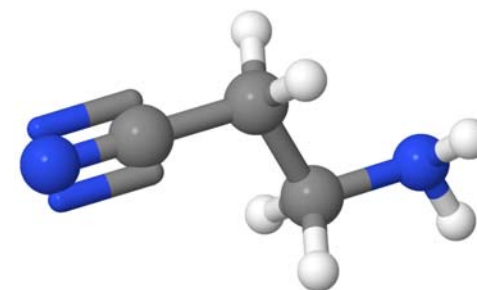

Supplement: Supplementary Figure 7 — Inhibitors of collagen synthesis and fibroblast proliferation. Malotilate (CAS 59937-28-9), transilast (CAS 53902-12-8), HOE 077 (CAS 128075-79-6), S 0885 (CAS 117517-22-3) β-aminopropionitrile (CAS 151-18-8), and safironil (CAS 134377-69-8) are drugs that interfere with fibroblastic proliferation and expression or synthesis of collagen. [file Image7.PDF]

**perindopril**

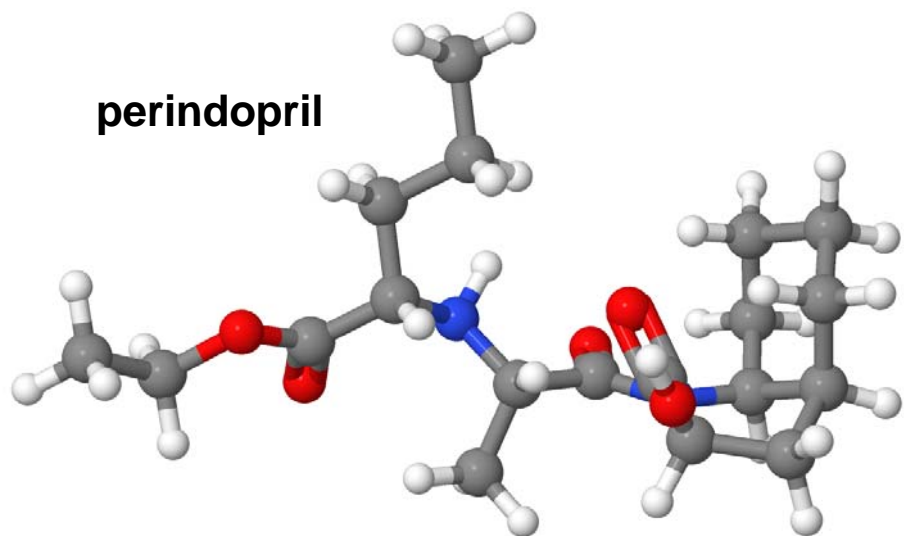

**candesartan**

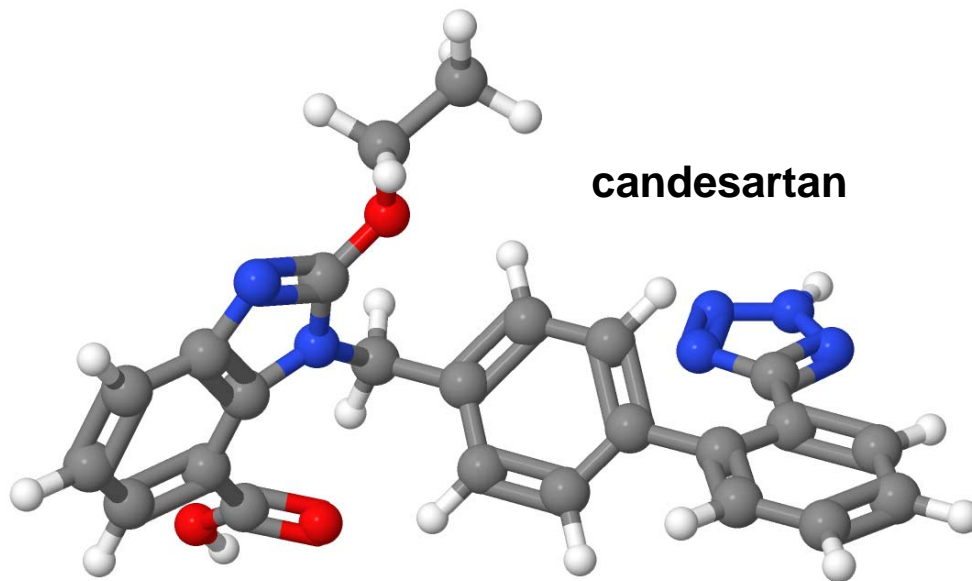

**losartan**

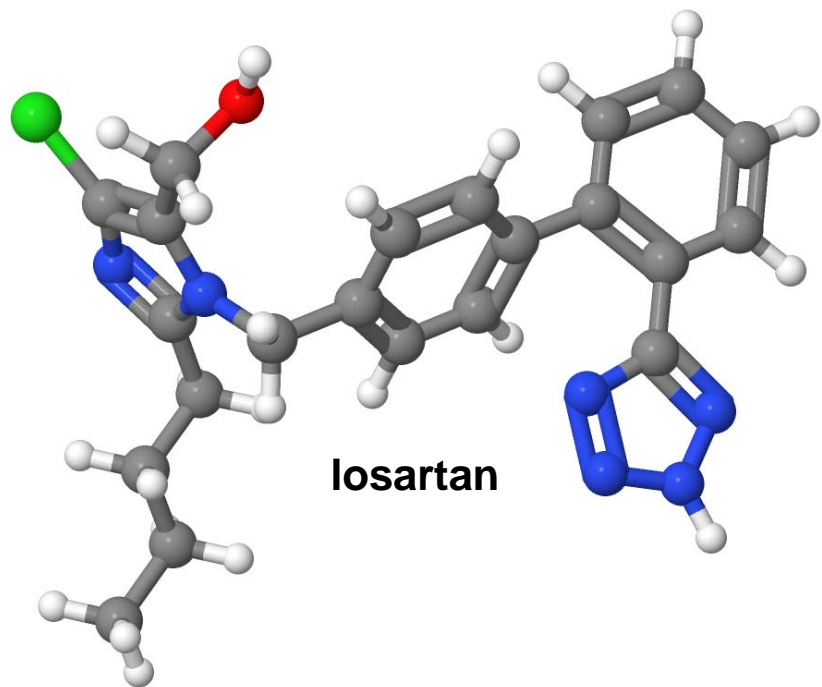

**aliskiren**

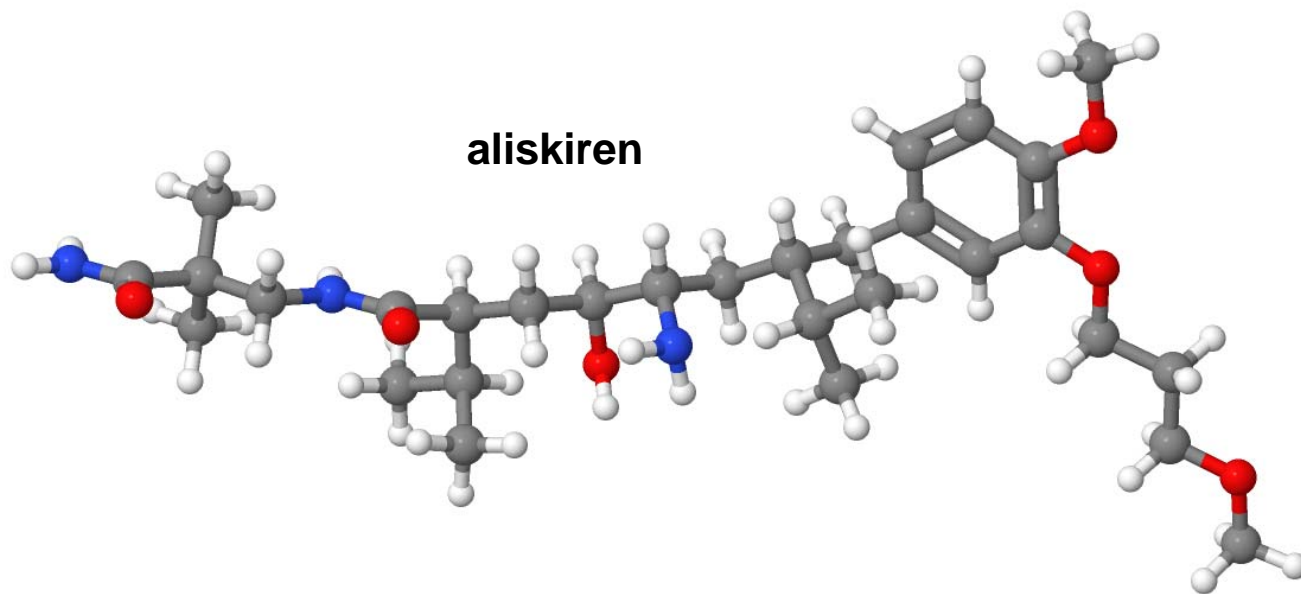

Supplement: Supplementary Figure 9 — Angiotensin receptor antagonism. The Angiotensin axis can be blocked by ACE inhibitors (e.g., perindopril, CAS 107133-36-8), selective receptor blockers (losartan, CAS 114798-26-4; candesartan, CAS 139481-59-7), or direct renin inhibitors (e.g., aliskiren; CAS 173334-57-1). [file Image9.PDF]

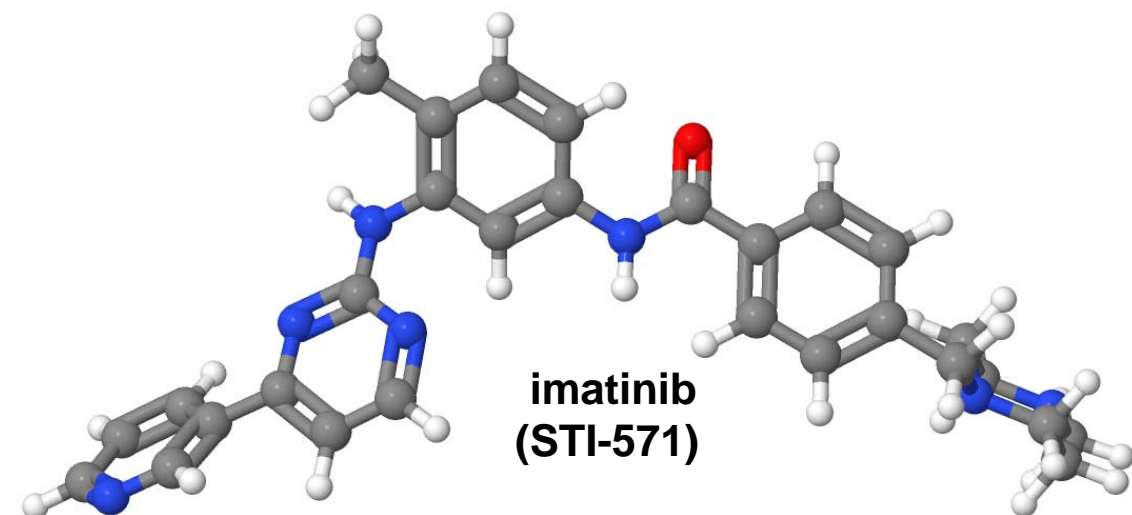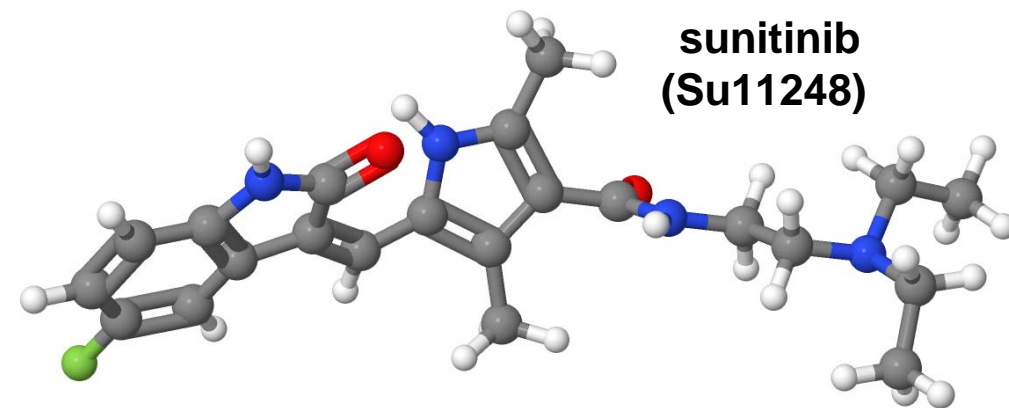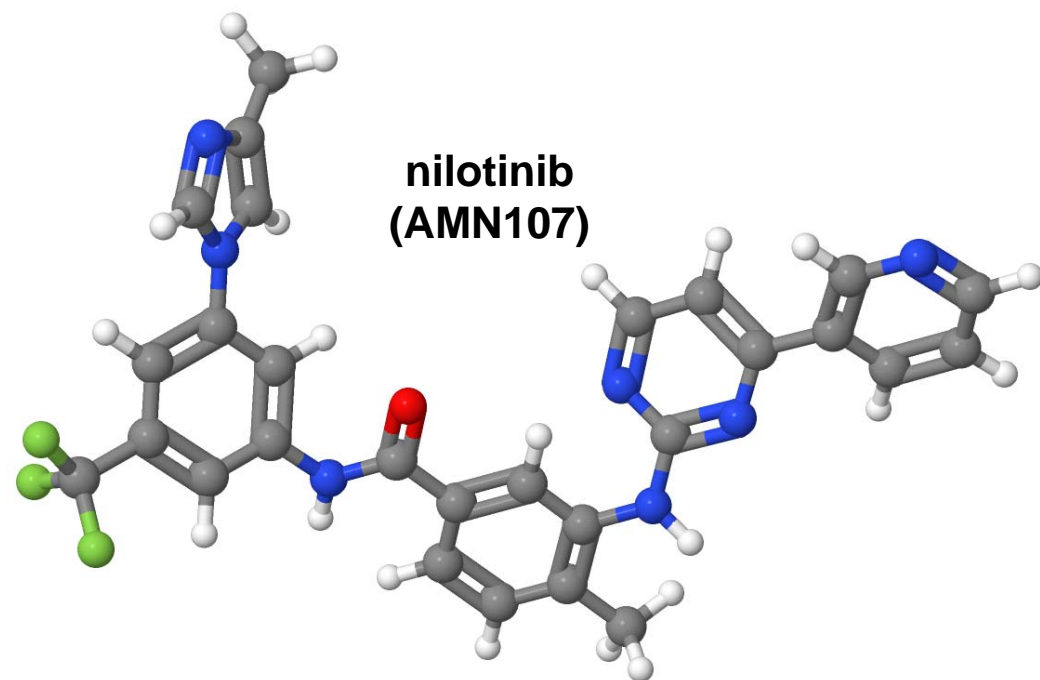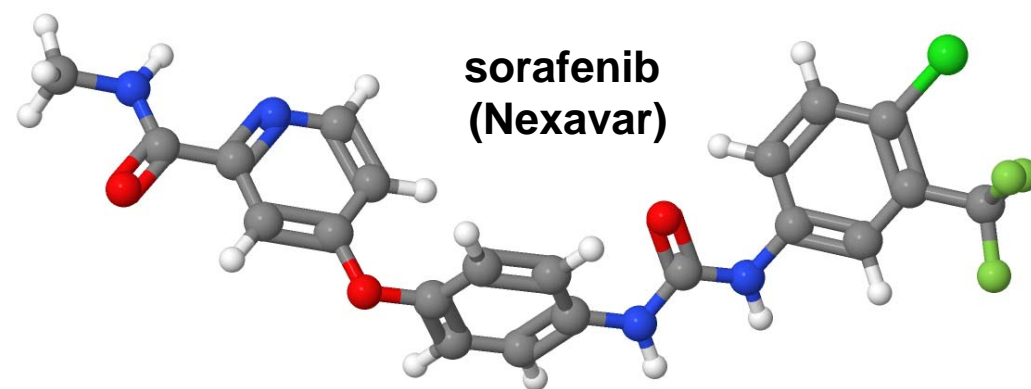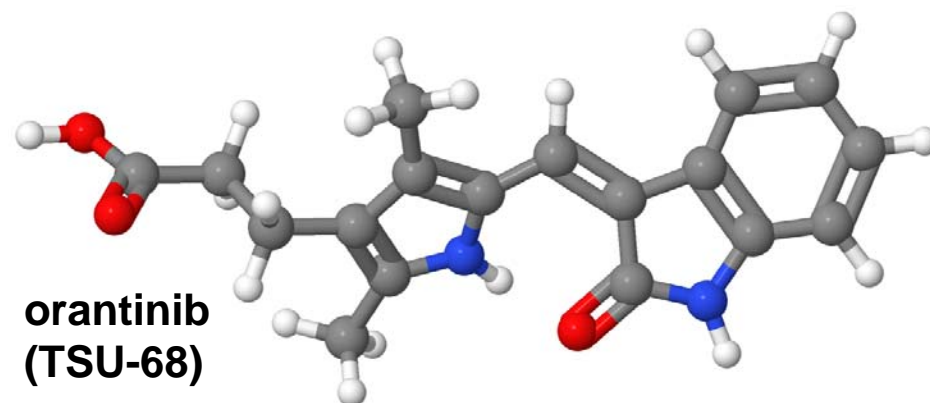

Supplement: Supplementary Figure 11 — RTK inhibitor substances. Numerous studies demonstrated that Imatinib (CAS 152459-95-5), nilotinib (CAS 641571-10-0), sunitinib (CAS 557795-19-4), sorafenib (CAS 284461-73-0), and orantinib (CAS 252916-29-3) have beneficial activities in hepatic fibrosis by targeting diverse receptor kinases (for details see text). [file Image11.PDF]

**vitamin D**

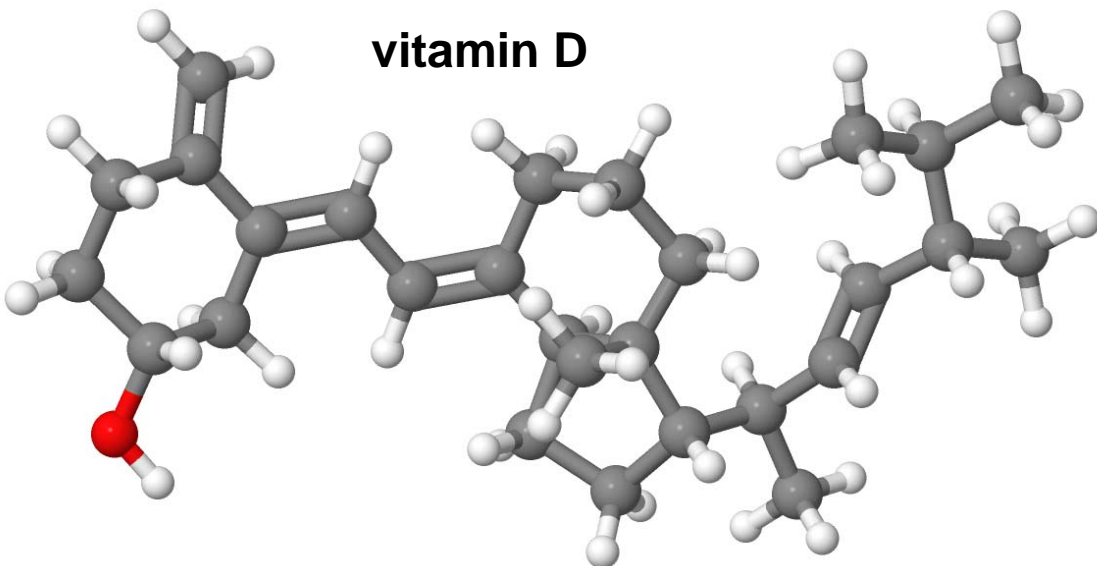

**tetrandrine**

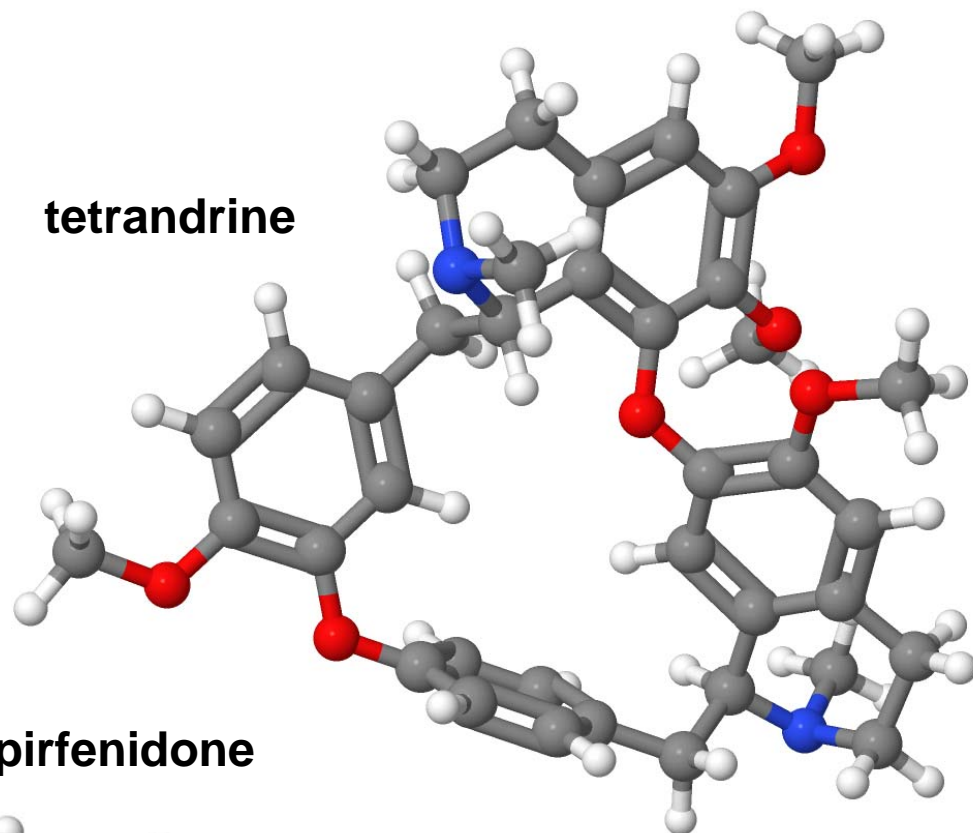

**pirfenidone**

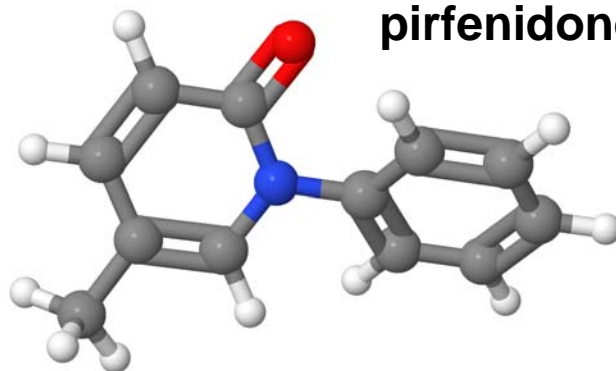

**caffeine**

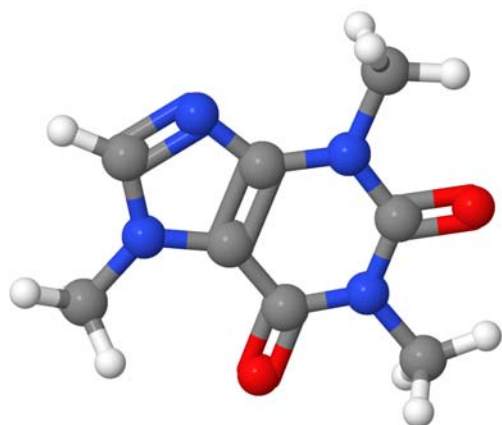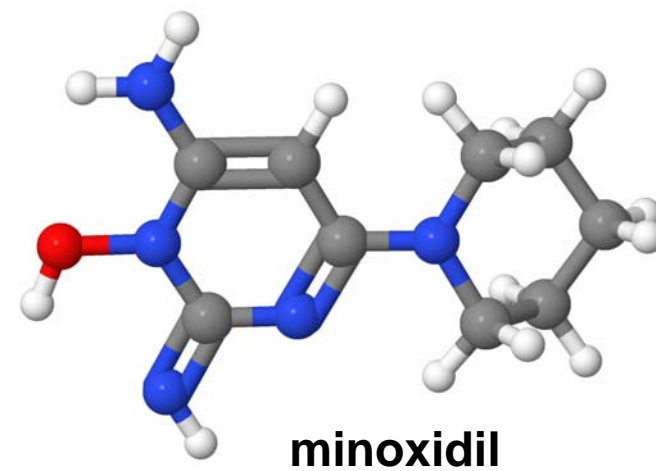

**minoxidil**

Supplement: Supplementary Figure 12 — Miscellaneous compounds with hepatoprotective, anti-inflammatory or anti-fibrotic effects. Vitamin D (CAS 67-97-0), tetrandrine (CAS 518-34-3), caffeine (CAS 58-08-2), pirfenidone (CAS 53179-13-8), and minoxidil (CAS 38304-91-5) are compounds that showed anti-inflammatory and anti-fibrotic activities in various fibrosis models. [file Image12.PDF]

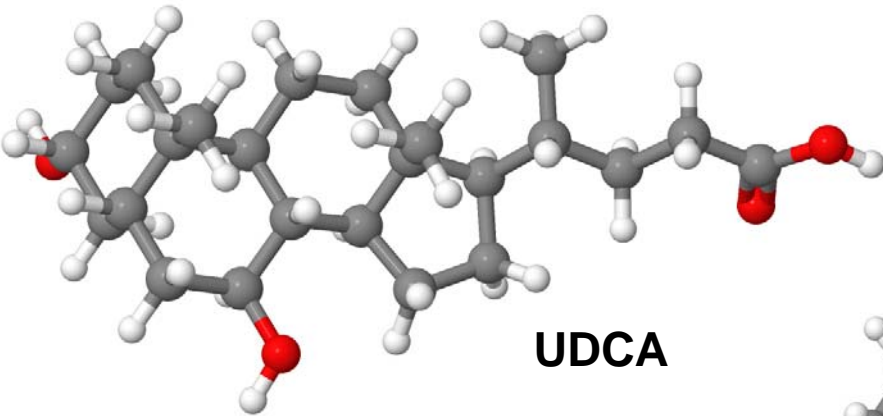

**Obeticholic acid  
(INT-747)**

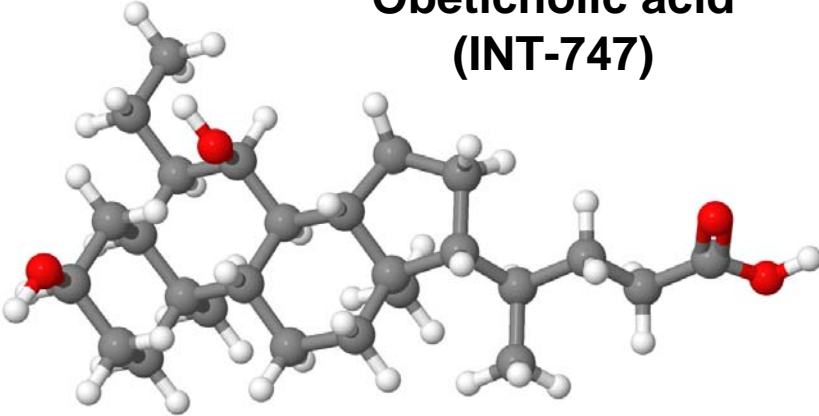

**cholic acid**

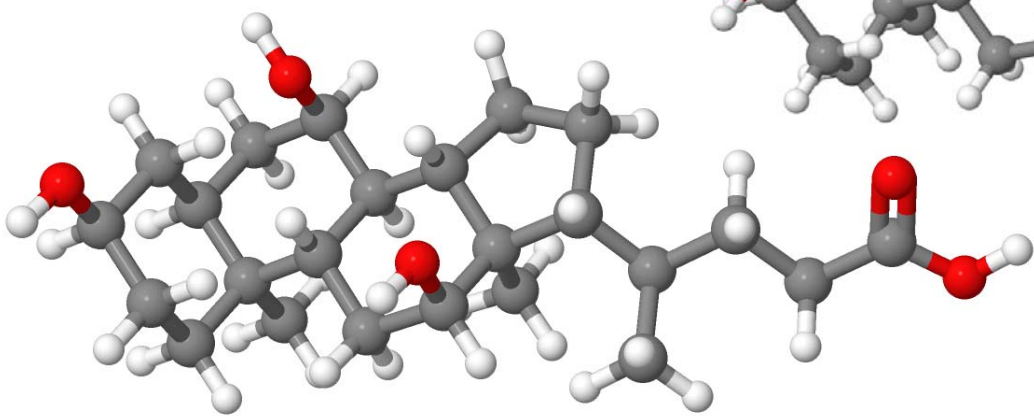

**lithocholic acid**

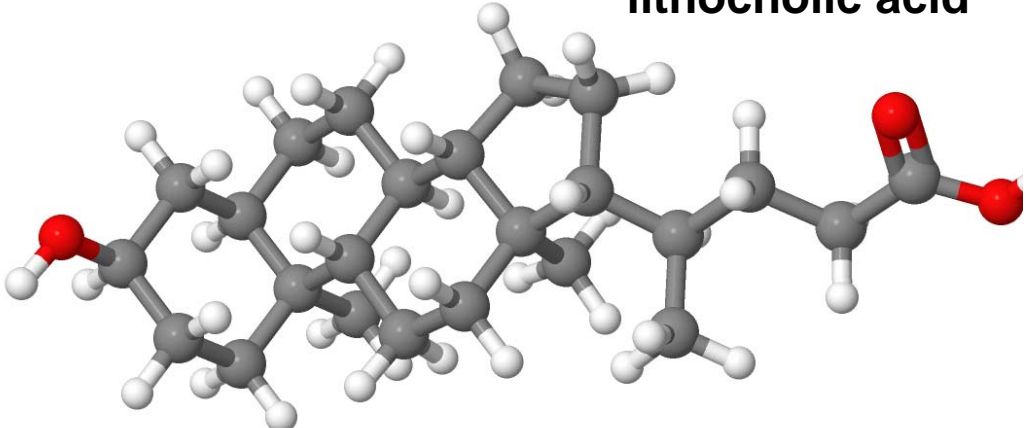

**deoxycholic acid**

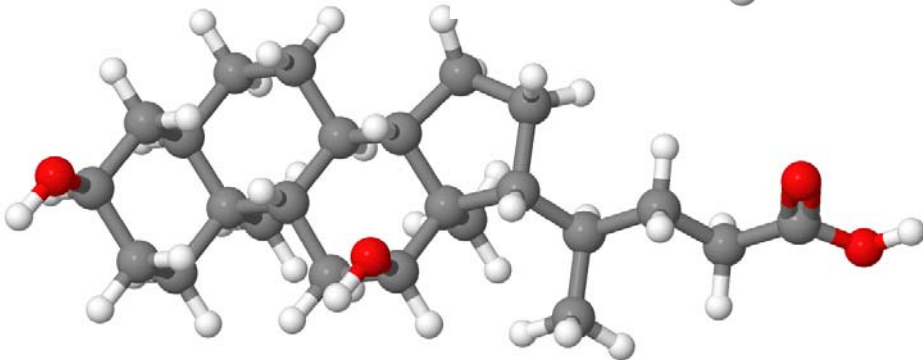

Supplement: Supplementary Figure 13 — Bile acids and FXR antagonists. Obeticholic acid (OCA, CAS 459789-99-2) is a semi-synthetic bile acid derivate related to Ursodeoxycholate (UDCA, CAS 128-13-2) used to treat cholestatic liver disease. In contrast to UDCA, OCA and other bile acids (cholic acid, lithocholic acid, and deoxycholic acid) are FXR ligands. [file Image13.PDF]
