# Supplementary material for: Hepatoprotective and Anti-fibrotic Agents: It's Time to Take the Next Step
Source: Front Pharmacol. 2016 Jan 7;6:303. doi: 10.3389/fphar.2015.00303 (PMC4703795; doi:10.3389/fphar.2015.00303)
Supplement: Supplementary Figure 2 — The Nicotinamide adenine dinucleotide phosphate oxidases (NOXs) and their inhibition. Nicotinamide (CAS 98-92-0) and nicotinic acid (CAS 59-67-6) serve as nicotinamide adenine dinucleotide precursors. NADPH oxidases (NOXs) catalyzes the transfer of electrons form NADPH to molecular oxygen thereby producing superoxide and other ROS. GKT137831 is a dual inhibitor showing high affinity for NOX1 and NOX4 but not for other isoforms, while decursin (CAS:5928-25-6) was found experimentally to suppress NOX1, NOX2 and NOX4. [file Image2.PDF]

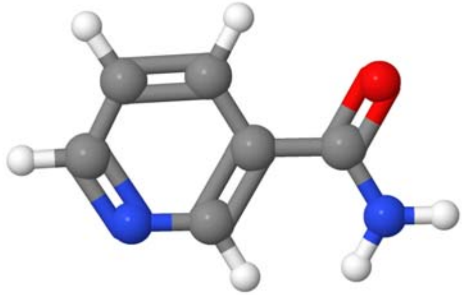

nicotinamide (vitamin B3)

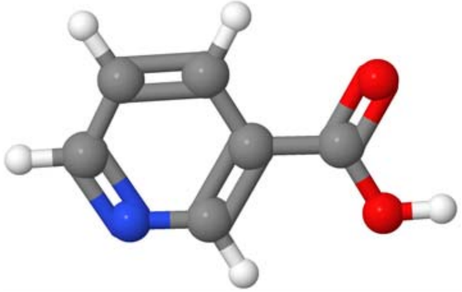

nicotinic acid (niacin)

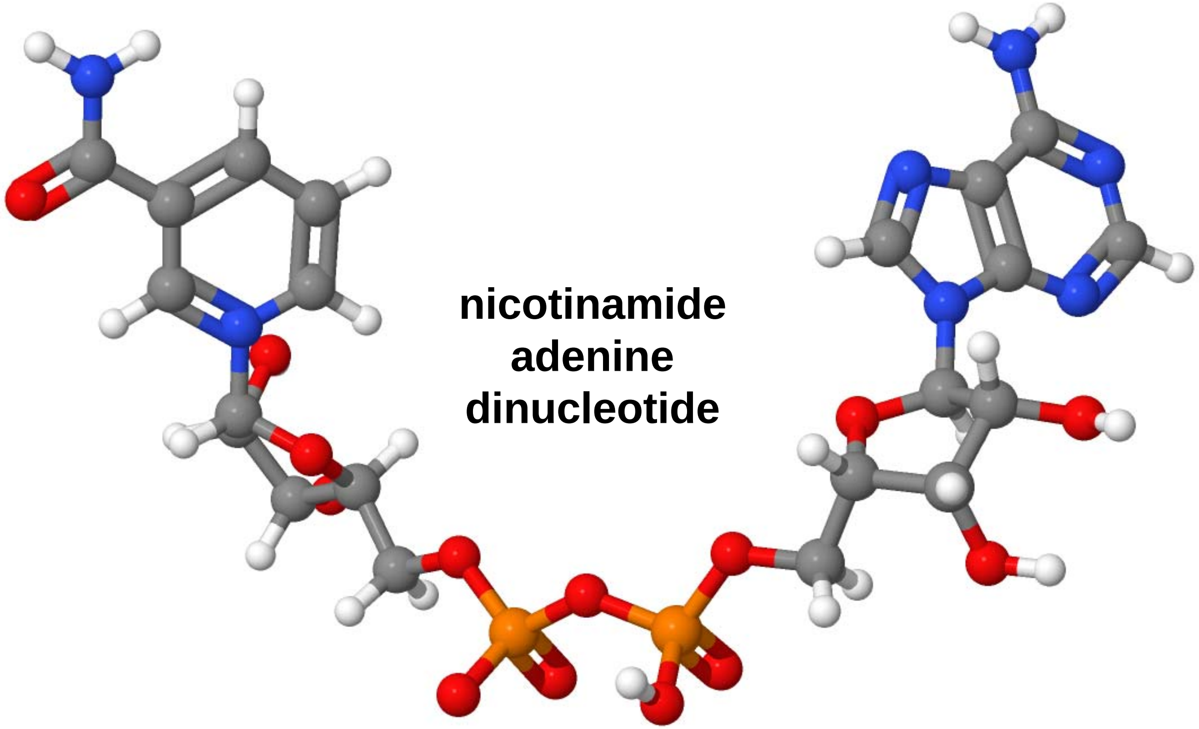

nicotinamide adenine dinucleotide

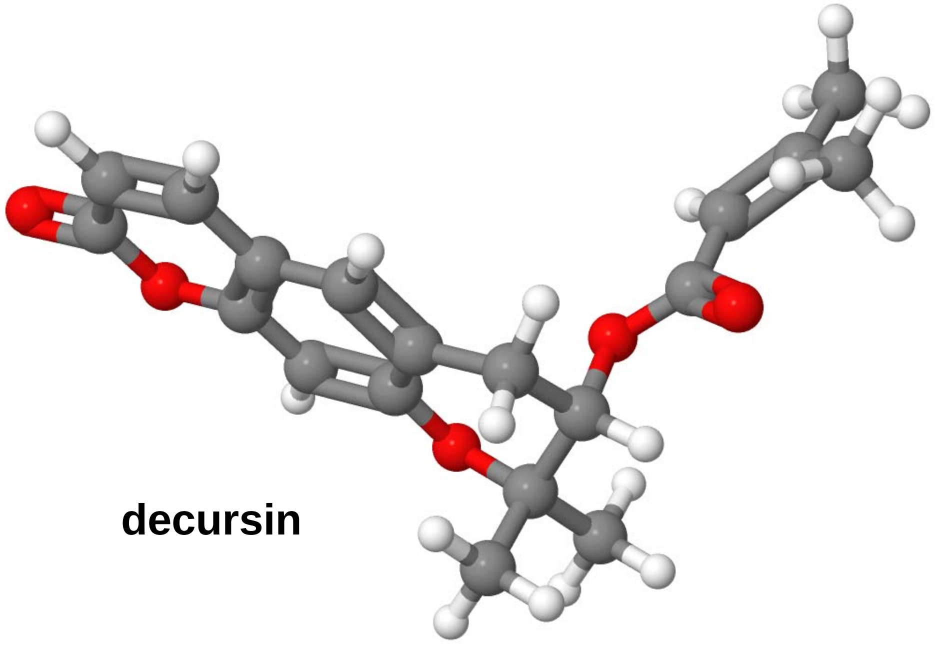

decursin

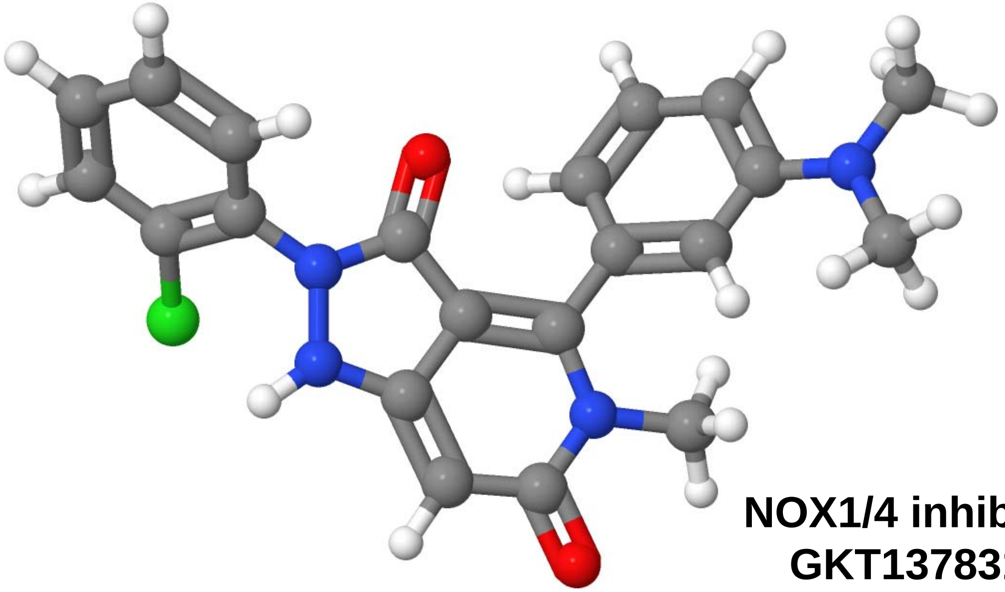

NOX1/4 inhibitor GKT137831

Weiskirchen, Suppl. Figure 2
