# Supplementary material for: Hepatoprotective and Anti-fibrotic Agents: It's Time to Take the Next Step
Source: Front Pharmacol. 2016 Jan 7;6:303. doi: 10.3389/fphar.2015.00303 (PMC4703795; doi:10.3389/fphar.2015.00303)
Supplement: Supplementary Figure 4 — Thiazolidinediones in liver healthy. All members of this class (e.g., pioglitazone, CAS 111025-46-8; rosiglitazone, CAS 122320-73-4) are derivatives of the parent compound 2,4-thiazolidinedione (CAS 2295-31-0). These compounds that are also known as glitazones activate nuclear receptors (PPARs) that lead to modulation of target gene transcription. [file Image4.PDF]

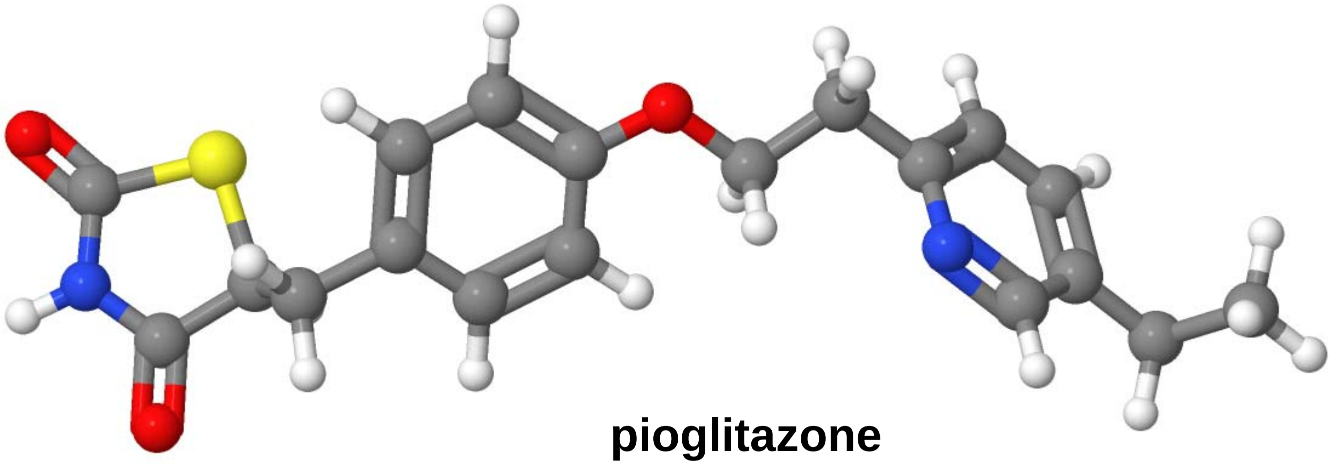

pioglitazone
(Actos)

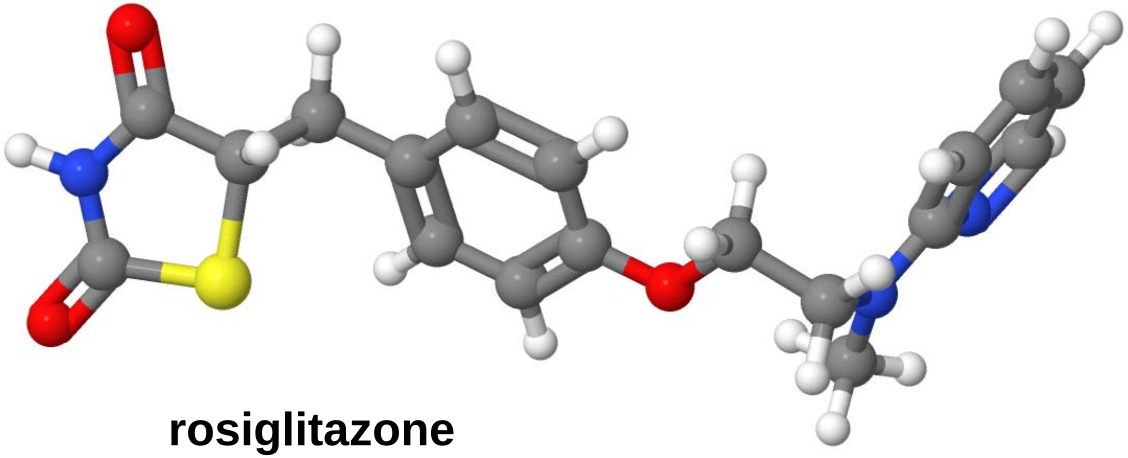

rosiglitazone
(Avandia)

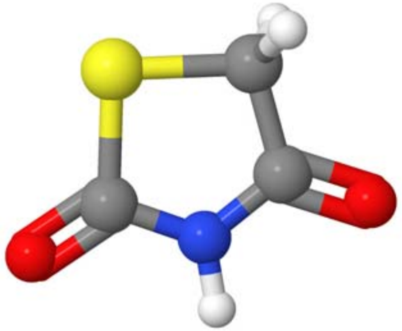

thiazolidinedione
(“glitazone“)

Weiskirchen, Suppl. Figure 4
