# Supplementary material for: Hepatoprotective and Anti-fibrotic Agents: It's Time to Take the Next Step
Source: Front Pharmacol. 2016 Jan 7;6:303. doi: 10.3389/fphar.2015.00303 (PMC4703795; doi:10.3389/fphar.2015.00303)
Supplement: Supplementary Figure 8 — Histone deacetylase and acetyltransferase blockers. The curcuma longa ingredient curcumin (CAS 458-37-7), trichostatin A (TSA, CAS 58880-19-6), and valproic acid (CAS 99-66-1) marked under a multitude of brand names are potent histone deacetylase blocker, while the green tea ingredient epigallocatechin-3-gallate (EGCG, CAS 989-51-5) interferes with the activity of acetyltransferases. [file Image8.PDF]

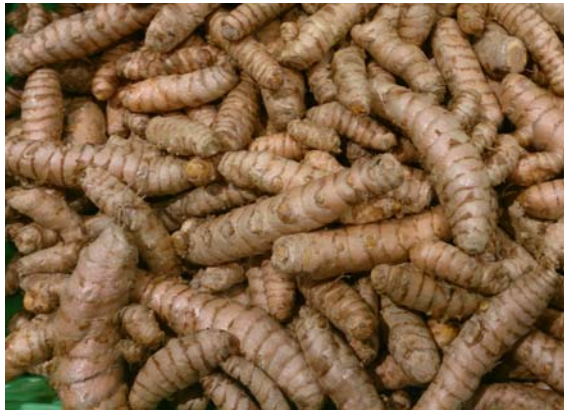

Curcuma longa (roots)

curcumin

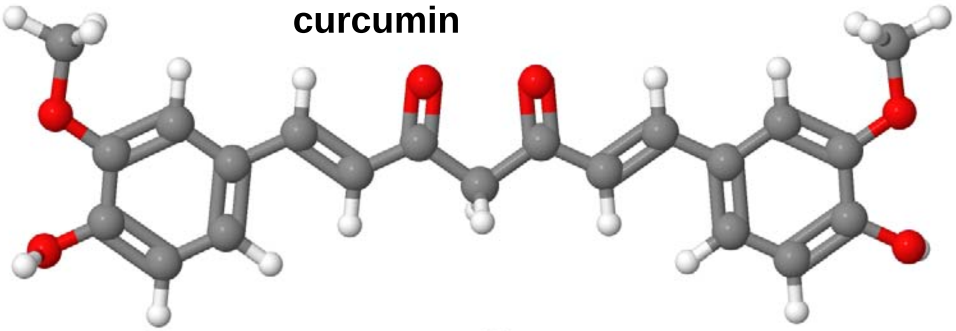

epigallocatechin-3-gallate (EGCG)

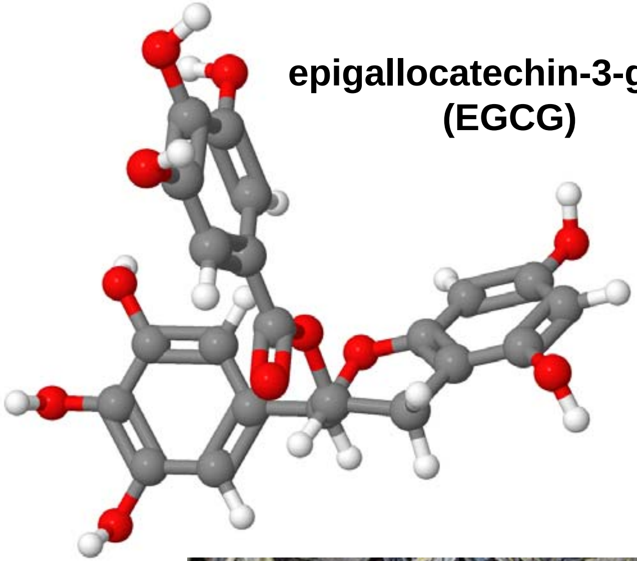

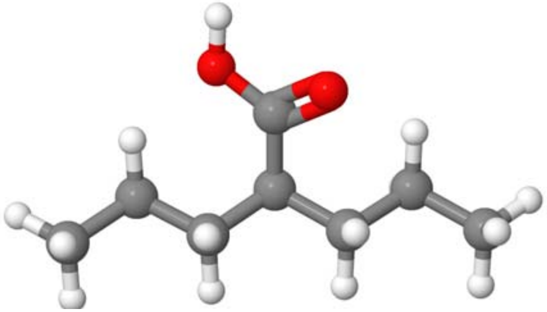

valproic acid
“Convulex“
“Depakote“
“Epilim“
“Valparin“
“Valpro“
“Vilapro“
“Stavzor“

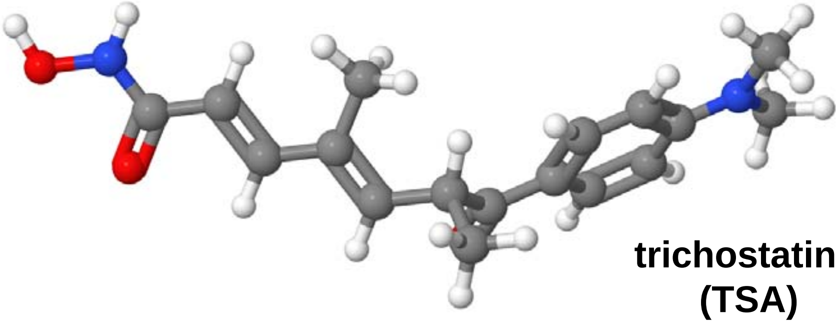

trichostatin A (TSA)

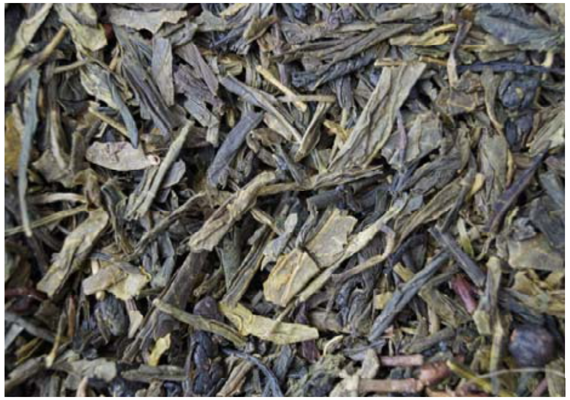

Japanese green tea (*sencha*)

Weiskirchen, Suppl. Figure 8
