# Supplementary material for: Hepatoprotective and Anti-fibrotic Agents: It's Time to Take the Next Step
Source: Front Pharmacol. 2016 Jan 7;6:303. doi: 10.3389/fphar.2015.00303 (PMC4703795; doi:10.3389/fphar.2015.00303)
Supplement: Supplementary Figure 10 — Endothelin-1. ET-1 (CAS 117399-94-7) is a potent vasoconstrictor involved in the formation of portal hypertension. The major biological active peptide consists of 21 residues. It is stepwise processed from a 212 aa precursor form. The mature peptide contains two disulfide bonds (disulfide bridges: 1-15 and 3-11). The X-ray structure of mature human ET-1 that is deposited in the RCSB Protein Data Base under accession no. 1EDN shows that the entire carboxy terminal tail (residues 16-21) that is crucial for receptor binding and vasoactivity foms a helix. [file Image10.PDF]

Human endothelin-1 (ET-1)

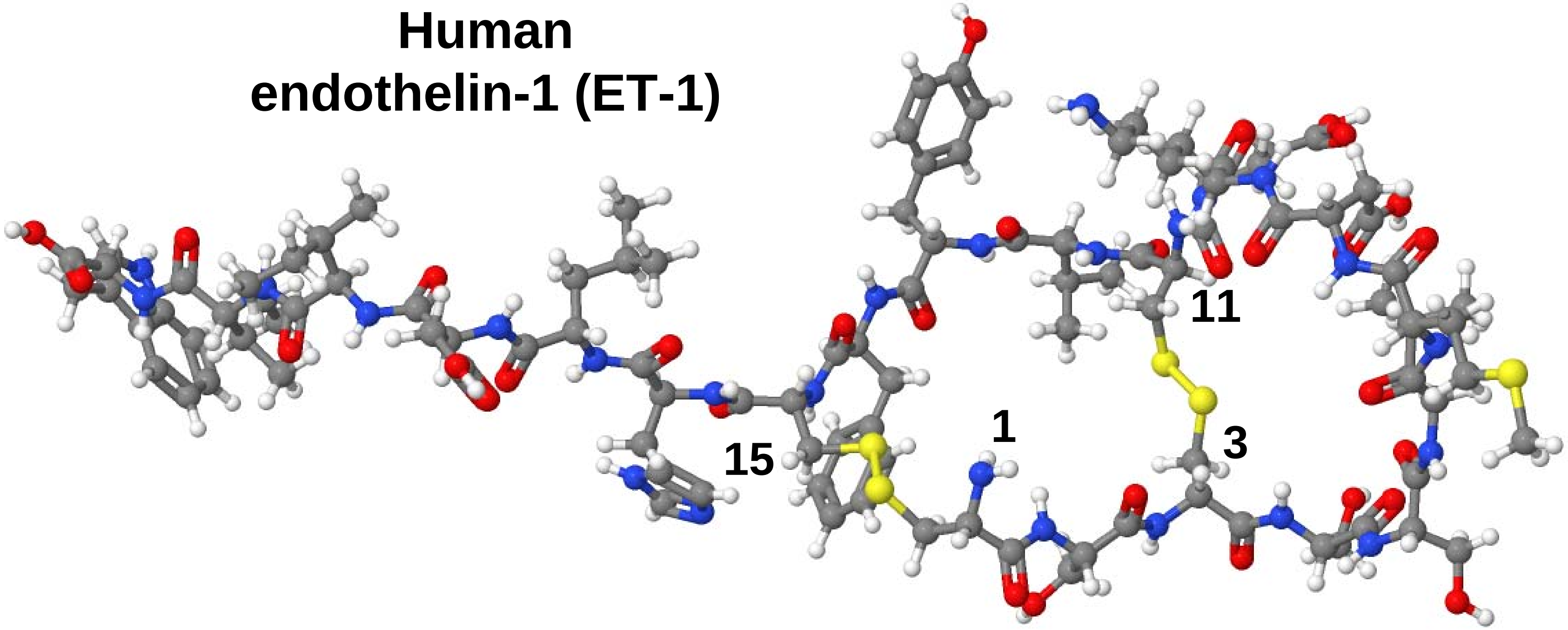

ET-1 (1EDN)

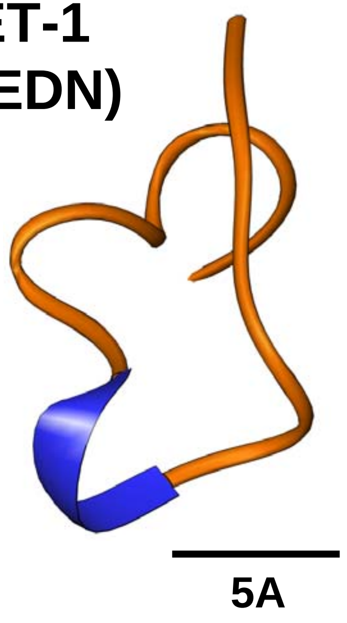

3 — 11

1

Cys-Ser-Cys-Ser-Ser-Leu-Met-Asp-Lys-Glu-Cys-Val-Tyr-Phe-

Cys-His-Leu-Asp-Ile-Ile-Trp

15

21 aa small ET-1

proteolytic processing

```
  1 MDYLLMIfSL LfVACQGAPE TAVLGAELSA VGENGGEKPT PSPPWRLRRS KRCSCSSLMD
 61 KECVYfCHLD IIWVNTPEHV VPYGLGSPRS KRALENLLPT KATDRENRCQ CASQKDKKCW
121 NfCQAGKELR AEDIMEKDWN NHKKGKDCSK LGKKCIYQQL VRGRKIRRSS EEHLRQTRSE
181 TMRNSVKSSf HDPKLKGNPS RERYVTHNRA HW
```

AAH09720

Weiskirchen, Suppl. Figure 10
